# Characterization of Carbapenemase- and ESBL-Producing Gram-Negative Bacilli Isolated from Patients with Urinary Tract and Bloodstream Infections

**DOI:** 10.3390/antibiotics12091386

**Published:** 2023-08-30

**Authors:** Isabella A. Tickler, Diane Kawa, Anne E. Obradovich, Ferric C. Fang, Fred C. Tenover, the Healthcare Associated Infections Consortium

**Affiliations:** 1 Cepheid, Sunnyvale, CA 94089, USA; 2 Department of Medical Microbiology and Immunology, School of Medicine, Creighton University, Omaha, NE 68178, USA; 3 Departments of Laboratory Medicine and Microbiology, University of Washington, Seattle, WA 98195, USA; 4 College of Arts & Sciences, University of Dayton, Dayton, OH 45469, USA

**Keywords:** carbapenemases, ESBL, AmpC, antimicrobial resistance, susceptibility testing

## Abstract

A total of 199 Gram-negative bacterial isolates from urinary tract infections and 162 from bloodstream infections were collected from 12 healthcare systems throughout the United States between May 2021 and August 2022. The isolates, phenotypically non-susceptible to 2nd or 3rd generation cephalosporins or carbapenems, were characterized through antimicrobial susceptibility testing and whole genome sequence analysis to obtain a broad snapshot of beta-lactamase-mediated resistance among these two sample types. Overall, 23 different carbapenemase genes were detected among 13 species (20.5% of isolates). The *bla*_KPC-3_ and *bla*_KPC-2_ subtypes were the most common carbapenemase genes identified, followed by *bla*_NDM_ and the co-carriage of two different *bla*_OXA_ carbapenemases by *Acinetobacter baumannii* isolates. All carbapenemase-producing *A. baumannii* isolates were mCIM negative. Extended-spectrum beta-lactamase genes were identified in 66.2% of isolates; *bla*_CTX-M-15_ was the most common. AmpC genes, both plasmid and chromosomal, were detected in 33.2% of isolates. Importantly, 2.8%, 8.3%, and 22.2% of *bla*_KPC_-positive organisms were susceptible to ertapenem, imipenem, and meropenem, respectively. The correlation between broth microdilution and disk diffusion results was high for most drugs except cefepime, where the detection of resistance was statistically lower by disk diffusion. Thus, there were gaps in the accuracy of susceptibility testing for some mechanisms of resistance.

## 1. Introduction

Antimicrobial-resistant Gram-negative bacteria, especially those producing extended-spectrum beta-lactamases (ESBL) or carbapenemases, have been recognized as urgent global public health threats [1,2]. Such organisms increased in prevalence during the COVID-19 pandemic due to the broad use of antimicrobial agents and infection control measures that focused more on halting the spread of SARS-CoV-2 in hospitals than on traditional healthcare-associated pathogens [3,4]. In fact, the World Health Organization (WHO) has placed carbapenem-resistant (CR) *Acinetobacter baumannii*, CR *Pseudomonas aeruginosa*, CR Enterobacterales, and Enterobacterales resistant to third-generation cephalosporins on the critical priority list for antimicrobial development due to the limited therapeutic options for infections caused by these organisms [5]. Despite the impact on the selection of therapeutic choices for infections with antimicrobial-resistant organisms, surveillance for these organisms in various regions of the United States is often spotty [6]. Thus, there is a need for additional data about the prevalence, genotypes, and extended susceptibility profiles of ESBL- and carbapenemase-producing organisms [7]. Multicenter surveys of beta-lactam resistance in Gram-negative bacteria in the U.S. have largely been limited to specific resistance mechanisms [8], selected bacterial species [9,10], or the pre-COVID period [11]. This study is intended to address this gap.

The goals of this study were to use whole genome sequencing to identify the genes encoding ESBLs, carbapenemases, and other beta-lactamases in Gram-negative bacilli obtained from patients with urinary tract infections and bloodstream infections in the United States, to compare the resistance determinants in the organisms from the two specimen types, and to investigate emerging mechanisms of beta-lactam resistance. 

## 2. Results

### 2.1. Description of the Isolates

**Figure 1:**
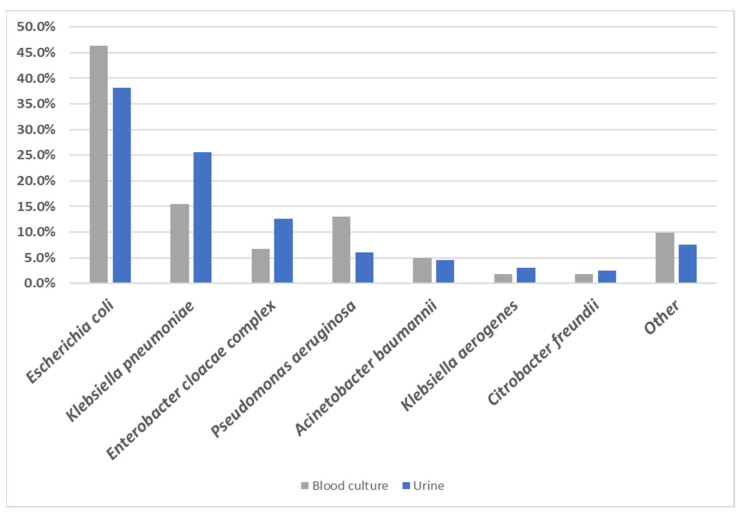
Prevalence of the main bacterial species in blood and urine culture specimens. Isolates identified by sequence analysis to the subspecies level as *Klebsiella pneumoniae* subsp. *pneumoniae* and several *Enterobacter* spp. were grouped as *K. pneumoniae* and *E. cloacae* complex, respectively, for the purpose of this chart; see Table S1 for details.

There were 361 isolates (199 urine isolates and 162 blood culture isolates) characterized in this study. *E. coli* represented 46.3% of isolates from blood cultures, compared to 38.2% of isolates from urine cultures, while *K. pneumoniae* represented only 15.4% of blood culture isolates but 25.6% of urine isolates (Figure 1). *P. aeruginosa* was isolated from 13.0% of blood cultures compared to only 6.0% from urine, while isolates of *Enterobacter cloacae* complex were isolated from 6.8% of blood culture samples but from 12.6% of urine cultures (Figure 1).

**Figure 2:**
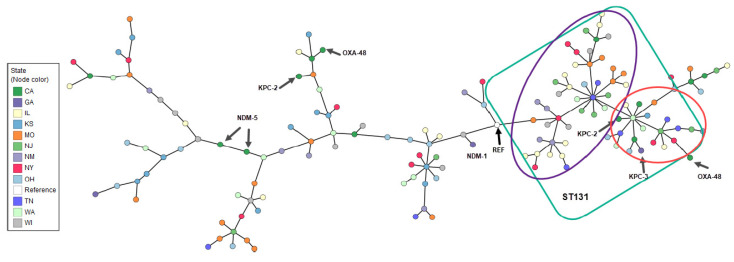
Minimum Spanning Tree based on MLST of *Escherichia coli* by U.S. state of isolation and carbapenemase gene identification. Arrows indicate isolates that harbor a carbapenemase gene. The green square shows the ST131 lineage. The purple circle delineates strains harboring *bla*_CTX-M-15_; the red circle shows the strains harboring mostly *bla*_CTX-M-14_ and *bla*_CTX-M-27._ Reference strain (REF): NZ_CP117235, *Escherichia coli* strain ATCC25922.

**Figure 3:**
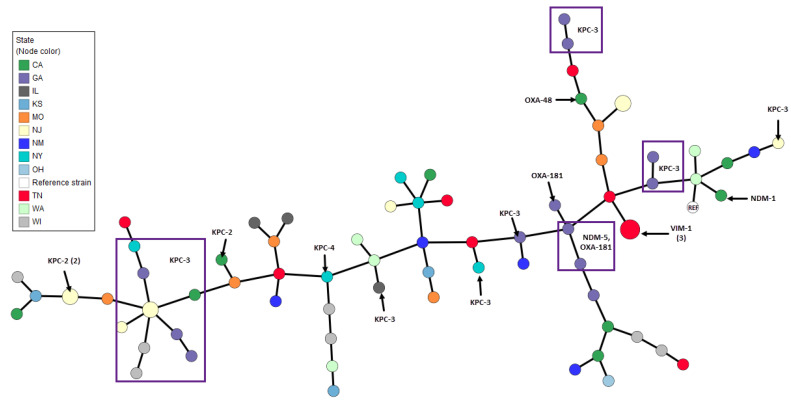
Minimum Spanning Tree based on MLST of *Klebsiella pneumoniae* by U.S. state of isolation and carbapenemase gene identification. Node radius indicates the number of isolates. Squares indicate isolates that harbor the same carbapenemase gene. Reference strain (REF): NZ_CP035929 *Klebsiella pneumoniae* strain B31.

Considerable genetic diversity was observed among the bacterial species using multilocus sequence typing (MLST). Several high-impact lineages were identified in multiple states across the country. For example, among isolates of *Acinetobacter baumannii*, the ST2 clone, which represented 70.6% of all *A. baumannii* in this study, was present in New York, Tennessee, Illinois, Georgia, and California (Supplementary Materials Figure S1). Similarly, among *E. coli* isolates, the ST131 pandemic clone (ST43 by the Pasteur scheme) [12] was the most common strain type observed (37.8%) and was identified in eight U.S. states (Figure 2). Small sporadic clusters of related isolates, especially among the ST131 isolates, were seen in several states (Figure 2). Highly similar clusters of isolates were more evident among *K. pneumoniae*, some of which were indistinguishable when visualized by Minimum Spanning Tree (MST) analysis, including three clusters each containing two closely related strains from New Jersey and a cluster of three strains from Tennessee (Figure 3, larger nodes containing 2 and 3 isolates). Among the isolates from Georgia (purple nodes), a group of four closely related *K. pneumoniae* isolates were identified, with two carrying both *bla*_NDM-5_ and *bla*_OXA-181_, one harboring only *bla*_OXA-181_, and one with no carbapenemase gene. Isolated pairs of more closely related strains were also identified by MST, including one pair of isolates from Missouri, two distinct pairs from Washington (all harboring both *bla*_CTX-M-15_ and *bla*_SHV_), and one pair from Wisconsin (carrying *bla*_KPC-3_) (Figure 3). The most frequent sequence type among *K. pneumoniae* isolates was the emerging ST307 lineage (14.5%), which was present in eight states, followed by ST258 (10.5%), which was found in five states. 

*P. aeruginosa* isolates exhibited considerable strain diversity, which included the pandemic clones ST235 (the most frequent), ST244, ST111, ST274, and ST357. The latter clone, ST357, harbored *bla*_NDM-1_. Additionally, a *P. aeruginosa* ST644 isolate carrying both *bla*_IMP-62_ and *bla*_NDM-1_ and an ST167 isolate that harbored a *bla*_IMP-15_ carbapenemase were identified (Figure S2).

### 2.2. Beta-Lactamase Gene Carriage by Specimen Type 

One or more carbapenemase genes were detected in 20.5% of all isolates, alone or in conjunction with other beta-lactamase genes (Table 1 and Table S2). Among urine cultures, 23.6% of the isolates harbored a carbapenemase gene compared to 16.7% of blood culture isolates (*p* = 0.116) (Table 1). 

**Figure 4:**
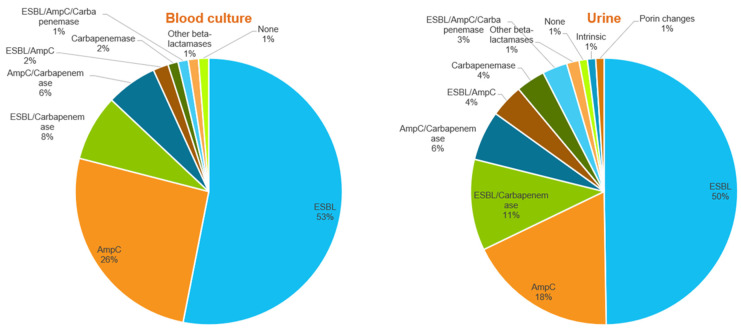
Beta-lactam resistance mechanisms were identified among isolates from blood cultures and urine cultures.

Overall, 66.2% of the isolates harbored ESBL genes, showing an even distribution between the two specimen types, with 67.8% of urine culture isolates carrying an ESBL gene compared to 64.2% of blood culture isolates (*p* = 0.656). AmpC genes were detected in 33.2% of all isolates and were equally distributed among the two sample types (35.8% of blood culture isolates vs. 31.2% of urine isolates; *p* = 0.370). Figure 4 shows the different combinations of beta-lactam resistance mechanisms observed among the blood culture and urine isolates. No significant difference was observed between the two sample types for any combination of beta-lactam resistance mechanisms, including the percentage of AmpC-only genes, which, although higher among blood cultures than among urine isolates, was not statistically significant (25.9% versus 18.1%; *p* = 0.094).

### 2.3. Carbapenemase, ESBL, and AmpC Genes Detected by Whole Genome Sequencing

The predominant carbapenemase genes were *bla*_KPC-2_ and *bla*_KPC-3_, which were harbored by 9.7% of all study isolates and 47.3% of those isolates with carbapenemase genes (Table 2 and Figure S3). The *bla*_NDM_ alleles were present in eight Enterobacterales isolates (two *K. pneumoniae* isolates also carried *bla*_OXA-181_) and two *P. aeruginosa* isolates, one of which also carried *bla*_IMP-62._ The remaining carbapenem-resistant *P. aeruginosa* isolate carried *bla*_IMP-15._ Co-carriage of two *bla*_OXA-type_ carbapenemases was seen in 11 of 16 carbapenem-resistant *A. baumannii* isolates, including the combination of *bla*_OXA-23_ and *bla*_OXA-66_ in five isolates. Other carbapenemase genes in *A. baumannii* included *bla*_OXA-24_, *bla*_OXA-71_, *bla*_OXA-78_, *bla*_OXA-82_, *bla*_OXA-95_, *bla*_OXA-237_, and *bla*_OXA-407._ The remaining carbapenemase genes, including *bla*_VIM_, *bla*_OXA-48_, and *bla*_SME_, were detected sporadically. 

Among ESBL genes, *bla*_CTX-M_ types were the most commonly identified (85.8% of all ESBLs) and included *bla*_CTX-M-15_ (58.2% of ESBL genes), *bla*_CTX-M-27_ (10.5%), and *bla*_CTX-M-14_ (7.5%) (Figure S4). The *bla*_CTX-M_ genes, especially *bla*_CTX-M-15_, were detected with other ESBL genes, such as *bla*_SHV-187_, 24.9% of the time. All isolates harboring *bla*_CTX-M_ genes displayed an ESBL phenotype (Figure S4). Among *E. coli* ST131 isolates in particular, 94.7% harbored an ESBL gene, of which 72.2% were *bla*_CTX-M-15_, 20.4% *bla*_CTX-M-27_, and 7.4% *bla*_CTX-M-14_. Isolates harboring the latter two *bla*_CTX-M_ genes clustered in a separate branch on the *E. coli* Minimum Spanning Tree (MST) (Figure 2, red circle). Similarly, all ST307 *K. pneumoniae* isolates carried the *bla*_CTX-M-15_ genes (Table S2). The *bla*_SHV_ beta-lactamase genes were the next most frequent ESBL family, and they were carried either alone or with other ESBL genes (31.4%). The *bla*_SHV-187_ gene was the most frequent (22.6%), followed by *bla*_SHV-12_ (3.3%). 

A total of 120 AmpC genes were identified among 11 different species. The chromosomal *bla*_PDC_ and *bla*_ADC_ genes, found in *P. aeruginosa* and *A. baumannii*, respectively, together constituted 42.5% of all the AmpC genes detected (Figure S5). Among the acquired AmpC genes, *bla*_ACT_ types were the most common (30.8% of all AmpC genes) and were identified in all *E. cloacae* complex isolates and in half of the *K. aerogenes* isolates. This is followed in frequency by *bla*_CMY_ genes, which were identified in *E. coli*, *C. freundii*, *K. aerogenes*, *P. mirabilis*, and *K. pneumoniae* isolates (Figure S5). 

### 2.4. Comparison of Phenotypic and Genotypic Resistance Profiles

Table 2 and Table 3 illustrate the most frequent carbapenemase genes identified and the correlation between the isolates’ genotype and phenotypic expression of resistance to ertapenem, imipenem, and meropenem for Enterobacterales, and imipenem and meropenem for *A. baumannii* and *P. aeruginosa*, respectively (see Figure S3 for additional information). 

Only 61.3% of organisms harboring a *bla*_SHV_ ESBL expressed the ESBL phenotype by disk diffusion (DD) and 66.7% by broth microdilution (BMD), when compared to 99.2% and 89.3% for isolates carrying *bla*_CTX-M_, respectively (*p* < 0.001 for both). This difference is likely due to the fact that 38.7% of *bla*_SHV_ were present concurrently with a carbapenemase gene, while only 8.3% of *bla*_CTX-M_ were associated with a carbapenemase gene, whose presence tends to confound phenotypic ESBL detection (Table S1). Other ESBL genes, like *bla*_TEM-106_ and *bla*_TEM-168_, *bla*_VEB-1_ and *bla*_PME-1_, and the chromosomal ESBLs *bla*_OXY-1-1_, *bla*_OXY-1-7_, and *bla*_OXY-2-8_, were seen sporadically (Table S2). 

Eight isolates in our study, two from blood cultures and six from urine, concurrently harbored an ESBL, an AmpC, and a carbapenemase gene (Figure 4 and Table S1). These isolates, from California, New Jersey, and Wisconsin, included two *C. freundii*, two *P. aeruginosa* (one ST644 harboring two carbapenemase genes and epidemic clone ST357 carrying *bla*_NDM-1_), an *E. coli*, a *K. pneumoniae* (high-risk clone ST15), an *Enterobacter asburiae*, and an *A. baumannii* (ST235). All of the isolates were phenotypically resistant to the three carbapenems tested, except for the ST-721-like *E. coli* harboring *bla*_OXA-48_, *bla*_CTX-M-27_, and *bla*_DHA-1_, which was susceptible to meropenem and intermediate to imipenem by BMD (Table 2).

Overall, 90.3% of the 196 isolates with ESBL genes but no carbapenemase genes were resistant to cefepime by BMD (Minimum Inhibitory Concentration ≥16 µg/mL). (Table S1). The rate of cefepime resistance remained high (84.9%) even after excluding 70 isolates carrying *bla*_OXA-1_, which has been implicated in cefepime hydrolysis [2]. 

Among the 54 Enterobacterales that harbored a carbapenemase gene, 42 were resistant to ertapenem, imipenem, and meropenem. Five isolates were resistant to ertapenem and imipenem but intermediate or susceptible to meropenem, while an additional six isolates had variable patterns of resistance, intermediate, and susceptible results to the three antimicrobial agents. The final organism, which, was susceptible to all three drugs, harbored a *bla*_KPC-2_ gene, which on further analysis, was truncated and non-functional. The three *P. aeruginosa* isolates with carbapenemase genes were resistant to both imipenem and meropenem, while 15 of the 17 *A. baumannii* isolates were resistant to both antimicrobial agents. One *A. baumannii* isolate was intermediate to imipenem and resistant to meropenem, while the final isolate, which contained *bla*_OXA-78_, was susceptible to both carbapenem agents. Interestingly, all *A. baumannii* isolates were negative by the combined mCIM/eCIM method for detection of carbapenemase activity.

### 2.5. Resistance to Newer Beta-Lactam/Beta-Lactamase Inhibitor Combinations

Overall, 5.2% (16 of 309) Enterobacterales and 15.2% (5 of 33) *P. aeruginosa* isolates were resistant to ceftazidime–avibactam (CZA) by BMD (MIC ≥ 16/4 µg/mL) (Table S1). Of those, 12 Enterobacterales (including 6 *K. pneumoniae*, 3 *E. coli*, one *E. cloacae hormaechei*, one *S. marcescens*, and one *K. michiganensis*) and 3 *P. aeruginosa* contained metallo-beta-lactamases, two in combination with *bla*_OXA-181_. In addition, one *E. hormaechei* carried *bla*_KPC-2_, and five isolates, including *P. aeruginosa*, *K. aerogenes*, *P. rettgeri*, and *S. nematodiphila* (isolates #17,636, 17,708, 17,213, and 17,835 respectively in Table S1), were negative for carbapenemase genes and negative for mCIM and eCIM. Among Enterobacterales, 26.5% were resistant and 3.9% were intermediate to ceftolozane-tazobactam (CT), while resistance to the combination drug among *P. aeruginosa* was 12.1%, with an additional 3.0% of results reported as intermediate. Three of the four CT-resistant *P. aeruginosa* harbored a metallo-beta-lactamase gene, while the fourth carried only *bla*_OXA-395_ and *bla*_PDC-471_ (amino-acid mutations in *bla*_PDC_ at R79Q and T105A have been linked to CT resistance) [13]. Resistance to meropenem-vaborbactam (MEV) among Enterobacterales was 3.9% (12 of 309). Ten of the resistant isolates harbored a metallo-beta-lactamase gene, one had a serine carbapenemase gene (*bla*_OXA-48_), and one *P. rettgeri* isolate did not carry a carbapenemase gene (Tables S1 and S2). 

Among all carbapenemase-producing *E. coli*, two isolates harboring *bla*_NDM-5_, one ST44 isolated from urine and one ST2 from a blood culture (isolates #17184 and 17198, respectively, in Table S2), both from the same institution in California, had the four amino acid YRIK insertion at position 333 in *pbp3*, associated with resistance to aztreonam-avibactam and decreased susceptibility to cefiderocol (phenotypic resistance to cefiderocol and aztreonam-avibactam were not assessed). The two isolates were obtained from different patients who were unlinked epidemiologically.

## 3. Discussion

The goal of this study was to investigate the prevalence of novel beta-lactamase-mediated resistance mechanisms in a convenience sample of cephalosporin- or carbapenem-resistant isolates from blood and urine specimens from patients in 12 geographically dispersed U.S. hospitals. A second goal was to identify potential gaps in susceptibility testing methods that may miss emerging resistance mechanisms. It is important to note that the mCIM and eCIM tests failed to detect carbapenemase production in every one of the carbapenem-resistant *A. baumannii* isolates in this study that carried a carbapenemase gene. Several new protocols with modified mCIM/eCIM conditions that may detect these isolates have been proposed but not yet standardized [14]. 

The correlation of broth microdilution results to disk diffusion results was high in this study, with the exception of the results for cefepime, for which recognition of resistance was statistically lower by the CLSI disk diffusion method when MIC results were interpreted using either CLSI or EUCAST breakpoints [15,16]. The CLSI and EUCAST MIC interpretations were highly correlated. 

Minimum spanning tree analysis of the key bacterial species in this study indicated that, except for a few pairs of *K. pneumoniae* isolates and several *E. coli* ST131 isolates, the majority of the isolates, representing 15 species, did not appear to be associated with hospital outbreaks. Thus, this convenience sample provides us with a broad snapshot of beta-lactamase-mediated resistance among Gram-negative organisms in the United States, including ESBLs, AmpC enzymes, and carbapenemases. Not surprisingly, the *bla*_CTX-M_ genes were the most common ESBLs detected; however, there were a number of SHV variants, including *bla*_SHV-12_, *bla*_SHV-106_ [17], and *bla*_SHV-187_, which were detected, often in combination with *bla*_CTX-M_ genes. Newer *bla*_SHV_ alleles, such as *bla*_SHV-187_, are emerging globally [18]. Plasmid-mediated AmpCs, such as *bla*_ACT_, *bla*_CMY_, and *bla*_DHA_, were rare in the Enterobacterales, while chromosomal AmpC enzymes were found in all *A. baumannii* and *P. aeruginosa* isolates, where they likely mediated resistance to multiple beta-lactam agents. 

The *E. coli* high-risk lineage ST131 was the predominant sequence type observed among the *E. coli* isolates from both blood and urine. ST131 has been described as the “quintessential example of an international multiresistant high-risk clone” [19], and in our convenience sample, both of the C subclades described in the literature, i.e., C1, characterized by carriage of *bla*_CTX-M-14_ and *bla*_CTX-M-27_, and C2, harboring *bla*_CTX-M-15_, were observed [20]. Strains belonging to ST131 were identified in isolates from all 12 participating laboratories, consistent with its broad geographic expansion during the last two decades [20]. Similarly, the two dominant clones among *K. pneumoniae* isolates, i.e., ST307 and ST258, are both high-risk international clones. ST307 isolates carried *bla*_CTX-M-15_ (similar to that of *E. coli* ST131 [20,21]), although only one ST307 was carbapenem-resistant and carried *bla*_KPC-3_. Similarly, most of the *A. baumannii* isolates characterized in our study belonged to the successful international clone ST2 (representative of global clone II) [22]. 

In our study, most ESBL isolates were resistant to cefepime by BMD. There have been mixed reports on the effectiveness of cefepime for the treatment of ESBL infections, ranging from cefepime therapy being comparable to carbapenem therapy to being inferior [23,24,25,26]. Higher cefepime MIC levels have been previously associated with CTX-M enzymes [27], which corroborates what was observed in our study. 

The breadth of carbapenemases identified was surprising. A total of 23 different carbapenemase genes were seen among 13 different species. In addition to the *bla*_KPC_, *bla*_NDM_, *bla*_OXA-48_, and *bla*_VIM_ variants detected among the Enterobacterales isolates, the *A. baumannii* isolates contained a variety of *bla*_OXA*Ab*_ variants, such as *bla*_OXA-23_, *bla*_OXA-24_, *bla*_OXA-51_, and several OXA variants not commonly reported from US isolates. These included the *bla*_OXA-66_ family (sometimes called the *bla*_OXA-51/66_ family) and its variants, *bla*_OXA-71_, *bla*_OXA-78_, and *bla*_OXA-317_. *bla*_OXA-237_ is another carbapenemase found in *Acinetobacter* species; however, one that is derived from the *bla*_OXA-134_ group. The proliferation of carbapenemase genes seen among *Acinetobacter* species in the United States is important for clinical microbiologists to recognize since many laboratories do not test *Acinetobacter* species for carbapenemases, presuming that the carbapenem resistance is efflux-mediated [28]. Furthermore, beta-lactam/beta-lactamase combination drugs with activity against *Acinetobacter* isolates producing class D beta-lactamases are now available [29].

As noted before, the mCIM/eCIM method was not reliable for detecting carbapenemases in *A. baumannii* in our study. Thus, it may be worth considering the use of genotypic methods for detecting and differentiating among serine and metallo-beta-lactamase genes among carbapenem-resistant *Acinetobacter* species to guide therapeutic strategies for serious infections.

There were relatively few *P. aeruginosa* isolates in this collection, and only three (two from Northern California and one from Southern California) contained a carbapenemase gene. Carbapenemase-producing strains of *P. aeruginosa* remain rare in the United States but are increasingly prevalent elsewhere and associated with higher mortality [30]. One isolate carried *bla*_IMP-15_; another, which belonged to the ST357 international lineage, harbored *bla*_NDM-1_; and the third, an ST644, contained both *bla*_IMP-62_ and *bla*_NDM-1._ An extensively drug-resistant *P. aeruginosa* ST644 isolate was recently reported from an infected footpad of a Magellanic penguin in Brazil [31]. Because metallo-carbapenemases are common in *P. aeruginosa* and especially since ceftolozane-tazobactam, which is often used to treat *P. aeruginosa* infections, is not active against metallo-enzymes, consideration should be given to testing isolates of *P. aeruginosa*, especially from positive blood cultures, for carbapenemase production [32]. As opposed to the problems noted for testing *A. baumannii* by mCIM and eCIM, all three *P. aeruginosa* isolates were positive for carbapenemase production with the mCIM test; however, one of the three isolates, the one that carried both *bla*_IMP-62_ and *bla*_NDM-1_, was incorrectly identified as a serine carbapenemase producer, likely because IMP enzymes are more resilient to zinc chelation and may cause a false-positive eCIM result unless EDTA concentration is increased [33].

All of the “big 5” carbapenemases are circulating in the United States [1]. The presence of a metallo-beta-lactamase (such as *bla*_IMP_, *bla*_NDM_, or *bla*_VIM_) in a bacterial isolate can compromise the use of novel beta-lactam/beta-lactamase inhibitor combinations, such as ceftazidime-avibactam, meropenem-vaborbactam, and imipenem-relebactam, as these antimicrobial agents often are not effective in the presence of these enzymes [34]. It is also important to note that carbapenems may be ineffective for the treatment of carbapenemase-producing organisms even when the MIC is in the susceptible range [35,36]. One *bla*_NDM-7_ gene, which encodes an NDM variant with increased carbapenem-hydrolyzing activity [37], was observed in a *K. michiganensis* isolate from the urine of a patient in California. The *bla*_NDM-7_ gene was located on an IncX3-type plasmid, which is an important mechanism of resistance gene dissemination for *bla*_NDM-5_ determinants [38].

Among the Enterobacterales, *bla*_KPC_, *bla*_NDM_, *bla*_OXA-48_, and *bla*_VIM_ carbapenemases were detected, while *bla*_IMP_ was observed in two *P. aeruginosa* isolates. The isolates carrying both metallo-beta-lactamases and ESBL genes were resistant to the entire panel of beta-lactam and beta-lactam–beta-lactamase inhibitor combinations tested, showing how this combination of resistance genes can limit therapeutic options. Two isolates of *E. coli*, both harboring *bla*_NDM-5_ and *bla*_CTX-M-15_, carried the *pbp3* insertions that have been linked to decreased susceptibility to cefiderocol and to the beta-lactam–beta-lactamase inhibitor aztreonam-avibactam. The latter combination has shown promising results for the treatment of Enterobacterales co-harboring metallo-beta-lactamases and ESBLs or AmpC genes [39,40]. There have been increasing reports of *bla*_KPC_ variants (including 77 out of 156 known *bla*_KPC_ alleles according to the NCBI Reference Gene Catalog [41]) that can confer resistance to ceftazidime-avibactam [42]. We only identified one *bla*_KPC-2_-containing isolate (an *E. hormaechei*) with resistance to CZA. This organism also carried a *bla*_ACT-27_-like ampC gene in addition to the carbapenemase gene. CZA resistance can be associated with mutations or with increased expression of *ampC* genes [43].

This study has several limitations, including the small number of participating laboratories and the limited number of isolates collected. Furthermore, the panel of beta-lactam and beta-lactam–beta-lactamase inhibitor combinations available for testing did not include imipenem-relebactam. However, the geographic diversity of the laboratories and the broad spectrum of beta-lactamases ultimately observed among the isolates indicate that the primary goal of our study, i.e., to obtain a snapshot of currently circulating beta-lactamase genes in the United States, was successful. 

In summary, these data indicate that mechanisms of beta-lactamase-mediated resistance in bacterial isolates obtained from patients across the United States continue to evolve, with new mechanisms of carbapenem resistance emerging, especially in *A. baumannii*. Phenotypic detection of carbapenem resistance in this species proved difficult with the mCIM and eCIM methods. Clearly, carbapenemase genes are much more diverse than just *bla*_KPC_, and the number of metallo-carbapenemases, including *bla*_IMP_, *bla*_NDM_, and *bla*_VIM_, circulating could prove a challenge for the selection of effective antimicrobial therapy. This study also shows the importance of internationally recognized epidemic clones in spreading antimicrobial resistance. 

## 4. Materials and Methods

### 4.1. Isolate Selection Criteria

Twelve geographically dispersed hospitals or hospital system laboratories from across the United States participated in this study. Each laboratory was invited to send 15 Gram-negative isolates from bloodstream infections and 15 Gram-negative organisms isolated from urinary tract infections, each from a unique patient, during 2021 and 2022. Isolates had to meet any of the following criteria: Enterobacterales that were non-susceptible to cefotaxime, ceftriaxone, ceftazidime, ertapenem, meropenem, or imipenem; *Acinetobacter* or *Pseudomonas* species that were non-susceptible to meropenem or imipenem; or any Gram-negative bacterial isolate that was positive by the modified carbapenem inactivation method (mCIM) or was confirmed to be a carbapenemase producer by nucleic acid amplification testing or other laboratory method.

### 4.2. Bacterial Identification and Antimicrobial Susceptibility Testing (AST)

Identification of the Gram-negative bacterial isolates was performed by MALDI-TOF MS (Bruker Daltonics GmbH, Bremen, Germany) according to the manufacturer’s instructions. Antimicrobial susceptibility testing was conducted using the Neg MIC 56 panel on the MicroScan WalkAway 40 Plus system (Beckman Coulter, Inc., West Sacramento, CA, USA) as described by the manufacturer. Minimal inhibitory concentration (MIC) results were interpreted according to Clinical and Laboratory Standards Institute (CLSI) guidelines [15]. Quality control organisms included *P. aeruginosa* ATCC 27853, *Escherichia coli* ATCC 25922, and ATCC 35218, *Klebsiella pneumoniae* ATCC 700,603, and *K. pneumoniae* ATCC BAA-1705. A meropenem disk was added to agar plates in each subculture to maintain antimicrobial pressure and prevent loss of beta-lactam resistance determinants. Isolates were also tested for susceptibility to ertapenem, imipenem, meropenem, cefotaxime (with and without clavulanic acid), ceftazidime (with and without clavulanic acid), ceftriaxone, cefepime, aztreonam, and ceftazidime/avibactam by the disk diffusion method on Mueller-Hinton agar (Hardy Diagnostics, Santa Maria, CA, USA), as described by CLSI [44]. ESBL production was tested with the disk diffusion method using both cefotaxime (CTX) (30 mg) and ceftazidime (CAZ) (30 mg) disks alone and in combination with clavulanic acid (CA) (10 mg) (Becton, Dickinson, Sparks, MD, USA) as described by CLSI (CLSI M100). Carbapenemase production was detected with the modified carbapenem inactivation method (mCIM), which was employed in conjunction with the EDTA-modified carbapenem inactivation method (eCIM) to differentiate serine carbapenemases from metallo-carbapenemases, according to CLSI guidelines, with the following two exceptions: eCIM testing was extended to all isolates instead of only Enterobacterales, and mCIM/eCIM testing was also performed on *Acinetobacter* species [15].

### 4.3. Whole Genome Sequencing

Pure cultures of the organisms were grown overnight on blood agar plates with a meropenem disk (Hardy Diagnostics) added to the second streak area. Nucleic acids were extracted with the Qiagen DNeasy Blood and tissue kit using the Qiacube instrument (Qiagen, Valencia, Santa Clarita, CA, USA). Genomic libraries were prepared with the Illumina DNA Prep Kit (Illumina, San Diego, CA, USA) and sequenced on the Illumina MiSeq using Reagent Kit v2 chemistry (Illumina). De novo assemblies, multi-locus sequence typing (MLST), k-mer based prediction of species, construction of minimum spanning trees, and detection of acquired antimicrobial resistance genes and point mutations were performed with the CLC Genomics Workbench version 22.0.2 and CLC Microbial Genomics Module version 22.1.1 (QIAGEN Bioinformatics, Aarhus, Denmark). All procedures were performed in accordance with the manufacturers’ instructions. All nucleic acid sequence data obtained in this study have been deposited in the NCBI BioProject database at https://www.ncbi.nlm.nih.gov/bioproject/ (accessed on 8 June 2023) with links to BioProject Accession # PRJNA981469.

### 4.4. PBP3 Sequence Analysis

Polymorphisms in the penicillin-binding protein 3 (PBP3, encoded by the gene *ftsI*), specifically insertions of amino acids YRIN or YRIK at position 333, which have been associated with decreased susceptibility to aztreonam-avibactam as well as cefiderocol in *E. coli*, were determined as previously described [45,46,47].

## Figures and Tables

**Table 1:** Number and percentage of beta-lactamase genes identified in the two sample types.

	Blood Culture (N = 162)		Urine (N = 199)			All Isolates (N = 361)	
	No. of Isolates	%	No. of Isolates	%	*p*-Value	No. of Isolates	%
**Carbapenemase genes**	27	16.7%	47	23.6%	0.116	74	20.5%
**ESBL genes**	104	64.2%	135	67.8%	0.503	239	66.2%
**AmpC genes**	58	35.8%	62	31.2%	0.370	120	33.2%
**Other beta-lactamase genes detected ^1^**	2	1.2%	3	1.5%	1.000	5	1.4%
**No beta-lactamase genes detected**	2	1.2%	6	3.0%	0.304	8	2.2%

^1^ One *bla*_FONA-6_ (*Serratia fonticola*), 3 *bla*_TEM-1B_ (*Escherichia coli*) and 1 *bla*_TEM-1A_ (*Serratia nematodiphila*).

**Table 2:** Beta-lactamase gene carriage and phenotypic resistance profiles of carbapenemase-producing Enterobacterales isolates.

Sample Type	Organism by K-mer Spectra	MLST ^1^	State	Carbapenemase Gene	AmpC Gene	ESBL Gene	ETP	IPM	MEM	Other Resistance Phenotypes	mCIM/eCIM Report
BC	*Citrobacter freundii*	ST98	TN	KPC-2	CMY-109	None	R	R	S	ATM, CTX, CAZ, TZP	serine carbapenemase detected
UC	*Citrobacter freundii*	ST415	WI	KPC-2	CMY-48	SHV-12	R	R	R	ATM, CTX, CAZ, CT, TZP	serine carbapenemase detected
UC	*Citrobacter freundii*	ST344-like	WI	KPC-2	CMY-108	SHV-12	R	R	R	ATM, FEP, CTX, CAZ, CT, TZP	serine carbapenemase detected
UC	*Enterobacter asburiae*	ST252	CA	KPC-2	ACT-3	SHV-12	R	R	R	ATM, FEP, CTX, CAZ, CT, TZP	serine carbapenemase detected
UC	*Enterobacter cloacae*	ST171	NJ	KPC-3	ACT-16	None	R	R	R	ATM, FEP, CTX, CAZ, CT, TZP	serine carbapenemase detected
UC	*Enterobacter hormaechei*	ST110	CA	KPC-2	ACT-15	None	R	R	R	ATM, CTX, CAZ, CZA, CT, TZP	serine carbapenemase detected
UC	*Enterobacter hormaechei*	ST1377	NM	NDM-1	ACT-7	None	R	R	R	FEP, CTX, CAZ, CZA, CT, MEV, TZP	metallo beta-lactamase detected
UC	*Escherichia coli*	ST721-like	CA	OXA-48	DHA-1	CTX-M-27	R	I	S	ATM, FEP, CTX, CAZ, ETP	serine carbapenemase detected
UC	*Escherichia coli*	ST691-like	CA	KPC-2	None	None	R	R	S	ATM, CTX, TZP	serine carbapenemase detected
UC	*Escherichia coli*	ST44	CA	NDM-5	None	CTX-M-15	R	R	R	ATM, FEP, CTX, CAZ, CZA, CT, MEV, TZP	metallo beta-lactamase detected
BC	*Escherichia coli*	ST2	CA	NDM-5	None	CTX-M-15	R	R	R	ATM, FEP, CTX, CAZ, CZA, CT, MEV, TZP	metallo beta-lactamase detected
BC	*Escherichia coli*	ST221	CA	OXA-48	None	None	S	I	S	TZP	serine carbapenemase detected
UC	*Escherichia coli*	ST721-like	CA	KPC-2 (truncated)	None	CTX-M-15	S	S	S	ATM, FEP, CTX, CAZ	carbapenemase not detected
UC	*Escherichia coli*	ST43/ST131 ^2^	GA	KPC-3	None	None	S	I	S	ATM, CTX, CAZ, TZP	serine carbapenemase detected
UC	*Escherichia coli*	ST33	GA	NDM-1	None	None	R	R	R	FEP, CTX, CAZ, CZA, CT, TZP	metallo beta-lactamase detected
UC	*Klebsiella aerogenes*	ST3-like	GA	KPC-3	None	None	R	R	R	ATM, FEP, CTX, CAZ, CT, TZP	serine carbapenemase detected
UC	*Klebsiella aerogenes*	ST3-like	GA	KPC-3	None	None	R	R	R	ATM, FEP, CTX, CAZ, CT, TZP	serine carbapenemase detected
UC	*Klebsiella michiganensis*	ST59-like	CA	NDM-7	None	OXY-2-8	R	R	R	ATM, FEP, CTX, CAZ, CZA, CT, MEV, TZP	metallo beta-lactamase detected
BC	*Klebsiella michiganensis*	ST85	WI	KPC-2	None	OXY-1-7	R	R	R	ATM, CTX, TZP	serine carbapenemase detected
BC	*Klebsiella michiganensis*	ST311	GA	KPC-3	None	OXY-4-1, SHV-187	R	R	R	ATM, FEP, CTX, CAZ, CT, TZP	serine carbapenemase detected
UC	*Klebsiella oxytoca*	ST180	GA	KPC-3	None	OXY-1-4	S	R	S	ATM, TZP	serine carbapenemase detected
UC	*Klebsiella pneumoniae*	ST15	CA	NDM-1	CMY-6	CTX-M, SHV-106	R	R	R	ATM, FEP, CTX, CAZ, CZA, CT, MEV, TZP	metallo beta-lactamase detected
UC	*Klebsiella pneumoniae*	ST101	CA	OXA-48	None	CTX-M-15	R	R	R	ATM, FEP, CTX, CAZ, CT, MEV, TZP	serine carbapenemase detected
BC	*Klebsiella pneumoniae*	ST11	CA	KPC-2	None	CTX-M-65, SHV-187	R	R	R	ATM, FEP, CTX, CAZ, CT, TZP	serine carbapenemase detected
UC	*Klebsiella pneumoniae*	ST258	NJ	KPC-3	None	SHV-12	R	R	R	ATM, FEP, CTX, CAZ, CT, TZP	serine carbapenemase detected
UC	*Klebsiella pneumoniae*	ST412	NJ	KPC-2	None	SHV-187	R	R	R	ATM, CTX, TZP	serine carbapenemase detected
UC	*Klebsiella pneumoniae*	ST412	NJ	KPC-2	None	SHV-187	R	R	R	ATM, CTX, TZP	serine carbapenemase detected
UC	*Klebsiella pneumoniae*	ST258	NJ	KPC-3	None	SHV-12	R	R	R	ATM, FEP, CTX, CAZ, CT, TZP	serine carbapenemase detected
UC	*Klebsiella pneumoniae*	ST258	NJ	KPC-3	None	None	R	R	R	ATM, FEP, CTX, CAZ, CT, TZP	serine carbapenemase detected
BC	*Klebsiella pneumoniae*	ST1683-like	NJ	KPC-3	None	SHV-187	R	R	R	ATM, FEP, CTX, CAZ, CT, TZP	serine carbapenemase detected
UC	*Klebsiella pneumoniae*	ST379	GA	KPC-3	None	SHV-187, TEM-168 (trunc)	R	R	R	ATM, FEP, CTX, CAZ, CT, TZP	serine carbapenemase detected
UC	*Klebsiella pneumoniae*	ST469	GA	KPC-3	None	SHV-187	R	R	R	ATM, FEP, CTX, CAZ, CT, TZP	serine carbapenemase detected
UC	*Klebsiella pneumoniae*	ST16	GA	NDM-5, OXA-181	None	SHV-187	R	R	R	ATM, FEP, CTX, CAZ, CZA, CT, MEV, TZP	metallo beta-lactamase detected
UC	*Klebsiella pneumoniae*	ST219	GA	KPC-3	None	CTX-M-15, SHV-187	R	R	S	ATM, FEP, CTX, CAZ, CT, TZP	serine carbapenemase detected
BC	*Klebsiella pneumoniae*	ST13	GA	KPC-3	None	SHV-197	R	R	R	ATM, CTX, CAZ, CT, TZP	serine carbapenemase detected
UC	*Klebsiella pneumoniae*	ST219	GA	KPC-3	None	CTX-M-15, SHV-187	R	R	S	ATM, FEP, CTX, CAZ, CT, TZP	serine carbapenemase detected
BC	*Klebsiella pneumoniae*	ST16	GA	NDM-5, OXA-181	None	CTX-M-15, SHV-187	R	R	R	ATM, FEP, CTX, CAZ, CZA, CT, MEV, TZP	metallo beta-lactamase detected
UC	*Klebsiella pneumoniae*	ST258	NY	KPC-3	None	SHV-187	R	R	R	ATM, FEP, CTX, CAZ, CT, TZP	serine carbapenemase detected
UC	*Klebsiella pneumoniae*	ST348	NY	KPC-4	None	SHV-187	R	I	S	ATM, FEP, CTX, CAZ, CT, TZP	serine carbapenemase detected
UC	*Klebsiella pneumoniae*	ST307	NY	KPC-3	None	CTX-M-15	R	R	R	ATM, FEP, CTX, CAZ, CT, TZP	serine carbapenemase detected
UC	*Klebsiella pneumoniae*	ST258	CA	KPC-3	None	SHV-187, TEM-168 (trunc)	R	R	R	ATM, FEP, CTX, CAZ, CT, TZP	serine carbapenemase detected
UC	*Klebsiella pneumoniae*	ST405	TN	VIM-1	None	CTX-M-15	R	R	R	ATM, FEP, CTX, CAZ, CZA, CT, MEV, TZP	metallo beta-lactamase detected
BC	*Klebsiella pneumoniae*	ST405	TN	VIM-1	None	CTX-M-15, SHV-187	R	R	R	ATM, FEP, CTX, CAZ, CZA, CT, MEV, TZP	metallo-beta lactamase detected
BC	*Klebsiella pneumoniae*	ST405	TN	VIM-1	None	CTX-M-15	R	R	R	ATM, FEP, CTX, CAZ, CZA, CT, MEV, TZP	metallo beta lactamase detected
BC	*Klebsiella pneumoniae*	ST152	IL	KPC-3	None	CTX-M-15	R	R	R	ATM, FEP, CTX, CAZ, CT, TZP	serine carbapenemase detected
UC	*Klebsiella pneumoniae*	ST258	WI	KPC-3	None	SHV-187	R	R	R	ATM, FEP, CTX, CAZ, CT, TZP	serine carbapenemase detected
BC	*Klebsiella pneumoniae*	ST258	WI	KPC-3	None	SHV-187	R	R	R	ATM, FEP, CTX, CAZ, CT, TZP	serine carbapenemase detected
UC	*Klebsiella pneumoniae*	ST258	GA	KPC-3	None	SHV-12	R	R	R	ATM, FEP, CTX, CAZ, CT, TZP	serine carbapenemase detected
BC	*Klebsiella pneumoniae*	ST13	GA	KPC-3	None	CTX-M-15, SHV-187	R	R	R	ATM, FEP, CTX, CAZ, CT, TZP	serine carbapenemase detected
UC	*Klebsiella pneumoniae*	ST16	GA	OXA-181	None	CTX-M-15, SHV-187	R	R	R	ATM, FEP, CTX, CAZ, CT, TZP	serine carbapenemase detected
BC	*Klebsiella pneumoniae*	ST1199	GA	KPC-3	None	SHV-187	R	R	R	ATM, FEP, CTX, CAZ, CT, TZP	serine carbapenemase detected
UC	*Raoultella ornithinolytica*	N/A	NM	OXA-181	None	None	R	R	I	CTX, TZP	serine carbapenemase detected
BC	*Serratia marcescens*	N/A	CA	SME-2	SRT-1	None	R	N/R	R	ATM, CTX, CAZ, MEM	serine carbapenemase detected
BC	*Serratia marcescens*	N/A	TN	VIM-1	SRT-1	None	I	R	I	FEP, CTX, CAZ, CZA, CT, TZP	metallo beta lactamase detected

^1^ Multi-locus sequencing typing was carried out using the 7-locus scheme present in CLC Genomic Workbench (CLC Type with MLST scheme 1.3). When multiple MLST schemes exist, such as for *Acinetobacter baumannii* and *Escherichia coli*, the Pasteur scheme is used. When all alleles cannot be identified, the closest sequence type is listed, followed by “-like”. N/A = no scheme available for that organism. ^2^ ST43 Pasteur/ST131 Achtman scheme ATM: aztreonam; FEP: cefepime; CTX: cefotaxime; CAZ: ceftazidime; CZA: ceftazidime/avibactam; CT: ceftolozane/tazobactam; ETP: ertapenem; IPM: imipenem; MEM: meropenem; MEV: meropenem/vaborbactam; TZP: piperacillin/tazobactam. The antimicrobial susceptibility testing results listed in this table were obtained using the broth microdilution method.

**Table 3:** Beta-lactamase gene carriage and phenotypic resistance profiles of carbapenemase-producing *Acinetobacter baumannii* and *Pseudomonas aeruginosa* isolates.

Sample Type	Organism by K-mer Spectra	MLST ^1^	State	Carbapenemase Gene	AmpC Gene	ESBL Gene	Imipenem Interpretation (BMD)	Meropenem Interpretation (BMD)	Other Resistance Phenotypes	mCIM/eCIM Report
UC	*Acinetobacter baumannii*	ST2	CA	OXA-23, OXA-66	ADC-25	None	R	R	FEP, CTX, CAZ, TZP	carbapenemase not detected
BC	*Acinetobacter baumannii*	ST2	CA	OXA-237, OXA-66	ADC-25	None	I	R	CTX, CAZ, TZP	carbapenemase not detected
BC	*Acinetobacter baumannii*	ST2	CA	OXA-237, OXA-66	ADC-25	None	R	R	CTX, CAZ, TZP	carbapenemase not detected
BC	*Acinetobacter baumannii*	ST235	NJ	OXA-71	ADC-25	SHV-12	R	R	FEP, CTX, CAZ, TZP	carbapenemase not detected
UC	*Acinetobacter baumannii*	ST2	GA	OXA-23	ADC-25	None	R	R	FEP, TZP	carbapenemase not detected
UC	*Acinetobacter baumannii*	ST2	GA	OXA-23, OXA-66	ADC-25	None	R	R	FEP, CTX, CAZ, TZP	carbapenemase not detected
UC	*Acinetobacter baumannii*	ST2	GA	OXA-80	ADC-25	None	R	R	FEP, CTX, CAZ, TZP	carbapenemase not detected
BC	*Acinetobacter baumannii*	ST2	GA	OXA-23, OXA-66	ADC-25	None	R	R	FEP, CTX, CAZ, TZP	carbapenemase not detected
UC	*Acinetobacter baumannii*	ST499	NY	OXA-23, OXA-95	ADC-25	None	R	R	None	carbapenemase not detected
UC	*Acinetobacter baumannii*	ST2	NY	OXA-23, OXA-82	ADC-25	None	R	R	FEP, CTX, CAZ, TZP	carbapenemase not detected
UC	*Acinetobacter baumannii*	ST2	NY	OXA-407	ADC-25	None	R	R	FEP, CTX, CAZ, TZP	carbapenemase not detected
UC	*Acinetobacter baumannii*	ST2	NY	OXA-23, OXA-82	ADC-25	None	R	R	FEP, CTX, CAZ, TZP	carbapenemase not detected
BC	*Acinetobacter baumannii*	ST203	NM	OXA-78	ADC-25	None	S	S	CTX (I)	carbapenemase not detected
UC	*Acinetobacter baumannii*	ST258	CA	OXA-23, OXA-66	ADC-25	None	R	R	FEP, CTX, CAZ, TZP	carbapenemase not detected
BC	*Acinetobacter baumannii*	ST2	TN	OXA-23, OXA-66	ADC-25	None	R	R	FEP, CTX, CAZ, TZP	carbapenemase not detected
BC	*Acinetobacter baumannii*	ST1525-like	TN	OXA-24, OXA-317	ADC-25	None	R	R	TZP	carbapenemase not detected
BC	*Acinetobacter baumannii*	ST2	IL	OXA-24, OXA-66	ADC-25	None	R	R	CTX, CAZ, TZP	carbapenemase not detected
UC	*Pseudomonas aeruginosa*	ST644	CA	IMP-62, NDM-1	PDC-430	PME-1	R	R	ATM, FEP, CAZ, CZA, CT, TZP	serine carbapenemase detected
BC	*Pseudomonas aeruginosa*	ST357	CA	NDM-1	PDC-11	VEB-1	R	R	ATM, FEP, CAZ, CZA, CT, TZP	metallo beta-lactamase detected
BC	*Pseudomonas aeruginosa*	ST167	CA	IMP-15	PDC-445	None	R	R	FEP, CAZ, CZA, CT, TZP	metallo beta lactamase detected

^1^ Multi-locus sequencing typing was carried out using the 7-locus scheme present in CLC Genomic Workbench (CLC Type with MLST scheme 1.3). When multiple MLST schemes exist, such as for *Acinetobacter baumannii* and *Escherichia coli*, the Pasteur scheme is used. When all alleles cannot be identified, the closest sequence type is listed, followed by “-like”. N/A = no scheme available for that organism. ATM: aztreonam; FEP: cefepime; CTX: cefotaxime; CAZ: ceftazidime; CZA: ceftazidime/avibactam; CT: ceftolozane/tazobactam; ETP: ertapenem; IPM: imipenem; MEM: meropenem; MEV: meropenem/vaborbactam; TZP: piperacillin/tazobactam.

## Data Availability

The data presented in this study are available in this article and in the NCBI BioProject database https://www.ncbi.nlm.nih.gov/bioproject/ (accessed on 8 June 2023) with links to BioProject Accession # PRJNA981469.

## Supplementary Materials

The following supporting information can be downloaded at: https://www.mdpi.com/article/10.3390/antibiotics12091386/s1, Figure S1: Minimum Spanning Tree of *Acinetobacter baumannii* color-coded by sequence type; Figure S2: Minimum Spanning Tree of *Pseudomonas aeruginosa* by sequence type; Figure S3: Number of isolates with carbapenemase genes identified and the non-susceptibility of the isolates to carbapenems (ertapenem, imipenem, and meropenem) by BMD; Figure S4: Most frequent ESBL genes identified and their phenotypic identification by BMD and DD; Figure S5: Most frequent AmpC genes identified and their phenotypic resistance to cefepime by BMD and DD (grouped by AmpC gene family); Table S1: Summary of phenotypic results result; Table S2: Summary of genotypic results.
