# Supplementary material for: Characterization of Carbapenemase- and ESBL-Producing Gram-Negative Bacilli Isolated from Patients with Urinary Tract and Bloodstream Infections
Source: Antibiotics (Basel). 2023 Aug 30;12(9):1386. doi: 10.3390/antibiotics12091386 (PMC10525328; doi:10.3390/antibiotics12091386)
Supplement: Supplementary file 1 [file antibiotics-12-01386-s001.zip › Figure S1.pdf]

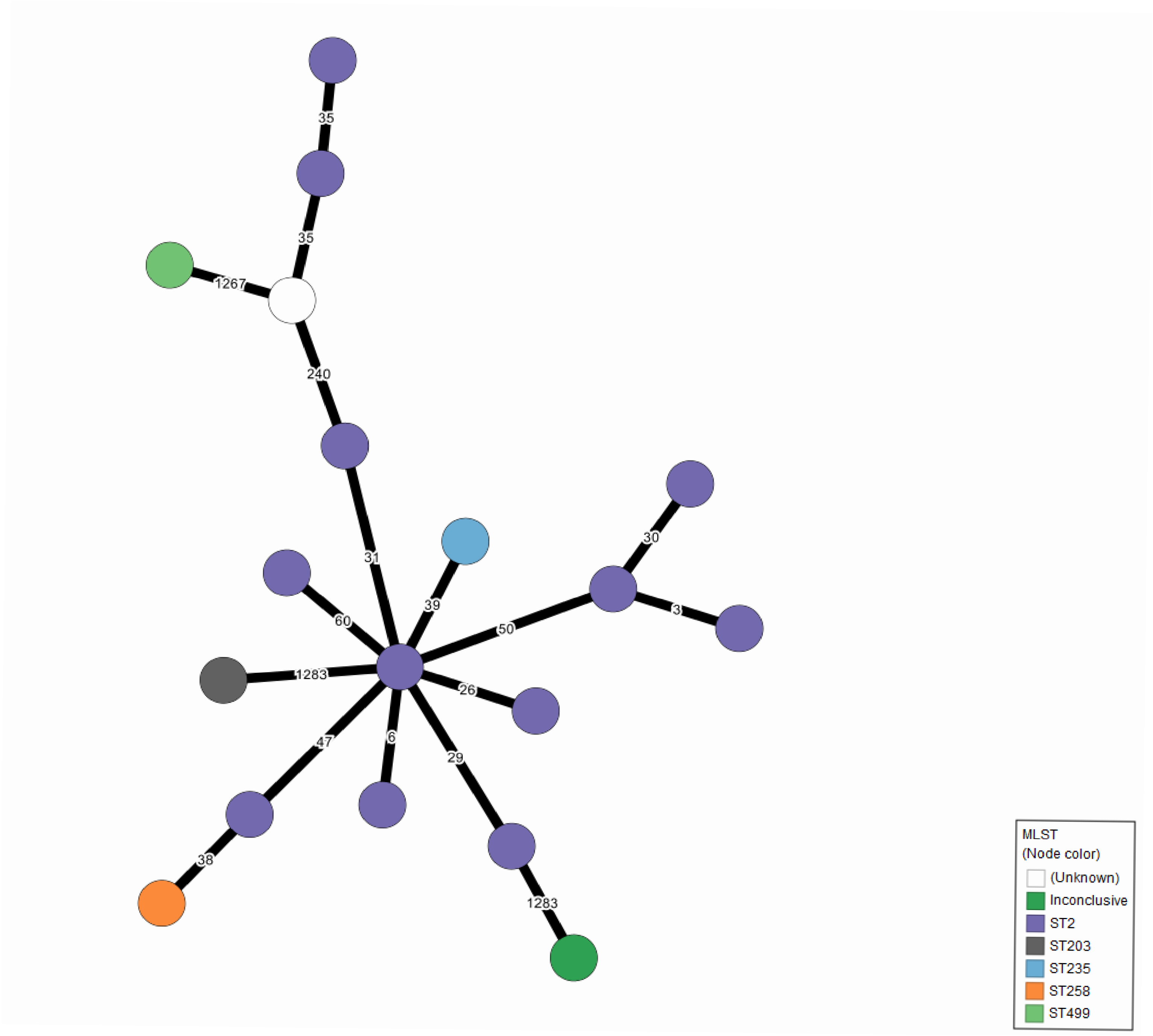

Figure S1. Minimum Spanning Tree of *Acinetobacter baumannii* color-coded by sequence type. Reference strain: NZ_CP042841 *Acinetobacter baumannii* strain BAA-1790.
