# Supplementary material for: Characterization of Carbapenemase- and ESBL-Producing Gram-Negative Bacilli Isolated from Patients with Urinary Tract and Bloodstream Infections
Source: Antibiotics (Basel). 2023 Aug 30;12(9):1386. doi: 10.3390/antibiotics12091386 (PMC10525328; doi:10.3390/antibiotics12091386)
Supplement: Supplementary file 1 [file antibiotics-12-01386-s001.zip › Figure S2.pdf]

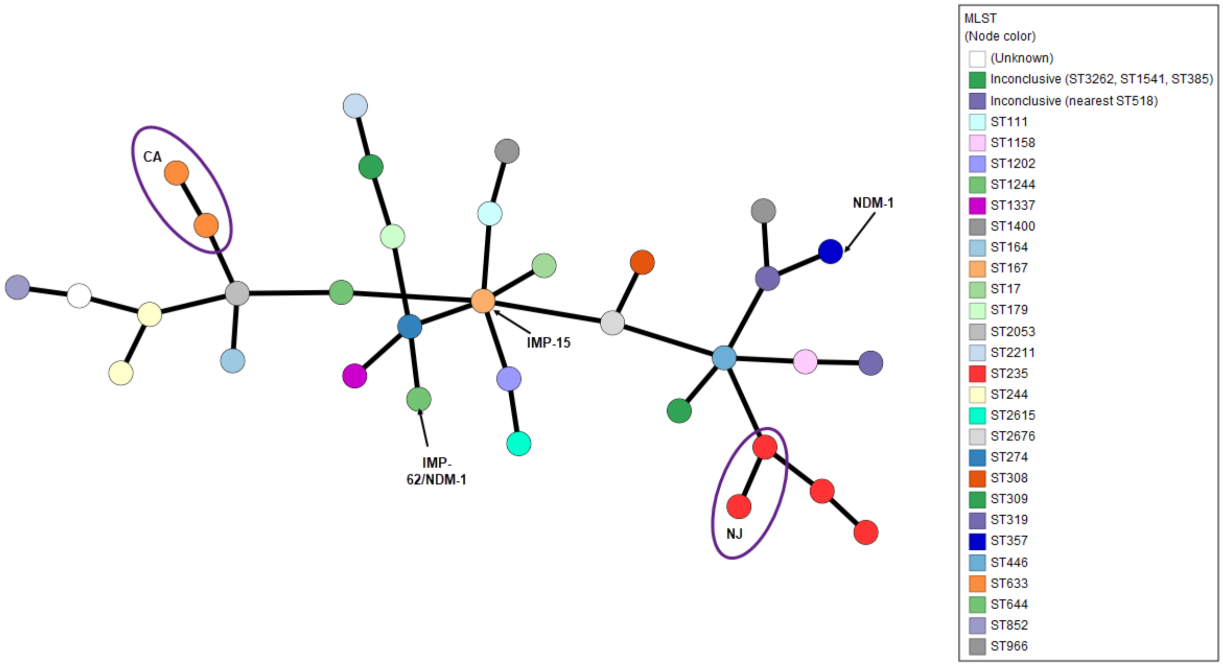

Figure S2. Minimum Spanning Tree of *Pseudomonas aeruginosa* color-coded by sequence type. Circles show clusters of the same sequence type found in the same U.S. state, arrows indicate carbapenemase gene identified. Reference strain: NC_002516 *Pseudomonas aeruginosa* strain PAO1
