# Supplementary material for: Characterization of Carbapenemase- and ESBL-Producing Gram-Negative Bacilli Isolated from Patients with Urinary Tract and Bloodstream Infections
Source: Antibiotics (Basel). 2023 Aug 30;12(9):1386. doi: 10.3390/antibiotics12091386 (PMC10525328; doi:10.3390/antibiotics12091386)
Supplement: Supplementary file 1 [file antibiotics-12-01386-s001.zip › Figure S3.pdf]

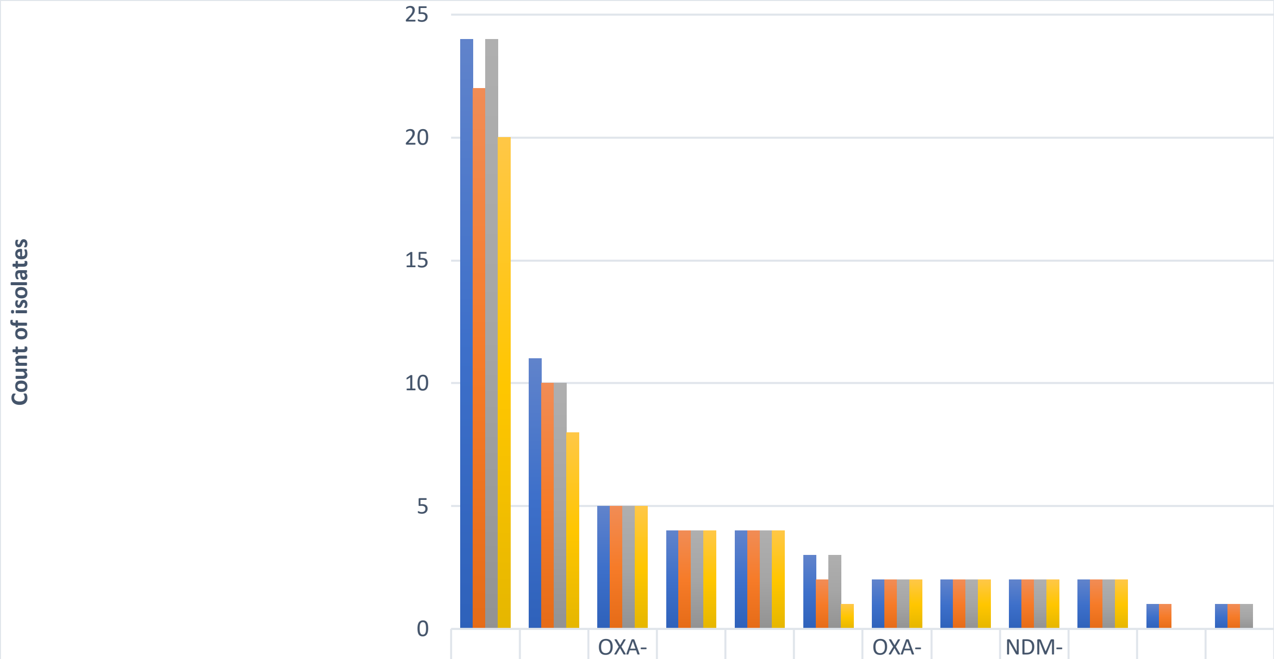

	KPC-3	KPC-2	OXA-23, OXA-66	NDM-1	VIM-1	OXA-48	OXA-237, OXA-66	OXA-181	NDM-5, OXA-181	NDM-5	OXA-78	KPC-4
Isolates with gene or gene combination	24	11	5	4	4	3	2	2	2	2	1	1
Ertapenem NS	22	10	5	4	4	2	2	2	2	2	1	1
Imipenem NS	24	10	5	4	4	3	2	2	2	2	0	1
Meropenem NS	20	8	5	4	4	1	2	2	2	2	0	0

Figure S3. Number of isolates with carbapenemase genes identified and the non-susceptibility of the isolates to carbapenems (ertapenem, imipenem and meropenem) by BMD. Ertapenem results for *P. aeruginosa* and *A. baumannii* were reported as NS due to intrinsic resistance. (N=74; showing 12 most frequent and any genes that were susceptible to meropenem). NS= I +R
