# Supplementary material for: Characterization of Carbapenemase- and ESBL-Producing Gram-Negative Bacilli Isolated from Patients with Urinary Tract and Bloodstream Infections
Source: Antibiotics (Basel). 2023 Aug 30;12(9):1386. doi: 10.3390/antibiotics12091386 (PMC10525328; doi:10.3390/antibiotics12091386)
Supplement: Supplementary file 1 [file antibiotics-12-01386-s001.zip › Figure S5.pdf]

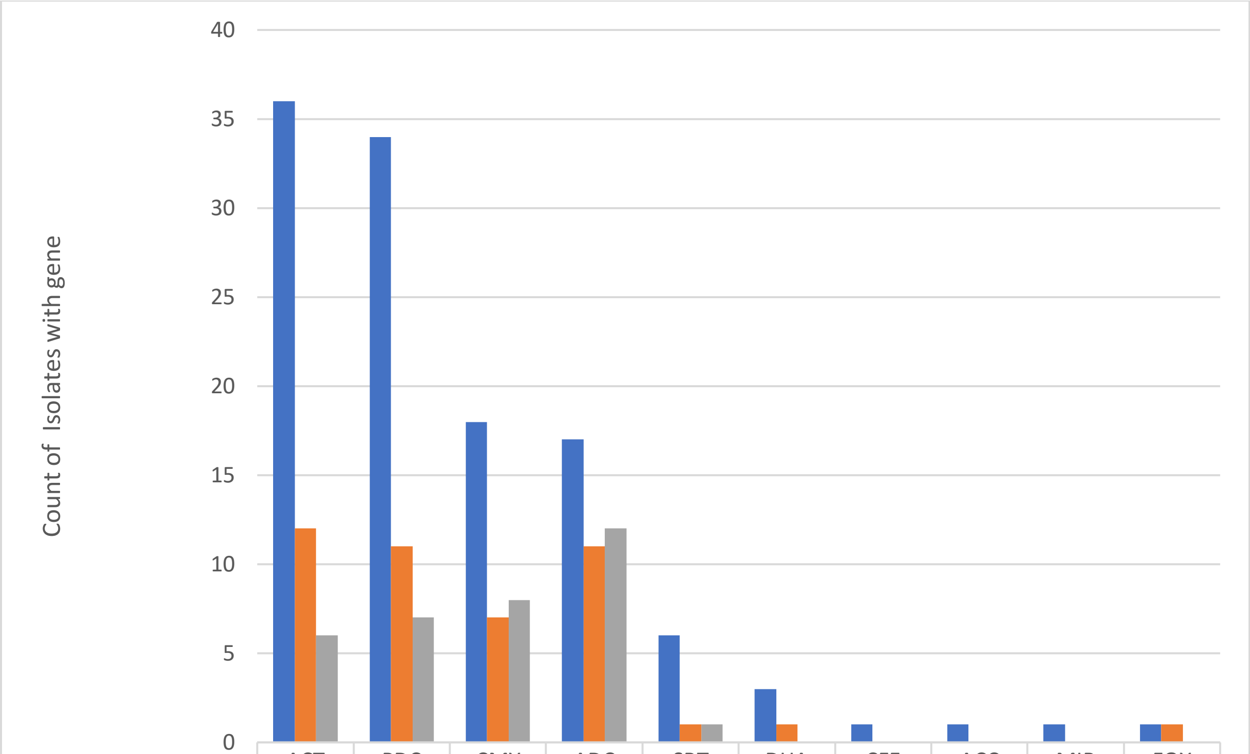

	ACT	PDC	CMY	ADC	SRT	DHA	CFE	ACC	MIR	FOX
Isolates with gene	36	34	18	17	6	3	1	1	1	1
FEP R (BMD)	12	11	7	11	1	1	0	0	0	1
FEP R (DD)	6	7	8	12	1	0	0	0	0	0

Figure S5. Most frequent AmpC genes identified and their phenotypic resistance to cefepime by BMD and DD (grouped by AmpC gene family).
