# Supplementary material for: Characterization of Carbapenemase- and ESBL-Producing Gram-Negative Bacilli Isolated from Patients with Urinary Tract and Bloodstream Infections
Source: Antibiotics (Basel). 2023 Aug 30;12(9):1386. doi: 10.3390/antibiotics12091386 (PMC10525328; doi:10.3390/antibiotics12091386)
Supplement: Supplementary file 1 [file antibiotics-12-01386-s001.zip › Table S1.pdf]

Table S1: Phenotypic Results

Cepheid ID	Sample type	State	Organism by K-mer spectra	mCIM (mm)	eCIM (mm)	mCIM/eCIM Result	FEP MIC	CTX MIC (µg/ml)	CAZ MIC (µg/ml)	CZA MIC (µg/ml)	CT MIC (µg/ml)	CRO MIC (µg/ml)	ETP MIC (µg/ml)	IPM MIC (µg/ml)	MEM MIC (µg/ml)	MEV MIC (µg/ml)
17978	Blood	GA	*Escherichia coli*	22	21	carbapenemase not detected	<=2	<=2	<=1	<=4	<=2	<=1	<=0.5	<=1	<=1	<=2
17977	Blood	GA	*Klebsiella pneumoniae*	6	6	serine carbapenemase detected	>16	>32	>16	<=4	>8	>32	>1	>8	>8	<=2
17976	Urine	GA	*Klebsiella pneumoniae*	11	18	serine carbapenemase detected	>16	>32	>16	<=4	>8	>32	>1	4	8	8
17975	Blood	GA	*Klebsiella oxytoca*	22	23	carbapenemase not detected	<=2	<=2	<=1	<=4	<=2	<=1	<=0.5	<=1	<=1	<=2
17974	Blood	GA	*Klebsiella pneumoniae*	6	6	serine carbapenemase detected	>16	>32	>16	<=4	>8	>32	>1	4	4	<=2
17973	Urine	GA	*Klebsiella pneumoniae*	6	6	serine carbapenemase detected	>16	>32	>16	<=4	>8	>32	>1	>8	>8	<=2
**17972**	Urine	GA	*Escherichia coli*	6	22	metallo-beta-lactamase detected	>16	>32	>16	>16	>8	>32	>1	4	8	8
17971	Urine	GA	*Escherichia coli*	6	6	serine carbapenemase detected	4	8	>16	<=4	4	>32	<=0.5	2	<=1	<=2
17970	Blood	GA	*Klebsiella michiganensis*	6	6	serine carbapenemase detected	16	16	>16	<=4	>8	32	>1	>8	4	<=2
17899	Blood	MO	*Klebsiella pneumoniae*	21	21	carbapenemase not detected	>16	>32	>16	<=4	8	>32	<=0.5	<=1	<=1	<=2
17898	Blood	MO	*Escherichia coli*	21	22	carbapenemase not detected	>16	>32	16	<=4	<=2	>32	<=0.5	<=1	<=1	<=2
17897	Blood	MO	*Escherichia coli*	20	24	carbapenemase not detected	<=2	<=2	8	<=4	<=2	<=1	<=0.5	<=1	<=1	<=2
17896	Urine	MO	*Escherichia coli*	21	23	carbapenemase not detected	>16	>32	4	<=4	<=2	>32	<=0.5	<=1	<=1	<=2
17895	Urine	MO	*Escherichia coli*	22	22	carbapenemase not detected	>16	>32	>16	<=4	<=2	>32	<=0.5	<=1	<=1	<=2
17894	Urine	MO	*Escherichia coli*	21	23	carbapenemase not detected	>16	>32	>16	<=4	<=2	>32	<=0.5	<=1	<=1	<=2
17893	Urine	MO	*Shigella flexneri*	21	21	carbapenemase not detected	>16	>32	>16	<=4	<=2	>32	<=0.5	<=1	<=1	<=2
17891	Blood	KS	*Escherichia coli*	21	23	carbapenemase not detected	16	>32	<=1	<=4	<=2	>32	<=0.5	<=1	<=1	<=2
17870	Blood	WI	*Escherichia coli*	21	22	carbapenemase not detected	>16	>32	>16	<=4	<=2	>32	<=0.5	<=1	<=1	<=2
17869	Blood	WI	*Klebsiella aerogenes*	21	22	carbapenemase not detected	<=2	>32	>16	<=4	8	>32	>1	4	<=1	<=2
17868	Blood	WI	*Escherichia coli*	22	23	carbapenemase not detected	<=2	32	4	<=4	<=2	32	<=0.5	<=1	<=1	<=2
17867	Blood	WI	*Raoultella ornithinolytica*	21	21	carbapenemase not detected	>16	>32	>16	<=4	>8	>32	>1	<=1	4	<=2

Table S1: Phenotypic Results

Cepheid ID	Sample type	State	Organism by K-mer spectra	mCIM (mm)	eCIM (mm)	mCIM/eCIM Result	FEP MIC	CTX MIC (µg/ml)	CAZ MIC (µg/ml)	CZA MIC (µg/ml)	CT MIC (µg/ml)	CRO MIC (µg/ml)	ETP MIC (µg/ml)	IPM MIC (µg/ml)	MEM MIC (µg/ml)	MEV MIC (µg/ml)
17866	Blood	WI	*Escherichia coli*	22	23	carbapenemase not detected	8	>32	4	<=4	<=2	>32	<=0.5	<=1	<=1	<=2
17865	Blood	WI	*Klebsiella pneumoniae*	21	22	carbapenemase not detected	>16	>32	>16	<=4	>8	>32	>1	2	4	<=2
17860	Blood	WI	*Escherichia coli*	22	22	carbapenemase not detected	>16	>32	8	<=4	<=2	>32	<=0.5	<=1	<=1	<=2
17859	Blood	WI	*Pseudomonas aeruginosa*	23	23	carbapenemase not detected	16	>32	>16	8	<=2	>32	>1	>8	>8	>16
17858	Blood	WI	*Escherichia coli*	20	22	carbapenemase not detected	>16	>32	>16	<=4	<=2	>32	<=0.5	<=1	<=1	<=2
17857	Blood	WI	*Escherichia coli*	20	22	carbapenemase not detected	>16	>32	>16	<=4	<=2	>32	<=0.5	<=1	<=1	<=2
17856	Blood	WI	*Escherichia coli*	23	23	carbapenemase not detected	>16	>32	16	<=4	<=2	>32	<=0.5	<=1	<=1	<=2
17855	Blood	WI	*Klebsiella pneumoniae*	21	21	carbapenemase not detected	>16	>32	<=1	<=4	<=2	>32	<=0.5	2	<=1	<=2
17854	Blood	WI	*Escherichia coli*	22	22	carbapenemase not detected	>16	>32	16	<=4	<=2	>32	<=0.5	<=1	<=1	<=2
17853	Blood	WI	*Klebsiella michiganensis*	6	6	serine carbapenemase detected	<=2	8	4	<=4	4	32	>1	4	4	<=2
17852	Blood	WI	*Klebsiella pneumoniae*	6	6	serine carbapenemase detected	>16	>32	>16	<=4	>8	>32	>1	>8	>8	<=2
17851	Urine	WI	*Klebsiella pneumoniae*	22	22	carbapenemase not detected	>16	>32	>16	<=4	<=2	>32	<=0.5	<=1	<=1	<=2
17850	Urine	WI	*Escherichia coli*	21	21	carbapenemase not detected	>16	>32	16	<=4	<=2	>32	<=0.5	<=1	<=1	<=2
17849	Urine	WI	*Escherichia coli*	21	21	carbapenemase not detected	16	>32	4	<=4	<=2	>32	<=0.5	<=1	<=1	<=2
17848	Urine	WI	*Escherichia coli*	21	22	carbapenemase not detected	>16	>32	16	<=4	<=2	>32	<=0.5	<=1	<=1	<=2
17847	Urine	WI	*Escherichia coli*	22	21	carbapenemase not detected	>16	>32	>16	<=4	<=2	>32	<=0.5	<=1	<=1	<=2
**17846**	Urine	WI	*Enterobacter sp.*	21	22	carbapenemase not detected	<=2	8	16	<=4	<=2	32	<=0.5	<=1	<=1	<=2
17845	Urine	WI	*Klebsiella pneumoniae*	21	21	carbapenemase not detected	>16	>32	>16	<=4	<=2	>32	<=0.5	<=1	<=1	<=2
**17844**	Urine	WI	*Enterobacter sp.*	22	22	carbapenemase not detected	>16	>32	>16	<=4	>8	>32	>1	>8	>8	4
17843	Urine	WI	*Providencia stuartii*	22	22	carbapenemase not detected	<=2	8	4	<=4	<=2	<=1	>1	4	4	<=2
17842	Urine	WI	*Klebsiella pneumoniae*	21	21	carbapenemase not detected	>16	>32	>16	<=4	>8	>32	>1	2	4	<=2

Table S1: Phenotypic Results

Cepheid ID	Sample type	State	Organism by K-mer spectra	mCIM (mm)	eCIM (mm)	mCIM/eCIM Result	FEP MIC	CTX MIC (µg/ml)	CAZ MIC (µg/ml)	CZA MIC (µg/ml)	CT MIC (µg/ml)	CRO MIC (µg/ml)	ETP MIC (µg/ml)	IPM MIC (µg/ml)	MEM MIC (µg/ml)	MEV MIC (µg/ml)
17841	Urine	WI	*Citrobacter freundii*	6	6	serine carbapenemase detected	>16	>32	>16	<=4	>8	>32	>1	>8	>8	<=2
17840	Urine	WI	*Klebsiella pneumoniae*	22	22	carbapenemase not detected	>16	>32	16	<=4	<=2	>32	<=0.5	<=1	<=1	<=2
17839	Urine	WI	*Escherichia coli*	22	22	carbapenemase not detected	>16	>32	16	<=4	<=2	>32	>1	<=1	<=1	<=2
17838	Urine	WI	*Citrobacter freundii*	6	6	serine carbapenemase detected	8	>32	>16	<=4	>8	>32	>1	8	8	<=2
17837	Urine	WI	*Klebsiella pneumoniae*	6	6	serine carbapenemase detected	>16	>32	>16	<=4	>8	>32	>1	>8	>8	<=2
17177	Urine	CA	*Acinetobacter baumannii*	24	24	carbapenemase not detected	>16	>32	>16	N/R	N/R	>32	>1	>8	>8	N/R
17835	Blood	IL	*Serratia nematodiphila*	21	21	carbapenemase not detected	16	>32	>16	>16	>8	>32	>1	2	<=1	<=2
17834	Blood	IL	*Escherichia coli*	21	22	carbapenemase not detected	>16	>32	>16	<=4	8	>32	<=0.5	<=1	<=1	<=2
17833	Blood	IL	*Klebsiella pneumoniae*	21	21	carbapenemase not detected	16	>32	4	<=4	<=2	>32	<=0.5	<=1	<=1	<=2
17832	Blood	IL	*Klebsiella pneumoniae*	6	6	serine carbapenemase detected	>16	>32	>16	<=4	>8	>32	>1	8	>8	<=2
17831	Blood	IL	*Escherichia coli*	21	21	carbapenemase not detected	>16	>32	>16	<=4	<=2	>32	<=0.5	<=1	<=1	<=2
17830	Blood	IL	*Escherichia coli*	21	22	carbapenemase not detected	>16	>32	4	<=4	<=2	>32	<=0.5	<=1	<=1	<=2
17829	Blood	IL	*Proteus mirabilis*	22	22	carbapenemase not detected	16	>32	<=1	<=4	<=2	>32	<=0.5	2	<=1	<=2
17828	Blood	IL	*Escherichia coli*	20	22	carbapenemase not detected	>16	>32	>16	<=4	<=2	>32	<=0.5	<=1	<=1	<=2
17827	Blood	IL	*Escherichia coli*	21	21	carbapenemase not detected	>16	>32	>16	<=4	<=2	>32	<=0.5	<=1	<=1	<=2
17826	Blood	IL	*Escherichia coli*	22	22	carbapenemase not detected	>16	>32	16	<=4	4	>32	<=0.5	<=1	<=1	<=2
**17825**	Blood	IL	*Enterobacter ludwigii*	22	22	carbapenemase not detected	4	16	>16	<=4	4	32	<=0.5	<=1	<=1	<=2
17824	Blood	IL	*Escherichia coli*	21	23	carbapenemase not detected	>16	>32	>16	<=4	<=2	>32	<=0.5	<=1	<=1	<=2
17823	Blood	IL	*Escherichia coli*	21	21	carbapenemase not detected	>16	>32	8	<=4	<=2	>32	<=0.5	<=1	<=1	<=2
17822	Blood	IL	*Escherichia coli*	22	22	carbapenemase not detected	>16	>32	>16	<=4	<=2	>32	<=0.5	<=1	<=1	<=2
17821	Urine	IL	*Escherichia coli*	22	22	carbapenemase not detected	<=2	32	>16	<=4	4	>32	<=0.5	<=1	<=1	<=2

Table S1: Phenotypic Results

Cepheid ID	Sample type	State	Organism by K-mer spectra	mCIM (mm)	eCIM (mm)	mCIM/eCIM Result	FEP MIC	CTX MIC (µg/ml)	CAZ MIC (µg/ml)	CZA MIC (µg/ml)	CT MIC (µg/ml)	CRO MIC (µg/ml)	ETP MIC (µg/ml)	IPM MIC (µg/ml)	MEM MIC (µg/ml)	MEV MIC (µg/ml)
17820	Urine	IL	*Escherichia coli*	22	23	carbapenemase not detected	>16	>32	8	<=4	<=2	>32	<=0.5	<=1	<=1	<=2
17819	Urine	IL	*Escherichia coli*	22	22	carbapenemase not detected	>16	>32	>16	<=4	<=2	>32	<=0.5	<=1	<=1	<=2
17818	Urine	IL	*Escherichia coli*	21	21	carbapenemase not detected	>16	>32	>16	<=4	<=2	>32	<=0.5	<=1	<=1	<=2
17817	Urine	IL	*Escherichia coli*	21	22	carbapenemase not detected	>16	>32	>16	<=4	<=2	>32	<=0.5	<=1	<=1	<=2
**17816**	Urine	IL	*Enterobacter hormaechei*	21	22	carbapenemase not detected	<=2	>32	>16	<=4	8	>32	>1	<=1	<=1	<=2
17815	Urine	IL	*Citrobacter freundii*	20	23	carbapenemase not detected	<=2	16	>16	<=4	<=2	32	<=0.5	<=1	<=1	<=2
17814	Urine	IL	*Escherichia coli*	22	21	carbapenemase not detected	>16	>32	16	<=4	<=2	>32	<=0.5	<=1	<=1	<=2
17813	Urine	IL	*Escherichia coli*	22	22	carbapenemase not detected	8	>32	<=1	<=4	<=2	>32	<=0.5	<=1	<=1	<=2
**17812**	Urine	IL	*Klebsiella pneumoniae*	21	21	carbapenemase not detected	>16	>32	8	<=4	<=2	>32	<=0.5	<=1	<=1	<=2
17811	Urine	IL	*Escherichia coli*	22	22	carbapenemase not detected	>16	>32	8	<=4	<=2	>32	<=0.5	<=1	<=1	<=2
17810	Urine	IL	*Escherichia coli*	22	22	carbapenemase not detected	>16	>32	>16	<=4	<=2	>32	<=0.5	<=1	<=1	<=2
17809	Urine	IL	*Escherichia coli*	23	22	carbapenemase not detected	>16	>32	>16	<=4	<=2	>32	<=0.5	<=1	<=1	<=2
17808	Urine	IL	*Escherichia coli*	22	22	carbapenemase not detected	>16	>32	>16	<=4	<=2	>32	<=0.5	<=1	<=1	<=2
17807	Urine	IL	*Providencia stuartii*	22	22	carbapenemase not detected	>16	16	>16	<=4	8	32	<=0.5	<=1	<=1	<=2
17755	Blood	TN	*Escherichia coli*	21	23	carbapenemase not detected	>16	>32	4	<=4	<=2	>32	<=0.5	<=1	<=1	<=2
17754	Blood	TN	*Pseudomonas aeruginosa*	22	23	carbapenemase not detected	8	>32	4	<=4	<=2	>32	>1	>8	>8	>16
17753	Blood	TN	*Citrobacter freundii*	6	6	serine carbapenemase detected	4	32	8	<=4	4	>32	>1	4	<=1	<=2
17752	Blood	TN	*Klebsiella pneumoniae*	20	22	carbapenemase not detected	>16	>32	4	<=4	<=2	>32	<=0.5	<=1	<=1	<=2
17751	Blood	TN	*Escherichia coli*	21	22	carbapenemase not detected	>16	>32	16	<=4	<=2	>32	<=0.5	<=1	<=1	<=2
17750	Blood	TN	*Escherichia coli*	20	21	carbapenemase not detected	>16	>32	>16	<=4	<=2	>32	<=0.5	<=1	<=1	<=2
17749	Blood	TN	*Pseudomonas aeruginosa*	22	22	carbapenemase not detected	>16	>32	8	8	<=2	32	>1	>8	>8	>16

Table S1: Phenotypic Results

Cepheid ID	Sample type	State	Organism by K-mer spectra	mCIM (mm)	eCIM (mm)	mCIM/eCIM Result	FEP MIC	CTX MIC (µg/ml)	CAZ MIC (µg/ml)	CZA MIC (µg/ml)	CT MIC (µg/ml)	CRO MIC (µg/ml)	ETP MIC (µg/ml)	IPM MIC (µg/ml)	MEM MIC (µg/ml)	MEV MIC (µg/ml)
17196	Blood	CA	*Acinetobacter baumannii*	22	22	carbapenemase not detected	16	>32	>16	N/R	N/R	>32	>1	4	8	8
17197	Blood	CA	*Acinetobacter baumannii*	22	22	carbapenemase not detected	16	>32	>16	16	4	>32	>1	8	>8	8
17746	Blood	TN	*Pseudomonas aeruginosa*	21	21	carbapenemase not detected	8	>32	8	8	<=2	>32	>1	>8	>8	16
17745	Blood	TN	*Pseudomonas aeruginosa*	22	23	carbapenemase not detected	>16	>32	>16	<=4	<=2	>32	>1	>8	>8	N/R
17744	Blood	TN	*Pseudomonas juntendi*	23	23	carbapenemase not detected	N/A	N/A	N/A	N/A	N/A	N/A	N/A	N/A	N/A	N/A
17743	Blood	TN	*Klebsiella pneumoniae*	6	22	metallo-beta-lactamase detected	>16	>32	>16	>16	>8	>32	>1	>8	>8	16
17742	Blood	TN	*Serratia marcescens*	6	21	metallo-beta-lactamase detected	16	>32	>16	>16	>8	32	1	>8	2	4
17741	Blood	TN	*Klebsiella pneumoniae*	6	22	metallo-beta-lactamase detected	>16	>32	>16	>16	>8	>32	>1	>8	>8	16
17740	Urine	TN	*Klebsiella pneumoniae*	21	22	carbapenemase not detected	>16	>32	16	<=4	<=2	>32	<=0.5	<=1	<=1	<=2
17739	Urine	TN	*Klebsiella aerogenes*	21	21	carbapenemase not detected	>16	32	>16	<=4	<=2	32	>1	2	2	<=2
**17738**	Urine	TN	*Enterobacter hormaechei*	21	21	carbapenemase not detected	8	>32	>16	<=4	>8	>32	>1	<=1	<=1	<=2
17737	Urine	TN	*Proteus mirabilis*	22	23	carbapenemase not detected	>16	>32	4	<=4	<=2	>32	<=0.5	4	<=1	<=2
17736	Urine	TN	*Klebsiella pneumoniae*	22	22	carbapenemase not detected	>16	>32	>16	<=4	<=2	>32	<=0.5	<=1	<=1	<=2
17735	Urine	TN	*Klebsiella pneumoniae*	21	21	carbapenemase not detected	>16	>32	16	<=4	<=2	>32	<=0.5	<=1	<=1	<=2
17734	Urine	TN	*Klebsiella pneumoniae*	20	20	carbapenemase not detected	>16	>32	>16	<=4	<=2	>32	<=0.5	<=1	<=1	<=2
17733	Urine	TN	*Escherichia coli*	21	22	carbapenemase not detected	>16	>32	>16	<=4	<=2	>32	<=0.5	<=1	<=1	<=2
17732	Urine	TN	*Klebsiella pneumoniae*	22	21	carbapenemase not detected	>16	>32	>16	<=4	<=2	>32	<=0.5	<=1	<=1	<=2
17731	Urine	TN	*Escherichia coli*	22	22	carbapenemase not detected	>16	>32	8	<=4	<=2	>32	<=0.5	<=1	<=1	<=2
17730	Urine	TN	*Escherichia coli*	22	22	carbapenemase not detected	>16	>32	8	<=4	<=2	>32	<=0.5	<=1	<=1	<=2
17729	Urine	TN	*Klebsiella pneumoniae*	22	22	carbapenemase not detected	>16	>32	16	<=4	<=2	>32	<=0.5	<=1	<=1	<=2
17728	Urine	TN	*Escherichia coli*	21	22	carbapenemase not detected	>16	>32	16	<=4	<=2	>32	<=0.5	<=1	<=1	<=2

Table S1: Phenotypic Results

Cepheid ID	Sample type	State	Organism by K-mer spectra	mCIM (mm)	eCIM (mm)	mCIM/eCIM Result	FEP MIC	CTX MIC (µg/ml)	CAZ MIC (µg/ml)	CZA MIC (µg/ml)	CT MIC (µg/ml)	CRO MIC (µg/ml)	ETP MIC (µg/ml)	IPM MIC (µg/ml)	MEM MIC (µg/ml)	MEV MIC (µg/ml)
17727	Urine	TN	*Escherichia coli*	21	21	carbapenemase not detected	>16	>32	>16	<=4	<=2	>32	<=0.5	<=1	<=1	<=2
17726	Urine	TN	*Klebsiella pneumoniae*	6	21	metallo-beta-lactamase detected	>16	>32	>16	>16	>8	>32	>1	>8	>8	16
17722	Blood	CA	*Escherichia coli*	23	24	carbapenemase not detected	>16	>32	16	<=4	<=2	>32	<=0.5	<=1	<=1	<=2
17721	Blood	CA	*Pseudomonas aeruginosa*	22	24	carbapenemase not detected	8	32	4	<=4	<=2	32	>1	>8	>8	8
17720	Blood	CA	*Pseudomonas aeruginosa*	24	24	carbapenemase not detected	8	>32	4	<=4	<=2	32	>1	>8	>8	8
17719	Blood	CA	*Pseudomonas aeruginosa*	24	23	carbapenemase not detected	16	>32	16	<=4	<=2	>32	>1	>8	>8	16
17718	Blood	CA	*Pseudomonas aeruginosa*	6	17	metallo-beta-lactamase detected	>16	>32	>16	>16	>8	>32	>1	>8	>8	>16
17717	Blood	CA	*Pseudomonas aeruginosa*	24	23	carbapenemase not detected	16	>32	>16	8	<=2	>32	>1	>8	>8	>16
17716	Urine	CA	*Klebsiella pneumoniae*	20	22	carbapenemase not detected	>16	>32	>16	<=4	<=2	>32	<=0.5	<=1	<=1	<=2
17715	Urine	CA	*Klebsiella pneumoniae*	22	22	carbapenemase not detected	16	>32	4	<=4	<=2	>32	<=0.5	<=1	<=1	<=2
**17714**	Urine	CA	*Enterobacter hormaechei*	21	23	carbapenemase not detected	>16	>32	>16	<=4	>8	>32	>1	2	2	<=2
17713	Urine	CA	*Escherichia coli*	23	23	carbapenemase not detected	4	32	4	<=4	<=2	32	>1	>8	8	8
17712	Urine	CA	*Klebsiella pneumoniae*	21	21	carbapenemase not detected	>16	>32	>16	<=4	<=2	>32	<=0.5	<=1	<=1	<=2
17316	Blood	NJ	*Acinetobacter baumannii*	24	24	carbapenemase not detected	>16	>32	>16	16	4	>32	>1	>8	>8	>16
17710	Urine	CA	*Pseudomonas aeruginosa*	23	23	carbapenemase not detected	8	>32	4	<=4	<=2	>32	>1	>8	>8	16
17709	Urine	CA	*Pseudomonas aeruginosa*	22	22	carbapenemase not detected	16	>32	>16	<=4	<=2	>32	>1	>8	>8	8
17708	Urine	CA	*Klebsiella aerogenes*	22	22	carbapenemase not detected	>16	>32	>16	>16	>8	>32	>1	>8	4	<=2
17707	Urine	CA	*Klebsiella pneumoniae*	21	21	carbapenemase not detected	>16	>32	>16	<=4	>8	>32	>1	<=1	2	<=2
17706	Urine	CA	*Klebsiella pneumoniae*	6	6	serine carbapenemase detected	>16	>32	>16	<=4	>8	>32	>1	>8	>8	<=2
17638	Blood	NM	*Escherichia coli*	21	22	carbapenemase not detected	>16	>32	16	<=4	<=2	>32	<=0.5	<=1	<=1	<=2
17637	Blood	NM	*Escherichia coli*	22	23	carbapenemase not detected	>16	>32	>16	<=4	<=2	>32	<=0.5	<=1	<=1	<=2

Table S1: Phenotypic Results

Cepheid ID	Sample type	State	Organism by K-mer spectra	mCIM (mm)	eCIM (mm)	mCIM/eCIM Result	FEP MIC	CTX MIC (µg/ml)	CAZ MIC (µg/ml)	CZA MIC (µg/ml)	CT MIC (µg/ml)	CRO MIC (µg/ml)	ETP MIC (µg/ml)	IPM MIC (µg/ml)	MEM MIC (µg/ml)	MEV MIC (µg/ml)
17636	Blood	NM	*Pseudomonas aeruginosa*	21	22	carbapenemase not detected	>16	>32	>16	>16	8	>32	>1	>8	>8	16
17324	Urine	GA	*Acinetobacter baumannii*	24	24	carbapenemase not detected	>16	8	4	8	4	8	>1	>8	>8	>16
17634	Blood	NM	*Pseudomonas aeruginosa*	23	22	carbapenemase not detected	>16	>32	>16	<=4	<=2	>32	>1	>8	>8	16
17628	Blood	OH	*Escherichia coli*	23	23	carbapenemase not detected	>16	>32	8	<=4	<=2	>32	<=0.5	<=1	<=1	<=2
**17627**	Blood	OH	*Enterobacter bugandensis*	22	22	carbapenemase not detected	<=2	<=2	<=1	<=4	<=2	<=1	<=0.5	<=1	<=1	<=2
17626	Blood	OH	*Escherichia coli*	23	23	carbapenemase not detected	>16	>32	>16	<=4	4	>32	<=0.5	<=1	<=1	<=2
17625	Blood	OH	*Escherichia coli*	21	21	carbapenemase not detected	8	>32	4	<=4	<=2	>32	<=0.5	<=1	<=1	<=2
17624	Blood	OH	*Escherichia coli*	20	20	carbapenemase not detected	>16	>32	<=1	<=4	<=2	>32	<=0.5	<=1	<=1	<=2
**17623**	Blood	OH	*Enterobacter hormaechei*	20	22	carbapenemase not detected	<=2	<=2	<=1	<=4	<=2	2	<=0.5	<=1	<=1	<=2
17622	Blood	OH	*Serratia marcescens*	20	20	carbapenemase not detected	<=2	<=2	<=1	<=4	<=2	<=1	<=0.5	<=1	<=1	<=2
17621	Blood	OH	*Serratia marcescens*	21	21	carbapenemase not detected	<=2	8	<=1	<=4	<=2	2	<=0.5	<=1	<=1	<=2
17620	Blood	OH	*Escherichia coli*	21	22	carbapenemase not detected	>16	>32	8	<=4	<=2	>32	<=0.5	<=1	<=1	<=2
17619	Blood	OH	*Klebsiella pneumoniae*	22	22	carbapenemase not detected	>16	>32	16	<=4	<=2	>32	<=0.5	<=1	<=1	<=2
**17618**	Blood	OH	*Enterobacter hormaechei*	21	21	carbapenemase not detected	<=2	<=2	<=1	<=4	<=2	<=1	<=0.5	<=1	<=1	<=2
17617	Blood	OH	*Escherichia coli*	23	23	carbapenemase not detected	>16	>32	>16	<=4	<=2	>32	<=0.5	<=1	<=1	<=2
**17616**	Blood	OH	*Enterobacter hormaechei [illegible]*	22	23	carbapenemase not detected	<=2	32	>16	<=4	<=2	>32	<=0.5	<=1	<=1	<=2
17615	Blood	OH	*Pseudomonas aeruginosa*	21	21	carbapenemase not detected	>16	>32	>16	<=4	>8	>32	>1	>8	8	4
17614	Blood	OH	*Pseudomonas aeruginosa*	21	21	carbapenemase not detected	8	16	4	<=4	<=2	8	>1	>8	>8	16
17613	Urine	OH	*Escherichia coli*	21	22	carbapenemase not detected	<=2	<=2	<=1	<=4	<=2	<=1	<=0.5	<=1	<=1	<=2
17612	Urine	OH	*Escherichia coli*	21	20	carbapenemase not detected	>16	>32	4	<=4	<=2	>32	<=0.5	<=1	<=1	<=2
17611	Urine	OH	*Escherichia coli*	20	20	carbapenemase not detected	>16	>32	>16	<=4	<=2	>32	<=0.5	<=1	<=1	<=2

Table S1: Phenotypic Results

Cepheid ID	Sample type	State	Organism by K-mer spectra	mCIM (mm)	eCIM (mm)	mCIM/eCIM Result	FEP MIC	CTX MIC (µg/ml)	CAZ MIC (µg/ml)	CZA MIC (µg/ml)	CT MIC (µg/ml)	CRO MIC (µg/ml)	ETP MIC (µg/ml)	IPM MIC (µg/ml)	MEM MIC (µg/ml)	MEV MIC (µg/ml)
17610	Urine	OH	*Escherichia coli*	22	22	carbapenemase not detected	>16	>32	>16	<=4	<=2	>32	<=0.5	<=1	<=1	<=2
**17609**	Urine	OH	*Enterobacter hormaechei* [illegible]	23	23	carbapenemase not detected	>16	>32	>16	<=4	8	>32	<=0.5	<=1	<=1	<=2
17608	Urine	OH	*Citrobacter freundii*	23	22	carbapenemase not detected	<=2	<=2	<=1	<=4	<=2	<=1	<=0.5	<=1	<=1	<=2
17607	Urine	OH	*Proteus mirabilis*	23	24	carbapenemase not detected	<=2	8	4	<=4	<=2	8	<=0.5	4	<=1	<=2
17606	Urine	OH	*Citrobacter freundii*	23	23	carbapenemase not detected	<=2	<=2	<=1	<=4	<=2	<=1	<=0.5	<=1	<=1	<=2
17605	Urine	OH	*Escherichia coli*	24	24	carbapenemase not detected	<=2	<=2	<=1	<=4	<=2	<=1	<=0.5	<=1	<=1	<=2
17604	Urine	OH	*Escherichia coli*	22	22	carbapenemase not detected	>16	>32	4	<=4	<=2	>32	<=0.5	<=1	<=1	<=2
**17603**	Urine	OH	*Enterobacter hormaechei*	22	21	carbapenemase not detected	<=2	<=2	<=1	<=4	<=2	<=1	<=0.5	<=1	<=1	<=2
17602	Urine	OH	*Escherichia coli*	22	22	carbapenemase not detected	>16	>32	16	<=4	<=2	>32	<=0.5	<=1	<=1	<=2
17601	Urine	OH	*Escherichia coli*	22	22	carbapenemase not detected	8	>32	4	<=4	<=2	>32	<=0.5	<=1	<=1	<=2
**17600**	Urine	OH	*Enterobacter hormaechei*	23	23	carbapenemase not detected	<=2	<=2	<=1	<=4	<=2	<=1	<=0.5	<=1	<=1	<=2
17599	Urine	OH	*Proteus penneri*	20	21	carbapenemase not detected	<=2	>32	<=1	<=4	<=2	>32	<=0.5	8	<=1	<=2
17598	Blood	WA	*Pseudomonas aeruginosa*	23	23	carbapenemase not detected	8	>32	16	<=4	<=2	>32	>1	>8	4	4
**17597**	Blood	WA	*Enterobacter cloacae complex*	20	21	carbapenemase not detected	16	>32	>16	<=4	>8	>32	1	<=1	<=1	<=2
17596	Blood	WA	*Proteus mirabilis*	21	21	carbapenemase not detected	>16	>32	<=1	<=4	<=2	>32	<=0.5	2	<=1	<=2
17595	Blood	WA	*Klebsiella pneumoniae*	20	20	carbapenemase not detected	>16	>32	16	<=4	<=2	>32	<=0.5	<=1	<=1	<=2
17590	Blood	MO	*Escherichia coli*	21	21	carbapenemase not detected	>16	>32	>16	<=4	<=2	>32	<=0.5	<=1	<=1	<=2
17589	Blood	MO	*Escherichia coli*	20	20	carbapenemase not detected	16	>32	8	<=4	<=2	>32	<=0.5	<=1	<=1	<=2
17588	Blood	MO	*Escherichia coli*	22	22	carbapenemase not detected	>16	>32	<=1	<=4	<=2	>32	<=0.5	<=1	<=1	<=2
17587	Blood	MO	*Escherichia coli*	20	22	carbapenemase not detected	>16	>32	8	<=4	<=2	>32	<=0.5	<=1	<=1	<=2
17586	Blood	MO	*Escherichia coli*	22	23	carbapenemase not detected	>16	>32	8	<=4	<=2	>32	<=0.5	<=1	<=1	<=2

Table S1: Phenotypic Results

Cepheid ID	Sample type	State	Organism by K-mer spectra	mCIM (mm)	eCIM (mm)	mCIM/eCIM Result	FEP MIC	CTX MIC (µg/ml)	CAZ MIC (µg/ml)	CZA MIC (µg/ml)	CT MIC (µg/ml)	CRO MIC (µg/ml)	ETP MIC (µg/ml)	IPM MIC (µg/ml)	MEM MIC (µg/ml)	MEV MIC (µg/ml)
17585	Blood	MO	*Escherichia coli*	20	22	carbapenemase not detected	16	32	>16	<=4	<=2	>32	<=0.5	<=1	<=1	<=2
17584	Blood	MO	*Escherichia coli*	21	23	carbapenemase not detected	>16	>32	>16	<=4	<=2	>32	<=0.5	<=1	<=1	<=2
17583	Blood	MO	*Escherichia coli*	21	21	carbapenemase not detected	>16	>32	8	<=4	<=2	>32	<=0.5	<=1	<=1	<=2
17582	Urine	MO	*Klebsiella pneumoniae*	21	21	carbapenemase not detected	8	>32	4	<=4	<=2	>32	<=0.5	<=1	<=1	<=2
17581	Urine	MO	*Escherichia coli*	21	21	carbapenemase not detected	>16	>32	<=1	<=4	<=2	>32	<=0.5	<=1	<=1	<=2
17580	Urine	MO	*Escherichia coli*	20	21	carbapenemase not detected	>16	>32	>16	<=4	<=2	>32	<=0.5	<=1	<=1	<=2
17579	Urine	MO	*Klebsiella pneumoniae*	19	20	carbapenemase not detected	>16	>32	>16	<=4	<=2	>32	<=0.5	<=1	<=1	<=2
17578	Urine	MO	*Klebsiella pneumoniae*	22	22	carbapenemase not detected	>16	>32	16	<=4	<=2	>32	<=0.5	<=1	<=1	<=2
17577	Urine	MO	*Klebsiella pneumoniae*	22	22	carbapenemase not detected	>16	>32	16	<=4	<=2	>32	<=0.5	<=1	<=1	<=2
17576	Urine	MO	*Escherichia coli*	22	22	carbapenemase not detected	>16	>32	>16	<=4	<=2	>32	<=0.5	<=1	<=1	<=2
17575	Urine	MO	*Escherichia coli*	22	22	carbapenemase not detected	<=2	<=2	4	<=4	8	<=1	<=0.5	<=1	<=1	<=2
17574	Urine	MO	*Escherichia coli*	23	23	carbapenemase not detected	>16	>32	>16	<=4	<=2	>32	<=0.5	<=1	<=1	<=2
17573	Urine	MO	*Escherichia coli*	20	22	carbapenemase not detected	>16	>32	16	<=4	<=2	>32	<=0.5	<=1	<=1	<=2
17572	Urine	MO	*Klebsiella pneumoniae*	20	22	carbapenemase not detected	16	>32	4	<=4	<=2	>32	<=0.5	<=1	<=1	<=2
**17571**	Blood	WA	*Enterobacter hormaechei* [illegible]	20	21	carbapenemase not detected	4	>32	>16	<=4	8	>32	<=0.5	<=1	<=1	<=2
17570	Blood	WA	*Pseudomonas aeruginosa*	20	22	carbapenemase not detected	16	>32	8	8	<=2	>32	>1	>8	>8	>16
17569	Blood	WA	*Escherichia coli*	21	23	carbapenemase not detected	<=2	16	16	<=4	<=2	32	<=0.5	<=1	<=1	<=2
17568	Blood	WA	*Escherichia coli*	21	21	carbapenemase not detected	>16	>32	>16	<=4	<=2	>32	<=0.5	<=1	<=1	<=2
17567	Blood	WA	*Escherichia coli*	21	21	carbapenemase not detected	>16	>32	>16	<=4	<=2	>32	<=0.5	<=1	<=1	<=2
17566	Blood	WA	*Escherichia coli*	21	22	carbapenemase not detected	>16	>32	>16	<=4	<=2	>32	<=0.5	<=1	<=1	<=2
17565	Blood	WA	*Serratia marcescens*	21	22	carbapenemase not detected	<=2	<=2	<=1	<=4	<=2	<=1	<=0.5	<=1	<=1	<=2

Table S1: Phenotypic Results

Cepheid ID	Sample type	State	Organism by K-mer spectra	mCIM (mm)	eCIM (mm)	mCIM/eCIM Result	FEP MIC	CTX MIC (µg/ml)	CAZ MIC (µg/ml)	CZA MIC (µg/ml)	CT MIC (µg/ml)	CRO MIC (µg/ml)	ETP MIC (µg/ml)	IPM MIC (µg/ml)	MEM MIC (µg/ml)	MEV MIC (µg/ml)
17564	Blood	WA	*Escherichia coli*	20	21	carbapenemase not detected	16	>32	<=1	<=4	<=2	>32	<=0.5	<=1	<=1	<=2
17563	Blood	WA	*Pseudomonas aeruginosa*	20	21	carbapenemase not detected	>16	>32	>16	16	<=2	>32	>1	>8	>8	>16
17562	Blood	WA	*Klebsiella michiganensis*	21	21	carbapenemase not detected	<=2	<=2	<=1	<=4	<=2	32	<=0.5	<=1	<=1	<=2
17561	Blood	WA	*Klebsiella pneumoniae*	20	20	carbapenemase not detected	>16	>32	>16	<=4	<=2	>32	<=0.5	<=1	<=1	<=2
17560	Urine	WA	*Escherichia coli*	21	22	carbapenemase not detected	>16	>32	>16	<=4	<=2	>32	<=0.5	<=1	<=1	<=2
**17559**	Urine	WA	*Enterobacter cloacae complex*	21	22	carbapenemase not detected	16	>32	4	<=4	<=2	>32	<=0.5	<=1	<=1	<=2
17558	Urine	WA	*Escherichia coli*	23	23	carbapenemase not detected	>16	>32	>16	<=4	<=2	>32	<=0.5	<=1	<=1	<=2
17557	Urine	WA	*Escherichia coli*	23	23	carbapenemase not detected	<=2	>32	<=1	<=4	<=2	>32	<=0.5	<=1	<=1	<=2
17556	Urine	WA	*Escherichia coli*	21	22	carbapenemase not detected	4	>32	4	<=4	<=2	>32	<=0.5	<=1	<=1	<=2
17555	Urine	WA	*Escherichia coli*	21	21	carbapenemase not detected	>16	>32	4	<=4	<=2	>32	<=0.5	<=1	<=1	<=2
17554	Urine	WA	*Escherichia coli*	21	21	carbapenemase not detected	>16	>32	8	<=4	<=2	>32	<=0.5	<=1	<=1	<=2
17553	Urine	WA	*Klebsiella pneumoniae*	21	22	carbapenemase not detected	>16	>32	>16	<=4	<=2	>32	<=0.5	<=1	<=1	<=2
17552	Urine	WA	*Escherichia coli*	22	22	carbapenemase not detected	>16	>32	<=1	<=4	<=2	>32	<=0.5	<=1	<=1	<=2
17551	Urine	WA	*Pseudomonas aeruginosa*	22	22	carbapenemase not detected	8	16	4	<=4	<=2	32	>1	>8	4	N/R
**17550**	Urine	WA	*Enterobacter hormaechei*	20	21	carbapenemase not detected	<=2	>32	16	<=4	<=2	32	>1	<=1	<=1	<=2
**17549**	Urine	WA	*Enterobacter hormaechei*	20	21	carbapenemase not detected	>16	>32	16	<=4	<=2	>32	<=0.5	<=1	<=1	<=2
17548	Urine	WA	*Klebsiella pneumoniae*	22	21	carbapenemase not detected	>16	>32	>16	<=4	<=2	>32	<=0.5	<=1	<=1	<=2
17547	Urine	WA	*Klebsiella pneumoniae*	20	21	carbapenemase not detected	>16	>32	16	<=4	<=2	>32	<=0.5	<=1	<=1	<=2
17546	Urine	WA	*Escherichia coli*	20	21	carbapenemase not detected	>16	>32	>16	<=4	<=2	>32	<=0.5	<=1	<=1	<=2
17545	Blood	NM	*Escherichia coli*	21	22	carbapenemase not detected	>16	>32	<=1	<=4	<=2	>32	<=0.5	<=1	<=1	<=2
17544	Blood	NM	*Escherichia coli*	21	21	carbapenemase not detected	>16	>32	>16	<=4	<=2	>32	<=0.5	<=1	<=1	<=2

Table S1: Phenotypic Results

Cepheid ID	Sample type	State	Organism by K-mer spectra	mCIM (mm)	eCIM (mm)	mCIM/eCIM Result	FEP MIC	CTX MIC (µg/ml)	CAZ MIC (µg/ml)	CZA MIC (µg/ml)	CT MIC (µg/ml)	CRO MIC (µg/ml)	ETP MIC (µg/ml)	IPM MIC (µg/ml)	MEM MIC (µg/ml)	MEV MIC (µg/ml)
17543	Blood	NM	*Escherichia coli*	20	20	carbapenemase not detected	>16	>32	8	<=4	<=2	>32	<=0.5	<=1	<=1	<=2
17329	Urine	GA	*Acinetobacter baumannii*	22	22	carbapenemase not detected	>16	>32	>16	16	>8	>32	>1	>8	>8	>16
17541	Blood	NM	*Klebsiella pneumoniae*	21	21	carbapenemase not detected	<=2	<=2	<=1	<=4	<=2	<=1	<=0.5	<=1	<=1	<=2
17540	Blood	NM	*Klebsiella pneumoniae*	20	20	carbapenemase not detected	<=2	32	>16	<=4	<=2	32	<=0.5	<=1	<=1	<=2
17539	Blood	NM	*Escherichia coli*	20	20	carbapenemase not detected	>16	>32	16	<=4	<=2	>32	<=0.5	<=1	<=1	<=2
17538	Blood	NM	*Escherichia coli*	20	21	carbapenemase not detected	>16	>32	>16	<=4	<=2	>32	<=0.5	<=1	<=1	<=2
17537	Blood	NM	*Escherichia coli*	20	20	carbapenemase not detected	>16	>32	>16	<=4	<=2	>32	<=0.5	<=1	<=1	<=2
**17536**	Blood	NM	*Enterobacter hormaechei* [illegible]	20	23	carbapenemase not detected	16	>32	>16	<=4	>8	>32	>1	<=1	<=1	<=2
17535	Urine	NM	*Klebsiella pneumoniae*	20	20	carbapenemase not detected	>16	>32	>16	<=4	<=2	>32	<=0.5	<=1	<=1	<=2
**17534**	Urine	NM	*Enterobacter hormaechei*	21	22	carbapenemase not detected	16	>32	>16	<=4	>8	>32	>1	<=1	<=1	<=2
17533	Urine	NM	*Pseudomonas aeruginosa*	21	22	carbapenemase not detected	16	>32	16	<=4	<=2	>32	>1	4	2	<=2
17531	Urine	NM	*Klebsiella pneumoniae*	20	20	carbapenemase not detected	>16	>32	16	<=4	<=2	>32	1	<=1	<=1	<=2
17530	Urine	NM	*Pseudomonas aeruginosa*	21	22	carbapenemase not detected	8	>32	4	<=4	<=2	32	>1	>8	>8	16
**17529**	Urine	NM	*Enterobacter cloacae complex*	21	22	carbapenemase not detected	<=2	<=2	<=1	<=4	<=2	2	<=0.5	<=1	<=1	<=2
17528	Urine	NM	*Hafnia alvei*	20	21	carbapenemase not detected	<=2	16	>16	<=4	4	32	<=0.5	<=1	<=1	<=2
17527	Urine	NM	*Klebsiella michiganensis*	22	22	carbapenemase not detected	>16	>32	>16	<=4	>8	>32	<=0.5	<=1	<=1	<=2
**17526**	Urine	NM	*Enterobacter cloacae complex*	21	22	carbapenemase not detected	8	>32	>16	<=4	8	>32	>1	<=1	<=1	<=2
**17525**	Urine	NM	*Enterobacter hormaechei*	6	23	metallo-beta-lactamase detected	>16	>32	>16	>16	>8	>32	>1	>8	>8	>16
17524	Urine	NM	*Pseudomonas aeruginosa*	23	23	carbapenemase not detected	<=2	>32	4	<=4	<=2	>32	>1	>8	4	4
17523	Urine	NM	*Raoultella ornithinolytica*	6	6	serine carbapenemase detected	<=2	8	<=1	<=4	<=2	2	>1	4	2	<=2
17522	Urine	NM	*Escherichia coli*	22	22	carbapenemase not detected	>16	>32	>16	<=4	<=2	>32	<=0.5	<=1	<=1	<=2

Table S1: Phenotypic Results

Cepheid ID	Sample type	State	Organism by K-mer spectra	mCIM (mm)	eCIM (mm)	mCIM/eCIM Result	FEP MIC	CTX MIC (µg/ml)	CAZ MIC (µg/ml)	CZA MIC (µg/ml)	CT MIC (µg/ml)	CRO MIC (µg/ml)	ETP MIC (µg/ml)	IPM MIC (µg/ml)	MEM MIC (µg/ml)	MEV MIC (µg/ml)
17521	Urine	NM	*Klebsiella pneumoniae*	20	20	carbapenemase not detected	>16	>32	16	<=4	<=2	>32	<=0.5	<=1	<=1	<=2
17520	Urine	NM	*Escherichia coli*	20	22	carbapenemase not detected	>16	>32	>16	<=4	<=2	>32	<=0.5	<=1	<=1	<=2
17464	Blood	NY	*Escherichia coli*	23	23	carbapenemase not detected	<=2	>32	8	<=4	<=2	>32	<=0.5	<=1	<=1	<=2
17463	Blood	NY	*Escherichia coli*	24	24	carbapenemase not detected	>16	>32	8	<=4	<=2	>32	<=0.5	<=1	<=1	<=2
17462	Blood	NY	*Escherichia coli*	23	23	carbapenemase not detected	<=2	>32	<=1	<=4	<=2	>32	<=0.5	<=1	<=1	<=2
**17461**	Blood	NY	*Enterobacter ludwigii*	6 (inner col)	23	serine carbapenemase detected	<=2	16	>16	<=4	<=2	32	<=0.5	<=1	<=1	<=2
17460	Blood	NY	*Serratia fonticola*	23	23	carbapenemase not detected	4	>32	4	<=4	<=2	>32	<=0.5	2	<=1	<=2
17459	Blood	NY	*Escherichia coli*	23	23	carbapenemase not detected	>16	>32	16	<=4	<=2	>32	<=0.5	<=1	<=1	<=2
17458	Blood	NY	*Escherichia coli*	23	23	carbapenemase not detected	8	>32	4	<=4	<=2	>32	<=0.5	<=1	<=1	<=2
17457	Blood	NY	*Escherichia coli*	22	22	carbapenemase not detected	<=2	8	16	<=4	<=2	32	<=0.5	<=1	<=1	<=2
17456	Blood	NY	*Citrobacter freundii*	21	23	carbapenemase not detected	<=2	>32	>16	<=4	>8	>32	<=0.5	<=1	<=1	<=2
17455	Blood	NY	*Escherichia coli*	22	23	carbapenemase not detected	>16	>32	8	<=4	<=2	>32	<=0.5	<=1	<=1	<=2
17454	Blood	NY	*Escherichia coli*	22	23	carbapenemase not detected	>16	>32	>16	<=4	<=2	>32	<=0.5	<=1	<=1	<=2
17453	Blood	NY	*Klebsiella aerogenes*	21	22	carbapenemase not detected	<=2	8	16	<=4	<=2	8	<=0.5	<=1	<=1	<=2
17452	Blood	NY	*Escherichia coli*	22	23	carbapenemase not detected	>16	>32	>16	<=4	<=2	>32	<=0.5	<=1	<=1	<=2
17451	Blood	NY	*Escherichia coli*	22	23	carbapenemase not detected	>16	>32	4	<=4	<=2	>32	<=0.5	<=1	<=1	<=2
17450	Blood	NY	*Klebsiella pneumoniae*	22	22	carbapenemase not detected	>16	>32	>16	<=4	<=2	>32	<=0.5	<=1	<=1	<=2
17449	Urine	NY	*Klebsiella pneumoniae*	22	22	carbapenemase not detected	>16	>32	>16	<=4	>8	>32	<=0.5	<=1	<=1	<=2
17448	Urine	NY	*Klebsiella michiganensis*	22	22	carbapenemase not detected	>16	>32	16	<=4	<=2	>32	<=0.5	<=1	<=1	<=2
17447	Urine	NY	*Escherichia coli*	21	23	carbapenemase not detected	>16	>32	>16	<=4	<=2	>32	<=0.5	<=1	<=1	<=2
17446	Urine	NY	*Escherichia coli*	22	23	carbapenemase not detected	>16	>32	>16	<=4	<=2	>32	<=0.5	<=1	<=1	<=2

Table S1: Phenotypic Results

Cepheid ID	Sample type	State	Organism by K-mer spectra	mCIM (mm)	eCIM (mm)	mCIM/eCIM Result	FEP MIC	CTX MIC (µg/ml)	CAZ MIC (µg/ml)	CZA MIC (µg/ml)	CT MIC (µg/ml)	CRO MIC (µg/ml)	ETP MIC (µg/ml)	IPM MIC (µg/ml)	MEM MIC (µg/ml)	MEV MIC (µg/ml)
17445	Urine	NY	*Escherichia coli*	21	22	carbapenemase not detected	>16	>32	4	<=4	<=2	>32	<=0.5	<=1	<=1	<=2
17444	Urine	NY	*Escherichia coli*	22	22	carbapenemase not detected	>16	>32	<=1	<=4	<=2	>32	<=0.5	<=1	<=1	<=2
17443	Urine	NY	*Klebsiella pneumoniae*	6	6	serine carbapenemase detected	>16	>32	>16	<=4	>8	>32	>1	4	4	<=2
17442	Urine	NY	*Klebsiella pneumoniae*	6	6	serine carbapenemase detected	>16	>32	>16	<=4	>8	>32	>1	2	<=1	<=2
17330	Urine	GA	*Acinetobacter baumannii*	22 (2 col)	22	carbapenemase not detected	>16	>32	>16	>16	>8	>32	>1	>8	>8	>16
**17440**	Urine	NY	*Enterobacter hormaechei*	23	23	carbapenemase not detected	>16	>32	>16	<=4	>8	>32	>1	4	4	<=2
**17439**	Urine	NY	*Klebsiella pneumoniae*	6	6	serine carbapenemase detected	16	16	>16	<=4	>8	>32	>1	>8	>8	<=2
17339	Blood	GA	*Acinetobacter baumannii*	23	23	carbapenemase not detected	>16	>32	>16	>16	>8	>32	>1	>8	>8	>16
**17437**	Urine	NY	*Enterobacter hormaechei*	22	22	carbapenemase not detected	8	>32	>16	<=4	>8	>32	>1	<=1	<=1	<=2
17435	Urine	NY	*Acinetobacter baumannii*	22	22	carbapenemase not detected	16	16	4	8	<=2	8	>1	>8	>8	16
17436	Urine	NY	*Acinetobacter baumannii*	22	22	carbapenemase not detected	>16	>32	>16	>16	>8	>32	>1	>8	>8	>16
17430	Blood	NJ	*Escherichia coli*	20 (some [illegible]	23	carbapenemase not detected	<=2	32	>16	<=4	<=2	8	<=0.5	<=1	<=1	<=2
17429	Blood	NJ	*Escherichia coli*	22	22	carbapenemase not detected	<=2	<=2	4	<=4	<=2	8	<=0.5	<=1	<=1	<=2
17428	Blood	NJ	*Escherichia coli*	22	22	carbapenemase not detected	>16	>32	8	<=4	<=2	>32	<=0.5	<=1	<=1	<=2
**17427**	Blood	NJ	*Klebsiella pneumoniae*	22	22	carbapenemase not detected	>16	>32	16	<=4	<=2	>32	<=0.5	<=1	<=1	<=2
17426	Blood	NJ	*Escherichia coli*	22	22	carbapenemase not detected	>16	>32	>16	<=4	<=2	>32	<=0.5	<=1	<=1	<=2
17425	Blood	NJ	*Escherichia coli*	21	21	carbapenemase not detected	>16	>32	8	<=4	<=2	>32	<=0.5	<=1	<=1	<=2
17424	Blood	NJ	*Pseudomonas aeruginosa*	23	23	carbapenemase not detected	>16	>32	>16	<=4	<=2	>32	>1	>8	8	N/R
17346	Blood	KS	*Escherichia coli*	21	22	carbapenemase not detected	>16	>32	16	<=4	<=2	>32	<=0.5	<=1	<=1	<=2
**17345**	Urine	KS	*Klebsiella pneumoniae*	21	22	carbapenemase not detected	>16	>32	8	<=4	<=2	>32	<=0.5	<=1	<=1	<=2
**17344**	Urine	KS	*Klebsiella pneumoniae*	20	23	carbapenemase not detected	>16	>32	>16	<=4	<=2	>32	1	<=1	<=1	<=2

Table S1: Phenotypic Results

Cepheid ID	Sample type	State	Organism by K-mer spectra	mCIM (mm)	eCIM (mm)	mCIM/eCIM Result	FEP MIC	CTX MIC (µg/ml)	CAZ MIC (µg/ml)	CZA MIC (µg/ml)	CT MIC (µg/ml)	CRO MIC (µg/ml)	ETP MIC (µg/ml)	IPM MIC (µg/ml)	MEM MIC (µg/ml)	MEV MIC (µg/ml)
17343	Urine	KS	*Escherichia coli*	21	21	carbapenemase not detected	>16	>32	16	<=4	<=2	>32	<=0.5	<=1	<=1	<=2
17342	Urine	KS	*Escherichia coli*	21	21	carbapenemase not detected	>16	>32	16	<=4	<=2	>32	<=0.5	<=1	<=1	<=2
**17340**	Blood	GA	*Klebsiella pneumoniae*	6	18 (pinpoint)	metallo-beta-lactamase detected	>16	>32	>16	>16	>8	>32	>1	>8	>8	>16
17438	Urine	NY	*Acinetobacter baumannii*	22	22	carbapenemase not detected	>16	>32	>16	>16	>8	>32	>1	8	8	8
**17338**	Urine	GA	*Klebsiella oxytoca*	6	6	serine carbapenemase detected	<=2	<=2	8	<=4	4	8	<=0.5	4	<=1	<=2
**17337**	Urine	GA	*Klebsiella pneumoniae*	6	6	serine carbapenemase detected	>16	>32	>16	<=4	8	>32	>1	4	<=1	<=2
**17336**	Blood	GA	*Klebsiella pneumoniae*	6	6	serine carbapenemase detected	8	16	>16	<=4	>8	>32	>1	4	4	<=2
17335	Urine	GA	*Klebsiella aerogenes*	6	6	serine carbapenemase detected	>16	>32	>16	<=4	8	>32	>1	4	4	<=2
**17334**	Urine	GA	*Klebsiella pneumoniae*	6	6	serine carbapenemase detected	>16	>32	>16	<=4	8	>32	>1	4	<=1	<=2
17333	Urine	GA	*Klebsiella aerogenes*	6	6	serine carbapenemase detected	>16	>32	>16	<=4	>8	>32	>1	4	4	<=2
**17332**	Urine	GA	*Klebsiella pneumoniae*	22	22	carbapenemase not detected	>16	>32	>16	<=4	8	>32	>1	<=1	2	<=2
17331	Urine	GA	*Klebsiella pneumoniae*	6	18 (pinpoint)	metallo-beta-lactamase detected	>16	>32	>16	>16	>8	>32	>1	>8	>8	>16
17441	Urine	NY	*Acinetobacter baumannii*	23	23	carbapenemase not detected	>16	>32	>16	>16	>8	>32	>1	>8	>8	16
17542	Blood	NM	*Acinetobacter beijerinckii*	21	23	carbapenemase not detected	<=2	<=2	<=1	<=4	<=2	<=1	<=0.5	<=1	<=1	<=2
**17328**	Urine	GA	*Enterobacter hormaechei*	22	22	carbapenemase not detected	8	>32	>16	<=4	>8	>32	>1	<=1	<=1	<=2
**17327**	Urine	GA	*Klebsiella pneumoniae*	6	6	serine carbapenemase detected	16	32	>16	<=4	8	>32	>1	8	4	<=2
**17326**	Urine	GA	*Klebsiella oxytoca*	22	22	carbapenemase not detected	>16	>32	>16	<=4	>8	>32	>1	<=1	4	<=2
**17325**	Urine	GA	*Klebsiella pneumoniae*	6	6	serine carbapenemase detected	16	>32	>16	<=4	>8	>32	>1	4	4	<=2
17635	Blood	NM	*Acinetobacter baumannii*	23	22	carbapenemase not detected	4	16	4	8	<=2	32	>1	<=1	<=1	<=2
**17323**	Blood	NJ	*Klebsiella pneumoniae*	22	24	carbapenemase not detected	>16	>32	>16	<=4	>8	>32	>1	<=1	<=1	<=2
**17322**	Blood	NJ	*Klebsiella pneumoniae*	6	6	serine carbapenemase detected	>16	>32	>16	<=4	>8	>32	>1	>8	>8	<=2

Table S1: Phenotypic Results

Cepheid ID	Sample type	State	Organism by K-mer spectra	mCIM (mm)	eCIM (mm)	mCIM/eCIM Result	FEP MIC	CTX MIC (µg/ml)	CAZ MIC (µg/ml)	CZA MIC (µg/ml)	CT MIC (µg/ml)	CRO MIC (µg/ml)	ETP MIC (µg/ml)	IPM MIC (µg/ml)	MEM MIC (µg/ml)	MEV MIC (µg/ml)
17321	Blood	NJ	*Klebsiella aerogenes*	21	21	carbapenemase not detected	8	>32	>16	<=4	>8	>32	>1	>8	>8	8
**17320**	Blood	NJ	*Klebsiella pneumoniae*	21	21	carbapenemase not detected	>16	>32	>16	<=4	>8	>32	>1	<=1	2	<=2
17319	Blood	NJ	*Escherichia coli*	6	6	serine carbapenemase detected	>16	>32	>16	<=4	>8	>32	>1	<=1	<=1	<=2
17318	Blood	NJ	*Escherichia coli*	23	23	carbapenemase not detected	>16	>32	>16	<=4	<=2	>32	<=0.5	<=1	<=1	<=2
**17317**	Blood	NJ	*Enterobacter cloacae*	21	22	carbapenemase not detected	8	>32	>16	<=4	>8	>32	<=0.5	<=1	<=1	<=2
17711	Urine	CA	*Acinetobacter baumannii*	21	21	carbapenemase not detected	>16	>32	>16	>16	>8	32	>1	>8	>8	>16
17315	Urine	NJ	*Pseudomonas aeruginosa*	23	23	carbapenemase not detected	16	>32	8	8	<=2	>32	>1	<=1	2	N/R
**17314**	Urine	NJ	*Klebsiella pneumoniae*	6	6	serine carbapenemase detected	>16	>32	>16	<=4	>8	>32	>1	>8	>8	<=2
**17313**	Urine	NJ	*Klebsiella pneumoniae*	6	6	serine carbapenemase detected	>16	>32	>16	<=4	>8	>32	>1	>8	>8	<=2
17312	Urine	NJ	*Escherichia coli*	23	23	carbapenemase not detected	>16	>32	>16	<=4	<=2	>32	<=0.5	<=1	<=1	<=2
**17311**	Urine	NJ	*Klebsiella pneumoniae*	6	6	serine carbapenemase detected	4	8	4	<=4	4	>32	>1	8	8	<=2
**17310**	Urine	NJ	*Enterobacter cloacae*	20	21	carbapenemase not detected	<=2	32	16	<=4	<=2	32	<=0.5	<=1	<=1	<=2
17309	Urine	NJ	*Escherichia coli*	20	21	carbapenemase not detected	>16	>32	16	<=4	<=2	>32	<=0.5	<=1	<=1	<=2
**17308**	Urine	NJ	*Enterobacter cloacae*	21	23	carbapenemase not detected	4	>32	>16	<=4	8	>32	<=0.5	<=1	<=1	<=2
**17307**	Urine	NJ	*Enterobacter hormaechei* [illegible]	6	6	serine carbapenemase detected	>16	>32	>16	<=4	>8	>32	>1	>8	>8	<=2
**17306**	Urine	NJ	*Klebsiella pneumoniae*	6	6	serine carbapenemase detected	4	8	8	<=4	4	>32	>1	8	8	<=2
17305	Urine	NJ	*Escherichia coli*	22	22	carbapenemase not detected	>16	>32	16	<=4	<=2	>32	<=0.5	<=1	<=1	<=2
**17304**	Urine	NJ	*Klebsiella pneumoniae*	6	6	serine carbapenemase detected	>16	>32	>16	<=4	>8	>32	>1	>8	>8	<=2
17303	Urine	NJ	*Klebsiella aerogenes*	20	21	carbapenemase not detected	8	>32	>16	<=4	8	>32	>1	>8	8	4
17302	Urine	NJ	*Pseudomonas aeruginosa*	22	22	carbapenemase not detected	16	>32	8	8	<=2	>32	>1	>8	>8	>16
17301	Urine	NJ	*Pseudomonas aeruginosa*	22	22	carbapenemase not detected	16	>32	8	8	<=2	>32	>1	<=1	2	4

Table S1: Phenotypic Results

Cepheid ID	Sample type	State	Organism by K-mer spectra	mCIM (mm)	eCIM (mm)	mCIM/eCIM Result	FEP MIC	CTX MIC (µg/ml)	CAZ MIC (µg/ml)	CZA MIC (µg/ml)	CT MIC (µg/ml)	CRO MIC (µg/ml)	ETP MIC (µg/ml)	IPM MIC (µg/ml)	MEM MIC (µg/ml)	MEV MIC (µg/ml)
17247	Urine	KS	*Pseudomonas aeruginosa*	22	22	carbapenemase not detected	8	>32	4	<=4	<=2	32	>1	8	>8	16
**17246**	Urine	KS	*Enterobacter cloacae*	21	21	carbapenemase not detected	<=2	<=2	4	<=4	<=2	<=1	>1	8	2	<=2
17245	Urine	KS	*Serratia marcescens*	22	21	carbapenemase not detected	8	>32	4	<=4	<=2	32	>1	2	<=1	<=2
17244	Urine	KS	*Escherichia coli*	22	22	carbapenemase not detected	>16	>32	4	<=4	<=2	>32	<=0.5	<=1	<=1	<=2
17243	Urine	KS	*Escherichia coli*	22	21	carbapenemase not detected	>16	>32	>16	<=4	<=2	>32	<=0.5	<=1	<=1	<=2
17213	Urine	KS	*Providencia rettgeri*	22	22	carbapenemase not detected	>16	>32	>16	>16	>8	>32	>1	>8	>8	16
17212	Urine	KS	*Escherichia coli*	22	22	carbapenemase not detected	<=2	>32	4	<=4	<=2	>32	<=0.5	<=1	<=1	<=2
17211	Urine	KS	*Escherichia coli*	21	21	carbapenemase not detected	>16	>32	<=1	<=4	<=2	>32	<=0.5	<=1	<=1	<=2
17210	Urine	KS	*Escherichia coli*	22	22	carbapenemase not detected	>16	>32	>16	<=4	>8	>32	<=0.5	<=1	<=1	<=2
17209	Urine	KS	*Escherichia coli*	20	20	carbapenemase not detected	>16	>32	>16	<=4	>8	>32	1	<=1	<=1	<=2
**17208**	Urine	KS	*Enterobacter cloacae*	21	21	carbapenemase not detected	>16	>32	16	<=4	<=2	>32	<=0.5	<=1	<=1	<=2
17207	Urine	KS	*Escherichia coli*	21	21	carbapenemase not detected	>16	>32	>16	<=4	<=2	>32	<=0.5	<=1	<=1	<=2
17206	Blood	CA	*Citrobacter freundii*	22	22	carbapenemase not detected	<=2	8	8	<=4	N/R	8	<=0.5	<=1	<=1	<=2
17205	Urine	CA	*Escherichia coli*	22	22	carbapenemase not detected	>16	>32	>16	<=4	<=2	>32	<=0.5	<=1	<=1	<=2
17204	Urine	CA	*Escherichia coli*	22	22	carbapenemase not detected	>16	>32	16	<=4	<=2	>32	<=0.5	<=1	<=1	<=2
17202	Blood	CA	*Escherichia coli*	22	23	carbapenemase not detected	16	>32	<=1	<=4	<=2	>32	<=0.5	<=1	<=1	<=2
17201	Blood	CA	*Escherichia coli*	6	6	serine carbapenemase detected	<=2	<=2	<=1	<=4	<=2	<=1	<=0.5	2	<=1	<=2
**17200**	Blood	CA	*Klebsiella pneumoniae*	6	6	serine carbapenemase detected	>16	>32	>16	<=4	>8	>32	>1	>8	>8	<=2
17199	Blood	CA	*Serratia marcescens*	6	6	serine carbapenemase detected	<=2	8	16	<=4	<=2	<=1	>1	>8	>8	<=2
**17198**	Blood	CA	*Escherichia coli*	6	20	metallo-beta-lactamase detected	>16	>32	>16	>16	>8	>32	>1	>8	>8	>16
17747	Blood	TN	*Acinetobacter baumannii*	20	20	carbapenemase not detected	>16	>32	>16	>16	8	>32	>1	>8	>8	16

Table S1: Phenotypic Results

Cepheid ID	Sample type	State	Organism by K-mer spectra	mCIM (mm)	eCIM (mm)	mCIM/eCIM Result	FEP MIC	CTX MIC (µg/ml)	CAZ MIC (µg/ml)	CZA MIC (µg/ml)	CT MIC (µg/ml)	CRO MIC (µg/ml)	ETP MIC (µg/ml)	IPM MIC (µg/ml)	MEM MIC (µg/ml)	MEV MIC (µg/ml)
17748	Blood	TN	*Acinetobacter baumannii*	21	22	carbapenemase not detected	16	16	8	>16	8	32	>1	>8	>8	16
**17195**	Blood	CA	*Pseudomonas aeruginosa*	6	24	metallo-beta-lactamase detected	>16	>32	>16	>16	>8	>32	>1	>8	>8	N/R
17194	Blood	CA	*Escherichia coli*	22	22	carbapenemase not detected	>16	>32	16	<=4	<=2	>32	<=0.5	<=1	<=1	<=2
**17193**	Blood	CA	*Enterobacter hormaechei* [illegible]	22	22	carbapenemase not detected	4	>32	>16	<=4	>8	>32	<=0.5	<=1	<=1	<=2
17192	Blood	CA	*Escherichia coli*	21	21	carbapenemase not detected	>16	>32	16	<=4	<=2	>32	<=0.5	<=1	<=1	<=2
**17191**	Blood	CA	*Klebsiella pneumoniae*	21	21	carbapenemase not detected	>16	>32	>16	<=4	<=2	>32	<=0.5	<=1	<=1	<=2
17190	Blood	CA	*Pseudomonas aeruginosa*	23	23	carbapenemase not detected	8	>32	8	8	<=2	>32	>1	>8	>8	N/R
17189	Blood	CA	*Pseudomonas aeruginosa*	22	22	carbapenemase not detected	16	>32	16	8	<=2	>32	>1	>8	>8	N/R
17188	Urine	CA	*Klebsiella aerogenes*	21	21	carbapenemase not detected	<=2	8	8	<=4	<=2	8	<=0.5	<=1	<=1	<=2
17187	Urine	CA	*Escherichia coli*	22	22	carbapenemase not detected	16	>32	4	<=4	<=2	>32	<=0.5	<=1	<=1	<=2
17186	Urine	CA	*Escherichia coli*	22	22	carbapenemase not detected	>16	>32	>16	<=4	4	>32	<=0.5	2	<=1	<=2
**17185**	Urine	CA	*Klebsiella pneumoniae*	6(inner colonies)	6 (inner colonies)	serine carbapenemase detected	>16	>32	>16	<=4	>8	>32	>1	>8	>8	16
**17184**	Urine	CA	*Escherichia coli*	6	21	metallo-beta-lactamase detected	>16	>32	>16	>16	>8	>32	>1	>8	>8	>16
17183	Urine	CA	*Escherichia coli*	6	6	serine carbapenemase detected	<=2	32	4	<=4	<=2	>32	>1	>8	<=1	<=2
17182	Urine	CA	*Klebsiella michiganensis*	6	19	metallo-beta-lactamase detected	>16	>32	>16	>16	>8	>32	>1	>8	>8	>16
**17181**	Urine	CA	*Enterobacter asburiae*	6	6	serine carbapenemase detected	>16	>32	>16	<=4	>8	>32	>1	8	8	<=2
**17180**	Urine	CA	*Enterobacter hormaechei*	6	6	serine carbapenemase detected	8	32	>16	16	>8	>32	>1	>8	8	<=2
**17179**	Urine	CA	*Klebsiella pneumoniae*	6	21	metallo-beta-lactamase detected	>16	>32	>16	>16	>8	>32	>1	>8	>8	>16
17178	Urine	CA	*Escherichia coli*	6	6	serine carbapenemase detected	>16	>32	>16	<=4	<=2	>32	>1	2	<=1	<=2
17836	Blood	IL	*Acinetobacter baumannii*	21	21	carbapenemase not detected	16	>32	>16	16	8	>32	>1	>8	>8	>16
17176	Urine	CA	*Pseudomonas aeruginosa*	6	6	serine carbapenemase detected	>16	>32	>16	>16	>8	>32	>1	>8	>8	N/R

Table S1: Phenotypic Results

Cepheid ID	Sample type	State	Organism by K-mer spectra	mCIM (mm)	eCIM (mm)	mCIM/eCIM Result	FEP MIC	CTX MIC (µg/ml)	CAZ MIC (µg/ml)	CZA MIC (µg/ml)	CT MIC (µg/ml)	CRO MIC (µg/ml)	ETP MIC (µg/ml)	IPM MIC (µg/ml)	MEM MIC (µg/ml)	MEV MIC (µg/ml)
17175	Urine	KS	*Escherichia coli*	23	23	carbapenemase not detected	>16	>32	4	<=4	<=2	>32	<=0.5	<=1	<=1	<=2
17174	Urine	KS	*Pseudomonas aeruginosa*	24 (1 col)	24	carbapenemase not detected	>16	>32	8	8	<=2	>32	>1	>8	>8	N/R
**17173**	Urine	KS	*Klebsiella pneumoniae*	22	22	carbapenemase not detected	>16	>32	>16	<=4	<=2	>32	<=0.5	<=1	<=1	<=2
17172	Urine	KS	*Escherichia coli*	23	23	carbapenemase not detected	>16	>32	16	<=4	<=2	>32	<=0.5	<=1	<=1	<=2

FEP: cefepime; CTX: cefotaxime; CAZ: ceftazidime; CZA: ceftazidime/avibactam; CT: ceftolozane/tazobactam; ETP: ertapenem; IPM: imipenem; MEM: meropenem; MEV: meropenem/tazobactam.
