# Supplementary material for: Characterization of Carbapenemase- and ESBL-Producing Gram-Negative Bacilli Isolated from Patients with Urinary Tract and Bloodstream Infections
Source: Antibiotics (Basel). 2023 Aug 30;12(9):1386. doi: 10.3390/antibiotics12091386 (PMC10525328; doi:10.3390/antibiotics12091386)
Supplement: Supplementary file 1 [file antibiotics-12-01386-s001.zip › Table S2.pdf]

Table S2. Genotypic Results

Cepheid ID	Sample type	Year	State	Organism by K-mer spectra	MLST	Resistance genes identified
17978	Blood	2022	GA	*Escherichia coli*	ST976	formA
17977	Blood	2022	GA	*Klebsiella pneumoniae*	ST1199	aac(6')-Ib,aadA2,aph(3')-Ia,blaKPC-3,blaSHV-187,catA1,dfrA12,fosA6,mph(A),OqxA,OqxB,qacE,sul1
17976	Urine	2022	GA	*Klebsiella pneumoniae*	ST16	aac(3)-IIa,aac(6')-Ib,aac(6')-Ib-cr,aadA13,ant(3'')-Ia,aph(3')-Ia,aph(3'')-Ib,aph(6)-Id,blaCTX-M-15,blaOXA-1,blaOXA-181,blaSHV-187,blaTEM-1B,catB3,dfrA14,floR,fosA5,mph(A),OqxA,OqxB,qacL,sul2,sul3
17975	Blood	2022	GA	*Klebsiella oxytoca*	ST2	blaOXY-2-8,fosA,OqxA,OqxB
17974	Blood	2022	GA	*Klebsiella pneumoniae*	ST13	blaCTX-M-15,blaKPC-3,blaSHV-187,blaTEM-1A,dfrA14,fosA6,OqxA,OqxB,qnrB1
17973	Urine	2022	GA	*Klebsiella pneumoniae*	ST258	aac(6')-Ib,aadA2,aph(3')-Ia,blaKPC-3,blaSHV-12,catA1,dfrA12,fosA6,mph(A),OqxA,OqxB,qacE,sul1
**17972**	Urine	2022	GA	*Escherichia coli*	ST33	aac(3)-IId,aac(6')-Ib-cr,aadA16,aph(3')-Ia,aph(3'')-Ib,aph(6)-Id,ARR-3,blaNDM-1,catB3,dfrA27,floR,mph(A),qacE,sitABCD,sul1,sul2,tet(A)
17971	Urine	2022	GA	*Escherichia coli*	ST43	blaKPC-3,formA,sitABCD
17970	Blood	2022	GA	*Klebsiella michiganensis*	ST311	aadA6,ant(2'')-Ia,blaKPC-3,blaOXA-2,blaOXY-4-1,blaSHV-187,fosA,OqxA,OqxB,qacE,sul1
17899	Blood	2022	MO	*Klebsiella pneumoniae*	ST11	aac(3)-IId,aac(6')-Ib-cr,aph(3'')-Ib,aph(6)-Id,blaCTX-M-15,blaOXA-1,blaSHV-187,blaTEM-1B,catA2,catB3,dfrA14,fosA6,OqxA,OqxB,qnrB1,sul2,tet(D)
17898	Blood	2022	MO	*Escherichia coli*	Inconclusive	aadA5,blaCTX-M-15,dfrA7,erm(B),formA,mph(A),qacE,qepA1,sitABCD,sul1
17897	Blood	2022	MO	*Escherichia coli*	ST3	blaDHA-1,blaTEM-1B,dfrA7,formA,mph(A),qacE,qnrB4,sitABCD,sul1

Table S2. Genotypic Results

Cepheid ID	Sample type	Year	State	Organism by K-mer spectra	MLST	Resistance genes identified
17896	Urine	2022	MO	*Escherichia coli*	ST3	aadA13,ant(3'')-Ia,blaCTX-M-15,dfrA1,formA,qacE,sitABCD,sul1
17895	Urine	2022	MO	*Escherichia coli*	Not applicable	aac(3)-IId,aadA5,blaCTX-M-15,blaTEM-1B,dfrA7,formA,qacE,sitABCD,sul1
17894	Urine	2022	MO	*Escherichia coli*	ST700	aadA13,ant(3'')-Ia,blaCTX-M-15,dfrA1,formA,sitABCD
17893	Urine	2022	MO	*Shigella flexneri*	ST2	aac(3)-IIa,aadA5,ant(3'')-Ia,aph(3')-Ia,aph(3'')-Ib,aph(6)-Id,blaCTX-M-55,blaTEM-1B,catA1,floR,fosA3,qacL,sitABCD,sul2,sul3,tet(A),tet(B)
17891	Blood	2022	KS	*Escherichia coli*	ST2	aadA5,aph(3'')-Ib,aph(6)-Id,blaCTX-M-14,blaTEM-1B,dfrA7,formA,qacE,sul2,tet(A)
17870	Blood	2022	WI	*Escherichia coli*	ST8	aph(3'')-Ib,aph(6)-Id,blaCTX-M-15,blaTEM-1B,catA1,dfrA7,mdf(A),qacE,sitABCD,sul1,sul2,tet(D)
17869	Blood	2022	WI	*Klebsiella aerogenes*	Inconclusive	blaACT-4,formA,fosA,mdf(A),OqxA,OqxB,sitABCD,tet(34)
17868	Blood	2022	WI	*Escherichia coli*	ST43	aadA5,blaCTX-M-15,dfrA17,mdf(A),mph(A),qacE,sitABCD,sul1
17867	Blood	2022	WI	*Raoultella ornithinolytica*	Not applicable	aac(3)-IIa,aac(6')-Ib-cr,aph(3'')-Ib,aph(6)-Id,blaCTX-M-15,blaOXA-1,blaPLA1a,blaTEM-1B,catB3,dfrA14,fosA,qnrB1,sul2,tet(A)
17866	Blood	2022	WI	*Escherichia coli*	ST53	blaCTX-M-15,mdf(A),sitABCD
17865	Blood	2022	WI	*Klebsiella pneumoniae*	ST45	aac(3)-IIa,aac(6')-Ib-cr,aph(3'')-Ib,aph(6)-Id,blaCTX-M-15,blaOXA-1,blaSHV-187,blaTEM-1B,catB3,dfrA14,fosA,OqxA,OqxB,qnrB1,sul2,tet(A)
17860	Blood	2022	WI	*Escherichia coli*	ST8	aadA5,aph(3'')-Ib,aph(6)-Id,blaCTX-M-27,dfrA17,mdf(A),mph(A),qacE,sul1,sul2,tet(A)

Table S2. Genotypic Results

Cepheid ID	Sample type	Year	State	Organism by K-mer spectra	MLST	Resistance genes identified
17859	Blood	2022	WI	*Pseudomonas aeruginosa*	ST244	aph(3')-IIb,blaOXA-396,blaPDC-423,catB7,crpP,fosA
17858	Blood	2022	WI	*Escherichia coli*	ST2	aac(3)-IIa,aadA5,aph(3'')-Ib,aph(6)-Id,blaCTX-M-15,dfrA17,mdf(A),mph(A),sitABCD,sul2,tet(B)
17857	Blood	2022	WI	*Escherichia coli*	ST43	aac(3)-IIa,aac(6')-Ib-cr,ant(2'')-Ia,ant(3'')-Ia,blaCTX-M-15,blaOXA-1,catB3,cml,cmlA1,mdf(A),mph(A),qacE,sitABCD,sul1,tet(A)
17856	Blood	2022	WI	*Escherichia coli*	ST43	aac(3)-IIa,aac(6')-Ib-cr,aadA5,blaCTX-M-15,blaOXA-1,catB3,dfrA17,mdf(A),mph(A),qacE,sitABCD,sul1,tet(A)
17855	Blood	2022	WI	*Klebsiella pneumoniae*	ST1452	aac(3)-IId,aac(6')-Ib-cr,aadA16,aph(3')-Ia,aph(3'')-Ib,aph(6)-Id,ARR-3,blaCTX-M-3,blaSHV-187,blaTEM-1B,dfrA27,floR,fosA6,mph(A),OqxA,OqxB,qacE,qnrS1,sul1,sul2,tet(A)
17854	Blood	2022	WI	*Escherichia coli*	ST3	aph(3'')-Ib,aph(6)-Id,blaCTX-M-15,blaTEM-1B,dfrA14,mdf(A),qnrS1,sitABCD,sul2,tet(A)
17853	Blood	2022	WI	*Klebsiella michiganensis*	ST85	blaKPC-2,blaOXY-1-7,blaTEM-1B
17852	Blood	2022	WI	*Klebsiella pneumoniae*	ST258	aac(6')-Ib,ant(3'')-Ia,aph(3'')-Ib,aph(6)-Id,blaKPC-3,blaOXA-9,blaSHV-187,catA1,dfrA14,fosA6,OqxA,OqxB,sul2
17851	Urine	2022	WI	*Klebsiella pneumoniae*	ST22	aac(3)-IIa,aac(6')-Ib-cr,aph(3'')-Ib,aph(6)-Id,blaCTX-M-15,blaOXA-1,blaSHV-187,blaTEM-1B,catB3,dfrA14,fosA6,OqxA,OqxB,qnrB1,sul2,tet(A)
17850	Urine	2022	WI	*Escherichia coli*	ST8	aac(3)-IIa,blaCTX-M-14,blaTEM-1B,mdf(A),tet(D)
17849	Urine	2022	WI	*Escherichia coli*	ST43	aac(3)-IId,aac(6')-Ib-cr,aadA16,aadA2,ARR-3,blaCTX-M-27,blaTEM-1B,dfrA12,dfrA27,mdf(A),qacE,sitABCD,sul1,tet(A)
17848	Urine	2022	WI	*Escherichia coli*	ST43	aac(3)-IIa,aac(6')-Ib-cr,blaCTX-M-15,blaOXA-1,catB3,mdf(A),sitABCD

Table S2. Genotypic Results

Cepheid ID	Sample type	Year	State	Organism by K-mer spectra	MLST	Resistance genes identified
17847	Urine	2022	WI	*Escherichia coli*	Inconclusive	aac(3)-IId,ant(3'')-Ia,blaCMY-2,blaTEM-1B,mdf(A),sitABCD,tet(A)
**17846**	Urine	2022	WI	*Enterobacter sp.*	Inconclusive	ant(2'')-Ia,blaACT-16,blaSHV-187,fosA,qnrS1
17845	Urine	2022	WI	*Klebsiella pneumoniae*	ST2004-like	aac(3)-IIa,aph(3'')-Ib,aph(6)-Id,blaCTX-M-15,blaSHV-187,blaTEM-1B,dfrA14,fosA6,OqxA,OqxB,qnrB1,sul2,tet(A)
**17844**	Urine	2022	WI	*Enterobacter sp.*	Inconclusive	ant(2'')-Ia,blaACT-16,blaSHV-187,fosA,qnrS1
17843	Urine	2022	WI	*Providencia stuartii*	Not available	aac(2')-Ia,catA3,tet(B)
17842	Urine	2022	WI	*Klebsiella pneumoniae*	ST2546-like	aac(3)-IIa,aac(6')-Ib-cr,aph(3'')-Ib,aph(6)-Id,blaCTX-M-15,blaOXA-1,blaSHV-187,blaTEM-1B,catB3,dfrA14,fosA,OqxA,OqxB,qnrB1,sul2,tet(A)
17841	Urine	2022	WI	*Citrobacter freundii*	Inconclusive	aac(6')-Ib-cr,aph(3')-Ia,aph(3'')-Ib,aph(6)-Id,blaCMY-108,blaKPC-2,blaSHV-12,blaTEM-1B,dfrA19,mcr-9,qnrB2,qnrB6,sul1
17840	Urine	2022	WI	*Klebsiella pneumoniae*	ST348	aac(3)-IIa,aph(3'')-Ib,aph(6)-Id,blaCTX-M-15,blaSHV-187,blaTEM-1B,dfrA14,fosA,OqxA,OqxB,qnrB1,sul2
17839	Urine	2022	WI	*Escherichia coli*	ST2	aadA2,aph(3'')-Ib,aph(6)-Id,blaCTX-M-27,catA1,dfrA12,mdf(A),mph(A),qacE,sul1,sul2,tet(A)
17838	Urine	2022	WI	*Citrobacter freundii*	ST415	aac(3)-IId,aac(6')-Ib-cr,aadA2,aph(3'')-Ib,aph(6)-Id,blaCMY-48,blaKPC-2,blaSHV-12,blaTEM-1B,catA2,dfrA12,mcr-9,mph(A),qnrB2,sul1,sul2,tet(D)
17837	Urine	2022	WI	*Klebsiella pneumoniae*	ST258	aac(6')-Ib,ant(3'')-Ia,aph(3'')-Ib,aph(6)-Id,blaKPC-3,blaOXA-9,blaSHV-187,blaTEM-1A,catA1,dfrA14,fosA6,OqxA,OqxB,sul2
17177	Urine	2021	CA	*Acinetobacter baumannii*	ST2	aph(3')-Ia,aph(3'')-Ib,aph(6)-Id,armA,blaADC-25,blaOXA-23,blaOXA-66,blaTEM-1D,mph(E),msr(E),sul2,tet(B)

Table S2. Genotypic Results

Cepheid ID	Sample type	Year	State	Organism by K-mer spectra	MLST	Resistance genes identified
17835	Blood	2022	IL	*Serratia nematodiphila*	Not available	aac(6')-Ic,blaSRT-1,blaTEM-1A,qnrB19,qnrB5
17834	Blood	2022	IL	*Escherichia coli*	ST43	aac(3)-IIa,aac(6')-Ib-cr,ant(2'')-Ia,ant(3'')-Ia,blaCTX-M-15,blaOXA-1,catB3,cmlA1,mdf(A),mph(A),qacE,sitABCD,sul1
17833	Blood	2022	IL	*Klebsiella pneumoniae*	ST37	aac(3)-IIa,aac(6')-Ib-cr,aph(3'')-Ib,aph(6)-Id,blaCTX-M-15,blaOXA-1,blaSHV-187,blaTEM-1B,catB3,dfrA14,fosA6,OqxA,OqxB,qnrB1,sul2,tet(A)
17832	Blood	2022	IL	*Klebsiella pneumoniae*	ST152	aac(6')-Ib-cr,aadA16,ARR-3,blaCTX-M-15,blaKPC-3,blaSHV-187,blaTEM-1A,dfrA27,fosA,OqxA,OqxB,qacE,qnrB6,sul1
17831	Blood	2022	IL	*Escherichia coli*	ST43	aac(3)-IId,aadA2,aadA5,aph(3'')-Ib,aph(6)-Id,blaCTX-M-15,blaTEM-1B,dfrA12,dfrA17,mdf(A),mph(A),qacE,qnrB19,sitABCD,sul1,sul2,tet(A)
17830	Blood	2022	IL	*Escherichia coli*	ST3	aph(3'')-Ib,aph(6)-Id,blaCTX-M-27,blaTEM-1B,dfrA7,mdf(A),qacE,sitABCD,sul1,sul2,tet(A)
17829	Blood	2022	IL	*Proteus mirabilis*	Not available	aadA2,ant(2'')-Ia,ant(3'')-Ia,aph(6)-Id,blaCTX-M-14,blaTEM-1B,cat,dfrA1,qacE,sul1,tet(J)
17828	Blood	2022	IL	*Escherichia coli*	ST43	aac(3)-IIa,aac(6')-Ib-cr,blaCTX-M-15,blaOXA-1,catB3,mdf(A),sitABCD,tet(A)
17827	Blood	2022	IL	*Escherichia coli*	ST43	blaCTX-M-15,mdf(A),sitABCD,tet(A)
17826	Blood	2022	IL	*Escherichia coli*	ST9	aadA5,blaCTX-M-27,blaTEM-1B (trunc),dfrA17,mdf(A),mph(A),qacE,sul1
**17825**	Blood	2022	IL	*Enterobacter ludwigii*	ST1306	blaACT-12,fosA2
17824	Blood	2022	IL	*Escherichia coli*	ST43	aadA5,blaCTX-M-15,blaTEM-1B,dfrA17,mdf(A),mph(A),qacE,sitABCD,sul1

Table S2. Genotypic Results

Cepheid ID	Sample type	Year	State	Organism by K-mer spectra	MLST	Resistance genes identified
17823	Blood	2022	IL	*Escherichia coli*	Inconclusive	aac(3)-IId,aadA5,aph(3'')-Ib,aph(6)-Id,blaCTX-M-27,blaTEM-1B,dfrA17,mdf(A),mph(A),qacE,sitABCD,sul1,sul2,tet(A)
17822	Blood	2022	IL	*Escherichia coli*	ST43	aac(6')-Ib-cr,aadA5,blaCTX-M-15,blaOXA-1,catB3,dfrA17,mph(A),qacE,sitABCD,sul1,tet(A)
17821	Urine	2022	IL	*Escherichia coli*	ST43	aadA5,blaCMY-2,dfrA17,mdf(A),qacE,sitABCD,sul1
17820	Urine	2022	IL	*Escherichia coli*	ST53	aac(3)-IIa,aac(6')-Ib-cr,aph(3'')-Ib,aph(6)-Id,blaCTX-M-15,blaOXA-1,catB3,mdf(A),sitABCD,sul2,tet(B)
17819	Urine	2022	IL	*Escherichia coli*	ST43	aac(3)-IIa,aac(6')-Ib-cr,blaCTX-M-15,blaOXA-1,catB3,mdf(A),sitABCD,tet(A)
17818	Urine	2022	IL	*Escherichia coli*	ST43	aac(3)-IIa,aac(6')-Ib-cr,blaCTX-M-15,blaOXA-1,catB3,mdf(A),sitABCD
17817	Urine	2022	IL	*Escherichia coli*	ST2	aph(3')-Ia,aph(3'')-Ib,aph(6)-Id,blaCTX-M-55,catA1,mdf(A),sitABCD,sul2,tet(A),tet(B)
**17816**	Urine	2022	IL	*Enterobacter hormaechei*	Inconclusive	aph(3'')-Ib,aph(6)-Id,blaACT-16,blaTEM-1B,dfrA14,fosA,sul2,tet(D)
17815	Urine	2022	IL	*Citrobacter freundii*	Inconclusive	aac(6')-If,aadA2,blaCMY-150,dfrA12,mph(A),qacE,qnrB38,sul1,tet(A)
17814	Urine	2022	IL	*Escherichia coli*	ST901	aadA5,aph(3'')-Ib,aph(6)-Id,blaCTX-M-55,dfrA17,floR,mdf(A),sul2,tet(A)
17813	Urine	2022	IL	*Escherichia coli*	Inconclusive	aadA2,blaCTX-M-14,dfrA12,mdf(A),mph(A),qacE,sitABCD,sul1
**17812**	Urine	2022	IL	*Klebsiella pneumoniae*	ST37	aac(3)-IIa,aph(6)-Id,blaCTX-M-15,blaSHV-187,blaTEM-1B (truncated),catB3,fosA6,OqxA,OqxB,qnrB1,sul2,tet(A)

Table S2. Genotypic Results

Cepheid ID	Sample type	Year	State	Organism by K-mer spectra	MLST	Resistance genes identified
17811	Urine	2022	IL	*Escherichia coli*	Inconclusive	aac(6')-Ib-cr,blaCTX-M-15,catB3,mdf(A),sitABCD
17810	Urine	2022	IL	*Escherichia coli*	ST43	aac(3)-IId,aadA2,aadA5,blaCTX-M-15,blaTEM-1B,dfrA12,dfrA17,mdf(A),mph(A),qacE,qnrB19,sitABCD,sul1
17809	Urine	2022	IL	*Escherichia coli*	ST43	aac(6')-Ib-cr,ant(2'')-Ia,ant(3'')-Ia,blaCTX-M-15,blaOXA-1,catB3,cmlA1,mdf(A),mph(A),qacE,qnrB19,sitABCD,sul1,tet(A)
17808	Urine	2022	IL	*Escherichia coli*	ST43	aac(3)-IIa,aac(6')-Ib-cr,blaCTX-M-15,blaOXA-1,catB3,mdf(A),sitABCD
17807	Urine	2022	IL	*Providencia stuartii*	Not available	aac(2')-Ia,blaTEM-106,catA3,tet(B)
17755	Blood	2021	TN	*Escherichia coli*	ST43	aac(3)-IId,aph(3'')-Ib,blaCTX-M-14,blaTEM-1B,mdf(A),mph(A),qacE,sitABCD,sul1,sul2
17754	Blood	2021	TN	*Pseudomonas aeruginosa*	ST1158	aph(3')-IIb,blaOXA-395,blaPDC-476,catB7,crpP,fosA
17753	Blood	2021	TN	*Citrobacter freundii*	ST98	aac(6')-Ib,ant(3'')-Ia,blaCMY-109,blaKPC-2,blaOXA-9,blaTEM-1A,qnrB38
17752	Blood	2021	TN	*Klebsiella pneumoniae*	ST111	aadA2,blaCTX-M-3,blaSHV-187,blaTEM-1B,dfrA21,fosA6,OqxA,OqxB,qacE,sul1,tet(D)
17751	Blood	2021	TN	*Escherichia coli*	ST43	aadA5,aph(3'')-Ib,aph(6)-Id,blaCTX-M-27,dfrA17,mdf(A),mph(A),qacE,sitABCD,sul1,sul2,tet(A)
17750	Blood	2021	TN	*Escherichia coli*	ST43	aac(3)-IIa,aac(6')-Ib-cr,aadA2,ant(3'')-Ia,blaCARB-2,blaCTX-M-15,blaOXA-1,catB3,cmlA1,dfrA12,mdf(A),qacE,qnrA1,sul1,sul3,tet(A)
17749	Blood	2021	TN	*Pseudomonas aeruginosa*	ST446	aph(3')-IIa,aph(3')-IIb,aph(6)-Ic,blaOXA-395,blaPDC-16,catB7,crpP,fosA

Table S2. Genotypic Results

Cepheid ID	Sample type	Year	State	Organism by K-mer spectra	MLST	Resistance genes identified
17196	Blood	2021	CA	*Acinetobacter baumannii*	ST2	aph(3'')-Ib,aph(6)-Id,blaADC-25,blaOXA-237,blaOXA-66,sul2,tet(B)
17197	Blood	2021	CA	*Acinetobacter baumannii*	ST2	aph(3'')-Ib,aph(6)-Id,blaADC-25,blaOXA-237,blaOXA-66,sul2,tet(B)
17746	Blood	2021	TN	*Pseudomonas aeruginosa*	ST319	aph(3')-IIb,blaOXA-488,blaPDC-46,catB7,fosA
17745	Blood	2021	TN	*Pseudomonas aeruginosa*	Inconclusive	aph(3')-IIb,blaOXA-396,blaPDC-5,catB7,fosA
17744	Blood	2021	TN	*Pseudomonas juntendi*	Inconclusive	blaPDC-184 (68%)
17743	Blood	2021	TN	*Klebsiella pneumoniae*	ST405	aac(3)-IIa,aac(6')-Ib-cr,aac(6')-Il,ant(3'')-Ia,aph(3'')-Ib,aph(6)-Id,blaCTX-M-15,blaOXA-1,blaSHV-76,blaTEM-1B,blaVIM-1,catB3,dfrA1,dfrA14,fosA,mph(A),OqxA,OqxB,qnrS1,sul2
17742	Blood	2021	TN	*Serratia marcescens*	Not available	aac(6')-Ic,aac(6')-Il,ant(3'')-Ia,blaSRT-1,blaTEM-1B,blaVIM-1,dfrA1,mph(A)
17741	Blood	2021	TN	*Klebsiella pneumoniae*	ST405	aac(3)-IIa,aac(6')-Ib-cr,aac(6')-Il,ant(3'')-Ia,aph(3'')-Ib,aph(6)-Id,blaCTX-M-15,blaOXA-1,blaSHV-187,blaSHV-76,blaTEM-1B,blaVIM-1,catB3,dfrA1,dfrA14,fosA,mph(A),OqxA,OqxB,qnrS1,sul2
17740	Urine	2021	TN	*Klebsiella pneumoniae*	ST307	aac(3)-IIa,aac(6')-Ib-cr,aph(3'')-Ib,aph(6)-Id,blaCTX-M-15,blaOXA-1,blaSHV-106/28,catB3,dfrA14,fosA6,OqxA,OqxB,qnrB1,sul2,tet(A)
17739	Urine	2021	TN	*Klebsiella aerogenes*	Not available	blaCMY-63 (75%),sitABCD,OqxA,OqxB,formA,fosA7,fosA,mdf(A),tet(34)
**17738**	Urine	2021	TN	*Enterobacter hormaechei*	ST461	aac(3)-IId,aadA2,blaACT-7,blaTEM-1B,catA2,dfrA12,fosA,mph(A),qacE,sul1,sul2,tet(D)
17737	Urine	2021	TN	*Proteus mirabilis*	Not available	ant(3'')-Ia,armA,blaCARB-2,blaCTX-M-15,blaFOX-5,blaTEM-1B,catA1,dfrA1,mph(E),msr(E),qacE,sul1,tet(J)

Table S2. Genotypic Results

Cepheid ID	Sample type	Year	State	Organism by K-mer spectra	MLST	Resistance genes identified
17736	Urine	2021	TN	*Klebsiella pneumoniae*	ST37	aac(6')-Ib-cr,aac(6')-Ib-cr,blaCTX-M-15,blaOXA-1,blaSHV-187,catB3,dfrA14,fosA6,OqxA,OqxB,qnrB1,tet(A)
17735	Urine	2021	TN	*Klebsiella pneumoniae*	ST219	aadA2,aph(3'')-Ib,aph(6)-Id,blaCTX-M-15,blaSHV-187,dfrA12,fosA,mph(A),OqxA,OqxB,qacE,qnrS1,sul1,sul2
17734	Urine	2021	TN	*Klebsiella pneumoniae*	ST2217	aac(6')-Ib-cr,aph(3'')-Ib,aph(6)-Id,blaCTX-M-15,blaOXA-1,blaSHV-187,blaTEM-1B,catB3,dfrA14,fosA6,OqxA,OqxB,qnrB1,sul2,tet(A)
17733	Urine	2021	TN	*Escherichia coli*	ST43	aadA5,aph(3'')-Ib,aph(6)-Id,blaCTX-M-15,dfrA17,mdf(A),mph(A),qacE,sitABCD,sul1,sul2,tet(A)
17732	Urine	2021	TN	*Klebsiella pneumoniae*	ST268	aadA2,aph(3')-Ia,aph(3'')-Ib,aph(6)-Id,blaCTX-M-15,blaSHV-187,catA2,dfrA12,fosA5,mph(A),OqxA,OqxB,qacE,qnrS1,sul1,sul2
17731	Urine	2021	TN	*Escherichia coli*	ST700	ant(3'')-Ia,aph(3'')-Ib,aph(6)-Id,blaCTX-M-15,blaTEM-1A,dfrA1,mdf(A),sitABCD,sul2,tet(A)
17730	Urine	2021	TN	*Escherichia coli*	ST43	blaCTX-M-15,blaTEM-1B,mdf(A),sitABCD
17729	Urine	2021	TN	*Klebsiella pneumoniae*	ST4452	aac(6')-Ib-cr,aph(3'')-Ib,aph(6)-Id,blaCTX-M-15,blaOXA-1,blaSHV-187,blaTEM-1B,catB3,dfrA14,fosA6,fosA7,OqxA,OqxB,qnrB1,sul2,tet(A)
17728	Urine	2021	TN	*Escherichia coli*	ST3	blaCTX-M-15,dfrA14,erm(B),mdf(A),mph(A),sitABCD,tet(B)
17727	Urine	2021	TN	*Escherichia coli*	ST43	aadA5,aph(3'')-Ib,aph(6)-Id,blaCTX-M-27,dfrA17,mdf(A),mph(A),qacE,sitABCD,sul1,sul2,tet(A)
17726	Urine	2021	TN	*Klebsiella pneumoniae*	ST405	aac(3)-IIa,aac(6')-Ib-cr,aac(6')-II,ant(2'')-Ia,ant(3'')-Ia,aph(3'')-Ib,aph(6)-Id,blaCTX-M-15,blaOXA-1,blaSHV-76,blaTEM-1B,blaVIM-1,catB3,dfrA1,dfrA14,fosA,mph(A),OqxA,OqxB,qnrS1,sul2
17722	Blood	2021	CA	*Escherichia coli*	ST43	aadA5,aph(3'')-Ib,aph(6)-Id,blaCTX-M-27,dfrA17,mdf(A),mph(A),qacE,sitABCD,sul1,sul2,tet(A)

Table S2. Genotypic Results

Cepheid ID	Sample type	Year	State	Organism by K-mer spectra	MLST	Resistance genes identified
17721	Blood	2021	CA	*Pseudomonas aeruginosa*	ST633	aph(3')-IIb,blaOXA-396,blaPDC-171,catB7,crpP,fosA
17720	Blood	2021	CA	*Pseudomonas aeruginosa*	ST633	aph(3')-IIb,blaOXA-396,blaPDC-171,catB7,crpP,fosA
17719	Blood	2021	CA	*Pseudomonas aeruginosa*	ST235	aadA6,aph(3')-IIb,blaOXA-488,blaPDC-35,catB7,fosA,qacE,sul1
17718	Blood	2021	CA	*Pseudomonas aeruginosa*	ST167	aac(6')-Ib,aac(6')-II,aadA6,ant(3'')-Ia,aph(3')-IIb,aph(3')-VIa,blaIMP-15,blaOXA-2,blaOXA-396,blaPDC-445,catA1,catB7,fosA,qacE,sul1
17717	Blood	2021	CA	*Pseudomonas aeruginosa*	ST244	aph(3')-IIb,blaOXA-396,blaOXA-494,blaPDC-423,catB7,fosA
17716	Urine	2021	CA	*Klebsiella pneumoniae*	ST4145-like	aph(3'')-Ib,aph(6)-Id,blaCTX-M-15,blaSHV-187,blaTEM-1B,dfrA14,fosA5,OqxA,OqxB,qnrB1,sul2
17715	Urine	2021	CA	*Klebsiella pneumoniae*	ST2546-like	aph(3'')-Ib,aph(6)-Id,blaCTX-M-15,blaSHV-187,blaTEM-1B,dfrA14,fosA,OqxA,OqxB,qnrB1,sul2,tet(A)
**17714**	Urine	2021	CA	*Enterobacter hormaechei*	ST45	aadA2,blaACT-15,mcr-10,qacE,sul1
17713	Urine	2021	CA	*Escherichia coli*	Inconclusive	aadA5,aph(3'')-Ib,aph(6)-Id,blaTEM-1B,dfrA17,mdf(A),mph(A),qacE,sitABCD,sul1,sul2,tet(A)
17712	Urine	2021	CA	*Klebsiella pneumoniae*	ST4145-like	aph(3'')-Ib,aph(6)-Id,blaCTX-M-15,blaSHV-187,blaTEM-1B,dfrA14,fosA5,OqxA,OqxB,qnrB1,sul2
17316	Blood	2021	NJ	*Acinetobacter baumannii*	ST-235	aac(6')-Ib-cr,aph(3'')-Ib,aph(3')-VIa,aph(6)-Id,blaADC-25,blaOXA-71,blaSHV-12,sul2,tet(B)
17710	Urine	2021	CA	*Pseudomonas aeruginosa*	Inconclusive	aph(3')-IIb,blaOXA-395,blaPDC-115,catB7,fosA

Table S2. Genotypic Results

Cepheid ID	Sample type	Year	State	Organism by K-mer spectra	MLST	Resistance genes identified
17709	Urine	2021	CA	*Pseudomonas aeruginosa*	ST852	aph(3')-IIb,blaOXA-396,blaPDC-45,catB7,crpP,fosA
17708	Urine	2021	CA	*Klebsiella aerogenes*	ST93	blaCMY-74 (74%),formA,fosA,mdf(A),OqxA,OqxB,sitABCD,tet(34)
17707	Urine	2021	CA	*Klebsiella pneumoniae*	ST307	aac(3)-IIa,aac(6')-Ib-cr,aac(6')-Ib-cr,aph(3'')-Ib,aph(6)-Id,blaCTX-M-15,blaOXA-1,blaSHV-106,blaTEM-1B,catB3,dfrA14,fosA6,OqxA,OqxB,qnrB1,sul2,tet(A)
17706	Urine	2021	CA	*Klebsiella pneumoniae*	ST258	aac(6')-Ib,aadA1,aadA2,blaKPC-3,blaOXA-9,blaSHV-187,blaTEM-168 (trunc),catA1,dfrA12,dfrA14,fosA6,OqxA,OqxB,qacE,sul1
17638	Blood	2021	NM	*Escherichia coli*	ST43	aadA5,aph(3'')-Ib,aph(6)-Id,blaCTX-M-27,dfrA7,formA,mph(A),qacE,sitABCD,sul1,sul2,tet(A)
17637	Blood	2021	NM	*Escherichia coli*	ST43	aac(3)-IIa,aac(6')-Ib-cr,blaCTX-M-15,blaOXA-1,catB3,mdf(A),sitABCD,tet(A)
17636	Blood	2021	NM	*Pseudomonas aeruginosa*	ST111	aac(6')-Ib,aadA2,aph(3')-IIb,blaCARB-2,blaOXA-395,blaPDC-44,catB7,crpP,fosA,qacE,sul1
17324	Urine	2021	GA	*Acinetobacter baumannii*	ST2	aac(6')-Ian,ant(2'')-Ia,aph(3')-Ia,aph(3'')-Ib,aph(6)-Id,blaADC-25,blaOXA-23,blaOXA-834,sul2,tet(B)
17634	Blood	2021	NM	*Pseudomonas aeruginosa*	ST308	aac(6')-30-aac(6')-Ib',aadA6,ant(3'')-Ia,aph(3')-IIb,blaOXA-2,blaOXA-488,blaPDC-19a,catB7,crpP,fosA,qacE,sul1
17628	Blood	2021	OH	*Escherichia coli*	ST132	aadA5,aph(3'')-Ib,aph(6)-Id,blaCTX-M-15,blaTEM-1B,dfrA17,mdf(A),mph(A),qacE,sitABCD,sul1,sul2,tet(A)
**17627**	Blood	2021	OH	*Enterobacter bugandensis*	Not available	blaACT-6,catA1,formA,fosA,mdf(A),OqxA,OqxB,sitABCD,tet(34) (
17626	Blood	2021	OH	*Escherichia coli*	ST43	aph(3'')-Ib,aph(6)-Id,blaCTX-M-15,blaTEM-1B,dfrA14,mdf(A),sitABCD,sul2

Table S2. Genotypic Results

Cepheid ID	Sample type	Year	State	Organism by K-mer spectra	MLST	Resistance genes identified
17625	Blood	2021	OH	*Escherichia coli*	ST53	aac(3)-IIa,aac(6')-Ib-cr,aph(3'')-Ib,aph(6)-Id,blaCTX-M-15,blaOXA-1,catB3,dfrA17,mdf(A),sitABCD,sul2,tet(B)
17624	Blood	2021	OH	*Escherichia coli*	Inconclusive	blaCTX-M-14,mdf(A),sitABCD
**17623**	Blood	2021	OH	*Enterobacter hormaechei*	ST108	blaACT-7
17622	Blood	2021	OH	*Serratia marcescens*	Not possible	aac(6')-Ic,blaSRT-2,tet(41)
17621	Blood	2021	OH	*Serratia marcescens*	Not possible	aac(6')-Ic,blaSRT-2,tet(41)
17620	Blood	2021	OH	*Escherichia coli*	Inconclusive	aac(3)-IIa,aac(6')-Ib-cr,blaCTX-M-15,blaCTX-M-27,blaOXA-1,blaTEM-1B,catB3,mdf(A),mdf(A),sitABCD
17619	Blood	2021	OH	*Klebsiella pneumoniae*	ST4083	aadA2,aph(3')-Ia,aph(3'')-Ib,aph(6)-Id,blaCTX-M-15,blaSHV-187,dfrA12,fosA5,mph(A),OqxA,OqxB,qacE,qnrS1,sul1,sul2
**17618**	Blood	2021	OH	*Enterobacter hormaechei*	Inconclusive	blaACT-7
17617	Blood	2021	OH	*Escherichia coli*	Inconclusive	aadA5,aph(3'')-Ib,aph(6)-Id,blaCTX-M-27,dfrA17,mdf(A),mph(A),qacE,sitABCD,sul1,sul2,tet(A)
**17616**	Blood	2021	OH	*Enterobacter hormaechei supsp. xiangfangensis*	ST116	aac(6')-Ib-cr,aadA2,ant(3'')-Ia,aph(3'')-Ib,aph(6)-Id,blaACT-7,blaSHV-12,blaTEM-1B,catA2,dfrA19,fosA,mcr-9,qacE,sul1
17615	Blood	2021	OH	*Pseudomonas aeruginosa*	ST966	aac(3)-IIIb,aph(3')-IIb,blaOXA-395,blaPDC-471,catB7,crpP,fosA
17614	Blood	2021	OH	*Pseudomonas aeruginosa*	ST17	aph(3')-IIb,blaOXA-50,blaPDC-240,catB7,crpP,fosA

Table S2. Genotypic Results

Cepheid ID	Sample type	Year	State	Organism by K-mer spectra	MLST	Resistance genes identified
17613	Urine	2021	OH	*Escherichia coli*	Inconclusive	aph(6)-Id,blaTEM-1B,mdf(A),sitABCD,sul2
17612	Urine	2021	OH	*Escherichia coli*	ST43	aac(3)-IId,blaCTX-M-14,blaTEM-1B,mdf(A),sitABCD
17611	Urine	2021	OH	*Escherichia coli*	Inconclusive	blaCTX-M-32,blaTEM-1A,dfrA1,floR,mdf(A),mph(A),qacE,sul1,tet(A)
17610	Urine	2021	OH	*Escherichia coli*	ST53	aac(3)-IId,aadA5,aph(3'')-Ib,aph(6)-Id,blaCTX-M-15,blaTEM-1B,dfrA17,mdf(A),mph(A),qacE,sitABCD,sul1,sul2,tet(A)
**17609**	Urine	2021	OH	*Enterobacter hormaechei supsp. xiangfangensis*	Inconclusive	aac(3)-IIa,aac(6')-Ib-cr,aph(3'')-Ib,aph(6)-Id,blaACT-16,blaACT-7,blaCTX-M-15,blaOXA-1,blaTEM-1B,catB3,dfrA14,fosA,sul2,tet(A)
17608	Urine	2021	OH	*Citrobacter freundii*	ST169	aac(3)-IId,aadA2,blaCMY-65,blaTEM-1B,dfrA12,mph(A),qacE,qnrB38,sul1,sul2,tet(D)
17607	Urine	2021	OH	*Proteus mirabilis*	Not available	aac(6')-Ib-cr,aph(3')-Ia,aph(3'')-Ib,aph(6)-Id,blaCMY-2,sul2,tet(A),tet(J)
17606	Urine	2021	OH	*Citrobacter freundii*	ST97	blaCFE-1,qnrB27
17605	Urine	2021	OH	*Escherichia coli*	ST3	aadA5,aph(3'')-Ib,aph(6)-Id,blaTEM-1B,dfrA17,mdf(A),mph(A),qacE,sitABCD,sul1,sul2
17604	Urine	2021	OH	*Escherichia coli*	ST53	aac(3)-IIa,aac(6')-Ib-cr,blaCTX-M-15,blaOXA-1,catB3,mdf(A),sitABCD
**17603**	Urine	2021	OH	*Enterobacter hormaechei*	ST662	blaACT-7,fosA
17602	Urine	2021	OH	*Escherichia coli*	ST43	blaCTX-M-15,mdf(A),sitABCD

Table S2. Genotypic Results

Cepheid ID	Sample type	Year	State	Organism by K-mer spectra	MLST	Resistance genes identified
17601	Urine	2021	OH	*Escherichia coli*	Inconclusive	aph(3'')-Ib,aph(6)-Id,blaCTX-M-15,mdf(A),mph(A),qnrS1,sitABCD,sul2
**17600**	Urine	2021	OH	*Enterobacter hormaechei*	ST45	aph(3'')-Ib,aph(6)-Id,blaACT-15,blaTEM-1B,dfrA14,sul2
17599	Urine	2021	OH	*Proteus penneri*	Not available	hugA
17598	Blood	2021	WA	*Pseudomonas aeruginosa*	ST1244	aph(3')-IIb,blaOXA-50,blaPDC-442,catB7,crpP,fosA
**17597**	Blood	2021	WA	*Enterobacter cloacae complex*	ST93	blaACT-7,fosA
17596	Blood	2021	WA	*Proteus mirabilis*	Not available	aac(3)-IId,aac(6')-Ib-cr,aadA5,ant(3'')-Ia,aph(3')-Ia,aph(3'')-Ib,aph(6)-Id,ARR-3,blaCTX-M-65,blaOXA-1,blaTEM-1B,cat,catA1,catB3,dfrA1,dfrA17,qacE,sul1,sul2,tet(J)
17595	Blood	2021	WA	*Klebsiella pneumoniae*	ST25	aac(3)-IIa,aph(3'')-Ib,aph(6)-Id,blaCTX-M-15,blaSHV-187,blaTEM-1B,dfrA14,fosA6,OqxA,OqxB,qnrB1,sul2
17590	Blood	2021	MO	*Escherichia coli*	ST43	aac(6')-Ib-cr,aac(6')-Ib-cr,blaCTX-M-15,blaOXA-1,blaTEM-1B,dfrA5,mdf(A),qacE,sitABCD,sul1
17589	Blood	2021	MO	*Escherichia coli*	Inconclusive	aac(3)-IId,blaCTX-M-55,mdf(A),qnrS1,sitABCD,tet(A)
17588	Blood	2021	MO	*Escherichia coli*	ST3	blaCTX-M-14,dfrA14,mdf(A),mph(A),sitABCD,tet(B)
17587	Blood	2021	MO	*Escherichia coli*	ST43	blaCTX-M-27,mdf(A),sitABCD
17586	Blood	2021	MO	*Escherichia coli*	ST3293	aph(3')-Ia,blaCTX-M-55,mdf(A),qnrB19,sitABCD

Table S2. Genotypic Results

Cepheid ID	Sample type	Year	State	Organism by K-mer spectra	MLST	Resistance genes identified
17585	Blood	2021	MO	*Escherichia coli*	ST535	aac(3)-IIa,aac(6')-Ib-cr,aph(3'')-Ib,aph(6)-Id,blaCMY-2,blaCTX-M-15,blaOXA-1,blaTEM-1B,catB3,dfrA14,mdf(A),sul2
17584	Blood	2021	MO	*Escherichia coli*	ST43	aac(6')-Ib-cr,aadA5,blaCTX-M-15,blaOXA-1,catB3,dfrA17,mdf(A),mph(A),qacE,sitABCD,sul1,tet(A)
17583	Blood	2021	MO	*Escherichia coli*	ST3	blaCTX-M-14,dfrA14,mdf(A),mph(A),sitABCD,tet(B)
17582	Urine	2021	MO	*Klebsiella pneumoniae*	ST37	aac(3)-IIa,aac(6')-Ib-cr,aph(3'')-Ib,aph(6)-Id,blaCTX-M-15,blaOXA-1,blaSHV-187,blaTEM-1B,catB3,dfrA14,fosA6,OqxA,OqxB,qnrB1,sul2,tet(A)
17581	Urine	2021	MO	*Escherichia coli*	ST736	aadA2,aadA5,aph(3'')-Ib,aph(6)-Id,blaCTX-M-65,blaTEM-1B,dfrA12,dfrA17,mdf(A),mph(A),qacE,sitABCD,sul1,sul2,sul3,tet(A)
17580	Urine	2021	MO	*Escherichia coli*	ST43	aac(6')-Ib-cr,blaCTX-M-15,blaOXA-1,blaTEM-1B,dfrA5,mdf(A),qacE,sitABCD,sul1
17579	Urine	2021	MO	*Klebsiella pneumoniae*	ST307	aac(3)-IIa,aac(6')-Ib-cr,aph(3'')-Ib,aph(6)-Id,blaCTX-M-15,blaOXA-1,blaSHV-28/106/171/205,blaTEM-1B,catB3,dfrA14,fosA6,OqxA,OqxB,sul2
17578	Urine	2021	MO	*Klebsiella pneumoniae*	ST107	aac(3)-IIa,aac(6')-Ib-cr,aph(3'')-Ib,aph(6)-Id,blaCTX-M-15,blaOXA-1,blaSHV-187,blaTEM-1B,catB3,dfrA14,fosA,OqxA,OqxB,qnrB1,sul2,tet(A)
17577	Urine	2021	MO	*Klebsiella pneumoniae*	ST107	aac(3)-IIa,aac(6')-Ib-cr,aph(3'')-Ib,aph(6)-Id,blaCTX-M-15,blaOXA-1,blaSHV-187,blaTEM-1B,catB3,dfrA14,fosA,OqxA,OqxB,qnrB1,sul2,tet(A)
17576	Urine	2021	MO	*Escherichia coli*	ST43	aac(6')-Ib-cr,aadA5,blaCTX-M-15,blaOXA-1,catB3,dfrA17,mdf(A),mph(A),qacE,sitABCD,sul1,tet(A)
17575	Urine	2021	MO	*Escherichia coli*	ST471	aadA5,dfrA17,mdf(A),mph(A),qacE,sitABCD,sul1,tet(A)
17574	Urine	2021	MO	*Escherichia coli*	ST43	aac(3)-IIa,aac(6')-Ib-cr,blaCTX-M-15,blaOXA-1,catB3,mdf(A),sitABCD,tet(A)

Table S2. Genotypic Results

Cepheid ID	Sample type	Year	State	Organism by K-mer spectra	MLST	Resistance genes identified
17573	Urine	2021	MO	*Escherichia coli*	Inconclusive	blaCTX-M-15,mdf(A),sitABCD
17572	Urine	2021	MO	*Klebsiella pneumoniae*	ST3293	aph(3'')-Ib,aph(6)-Id,blaCTX-M-15,blaSHV-187,dfrA14,fosA6,OqxA,OqxB,qnrB1,sul2,tet(A)
**17571**	Blood	2021	WA	*Enterobacter hormaechei supsp. steigerwaltii*	ST190	blaACT-7,fosA
17570	Blood	2021	WA	*Pseudomonas aeruginosa*	ST179	aph(3')-IIb,blaOXA-396,blaPDC-442,catB7,crpP,fosA
17569	Blood	2021	WA	*Escherichia coli*	ST3	aph(3'')-Ib,aph(6)-Id,blaCMY-2,blaTEM-1B,dfrA14,mdf(A),qnrS1,sitABCD,sitABCD,sul2,tet(A)
17568	Blood	2021	WA	*Escherichia coli*	ST44	aac(3)-IIa,aac(6')-Ib-cr,aadA5,aph(3'')-Ib,aph(6)-Id,truncated blaCTX-M-15-like,blaOXA-1,catB3,dfrA17,mdf(A),mph(A),qacE,sitABCD,sul1,sul2,tet(A)
17567	Blood	2021	WA	*Escherichia coli*	ST53	aac(3)-IId,aadA5,blaCTX-M-55,blaTEM-1B,dfrA17,erm(B),mdf(A),mph(A),qacE,sitABCD,sul1
17566	Blood	2021	WA	*Escherichia coli*	ST53	aac(3)-IId,aadA5,blaCTX-M-55,blaTEM-1B,dfrA17,erm(B),mdf(A),mph(A),qacE,sitABCD,sul1
17565	Blood	2021	WA	*Serratia marcescens*		aac(6')-Ic,blaSRT-2
17564	Blood	2021	WA	*Escherichia coli*	ST8	aac(3)-IIa,aadA2,blaCTX-M-14,blaTEM-1B,dfrA12,dfrA5,erm(B),mdf(A),mph(A),qacE,sitABCD,sul1
17563	Blood	2021	WA	*Pseudomonas aeruginosa*	ST1202	aph(3')-IIb,blaOXA-494/396,blaPDC-445,catB7,crpP,fosA
17562	Blood	2021	WA	*Klebsiella michiganensis*	ST30	blaOXY-2-7,fosA7

Table S2. Genotypic Results

Cepheid ID	Sample type	Year	State	Organism by K-mer spectra	MLST	Resistance genes identified
17561	Blood	2021	WA	*Klebsiella pneumoniae*	ST307	aac(3)-IIa,aac(6')-Ib-cr,aph(3'')-Ib,aph(6)-Id,blaCTX-M-15,blaOXA-1,blaSHV-205/171/106/28,blaTEM-1B,catB3,dfrA14,fosA6,OqxA,OqxB,qnrB1,sul2,tet(A)
17560	Urine	2021	WA	*Escherichia coli*	ST87	ant(3'')-Ia,aph(3')-Ia,blaCTX-M-55,blaTEM-1B,lnu(F),mdf(A),qnrS1,sitABCD,sul2,sul3,tet(A)
**17559**	Urine	2021	WA	*Enterobacter cloacae complex*	ST78	aac(6')-Ib,ant(3'')-Ia,blaACT-5,blaCTX-M-3,blaLAP-2,blaOXA-10,blaTEM-1B,dfrA15,dfrB4,fosA,qacE,qnrS1,sul1,tet(C)
17558	Urine	2021	WA	*Escherichia coli*	ST721	blaCTX-M-15,mdf(A),sitABCD,sul2,tet(B)
17557	Urine	2021	WA	*Escherichia coli*	ST132	aph(3'')-Ib,aph(6)-Id,truncated blaCTX-M,blaTEM-1B,dfrA7,mdf(A),qacE,qnrS1,sul1,sul2,tet(A)
17556	Urine	2021	WA	*Escherichia coli*	ST43	blaCTX-M-15,blaTEM-1B,mdf(A),sitABCD,sitABCD
17555	Urine	2021	WA	*Escherichia coli*	ST43	blaCTX-M-14,blaTEM-1C,dfrA14,mdf(A),mph(A),sitABCD
17554	Urine	2021	WA	*Escherichia coli*	ST43	ant(3'')-Ia,blaCTX-M-15,blaTEM-1B,dfrA15,mdf(A),qacE,sitABCD,sul1,tet(A)
17553	Urine	2021	WA	*Klebsiella pneumoniae*	ST307	aph(3'')-Ib,aph(6)-Id,blaCTX-M-15,blaSHV-205/171/106/28,blaTEM-1B,dfrA14,fosA6,OqxA,OqxB,qnrB1,sul2,tet(A)
17552	Urine	2021	WA	*Escherichia coli*	ST43	blaCTX-M-14,blaTEM-1B,mdf(A),sitABCD
17551	Urine	2021	WA	*Pseudomonas aeruginosa*	ST309	aph(3')-IIb,blaOXA-50,blaPDC-19a,catB7,crpP,fosA
**17550**	Urine	2021	WA	*Enterobacter hormaechei*	ST102	aac(3)-IVa,aac(6')-Ib-cr,aadA2,aph(4)-Ia,ARR-3,blaACT-5,blaOXA-1,catB3,dfrA12,floR,fosA,qacE,qnrA1,sul1,sul2,tet(B)

Table S2. Genotypic Results

Cepheid ID	Sample type	Year	State	Organism by K-mer spectra	MLST	Resistance genes identified
**17549**	Urine	2021	WA	*Enterobacter hormaechei*	ST113	aac(6')-Ib-cr,ant(3'')-Ia,aph(3'')-Ib,aph(6)-Id,blaACT-15,blaCTX-M-15,blaOXA-1,blaTEM-1B,catA1,catB3,dfrA14,fosA,sul2,tet(A)
17548	Urine	2021	WA	*Klebsiella pneumoniae*	ST15	aadA2,blaCTX-M-15,blaSHV-28/106/171/205,blaTEM-1B,dfrA12,dfrA16,fosA6,mph(A),OqxA,OqxB,qacE,qnrB2,sul1
17547	Urine	2021	WA	*Klebsiella pneumoniae*	ST15	aadA2,blaCTX-M-15,blaSHV-205/106/187/28,blaTEM-1B,dfrA12,fosA6,mph(A),OqxA,OqxB,qacE,sul1
17546	Urine	2021	WA	*Escherichia coli*	ST43	aac(6')-Ib-cr,aadA2,blaCTX-M-15,blaOXA-1,catB3,dfrA12,mdf(A),mph(A),qacE,sitABCD,sul1
17545	Blood	2021	NM	*Escherichia coli*	ST879	blaCTX-M-14 or CTX-M-24,dfrA14,mdf(A),sitABCD,sul2
17544	Blood	2021	NM	*Escherichia coli*	ST535	blaCTX-M-15,mdf(A),qnrS1,sitABCD,tet(A)
17543	Blood	2021	NM	*Escherichia coli*	ST700	ant(3'')-Ia,aph(3'')-Ib,aph(6)-Id,blaCTX-M-15,blaTEM-1B,dfrA1,mdf(A),sul2
17329	Urine	2021	GA	*Acinetobacter baumannii*	ST2	aac(3)-Ia,aac(6')-Ian,aph(3'')-Ib,aph(6)-Id,blaADC-25,blaOXA-23, blaOXA-66,qacE,sul1,tet(B)
17541	Blood	2021	NM	*Klebsiella pneumoniae*	ST5193-like	blaSHV-187,fosA,fosA5,OqxA,OqxB
17540	Blood	2021	NM	*Klebsiella pneumoniae*	ST17	aph(3')-Ia,aph(3'')-Ib,aph(6)-Id,blaSHV-187,catA2,fosA,mcr-9,OqxA,OqxB
17539	Blood	2021	NM	*Escherichia coli*	ST43	aac(3)-IIa,aac(6')-Ib-cr,aadA5,blaCTX-M-15,blaOXA-1,catB3,dfrA17,mdf(A),mph(A),qacE,sitABCD,sul1,tet(A)
17538	Blood	2021	NM	*Escherichia coli*	ST2	aadA1,aadA5,aph(3')-Ia,aph(3'')-Ib,aph(6)-Id,blaCMY-2,blaCTX-M-15,blaOXA-1,catB3,dfrA17,floR,mdf(A),mph(A),qacE,rmtE,sitABCD,sul1,sul2,tet(A),tet(B)

Table S2. Genotypic Results

Cepheid ID	Sample type	Year	State	Organism by K-mer spectra	MLST	Resistance genes identified
17537	Blood	2021	NM	*Escherichia coli*	ST477	aadA2,aadA5,ant(3'')-Ia,aph(3'')-Ib,aph(6)-Id,blaCTX-M-55,cmlA1,dfrA17,mdf(A),qacE,sitABCD,sul1,sul2,sul3,tet(A)
**17536**	Blood	2021	NM	*Enterobacter hormaechei supsp. xiangfangensis*	ST451	blaACT-84,fosA
17535	Urine	2021	NM	*Klebsiella pneumoniae*	ST866	aac(6')-Ib-cr,aac(6')-Ib-cr,aph(3'')-Ib,aph(6)-Id,blaCTX-M-15,blaOXA-1,blaSHV-187,blaTEM-1B,catB3,dfrA14,fosA6,OqxA,OqxB,qnrB1,sul2,tet(A)
**17534**	Urine	2021	NM	*Enterobacter hormaechei*	ST45	aph(3'')-Ib,aph(6)-Id,blaACT-15,blaTEM-1B,dfrA14,sul2
17533	Urine	2021	NM	*Pseudomonas aeruginosa*	ST2615	aph(3')-IIb,blaOXA-50,blaPDC-368,catB7,fosA
17531	Urine	2021	NM	*Klebsiella pneumoniae*	ST3964	aac(6')-Ib-cr,aac(6')-Ib-cr,aph(3'')-Ib,aph(6)-Id,blaCTX-M-15,blaLEN-13,blaOXA-1,blaTEM-1B,dfrA14,fosA,fosA7,OqxA,OqxB,sul2
17530	Urine	2021	NM	*Pseudomonas aeruginosa*	ST235	aadA6,aph(3')-IIa,aph(3')-IIb,aph(6)-Ic,blaOXA-488,blaPDC-35,catB7,fosA,qacE,sul1
**17529**	Urine	2021	NM	*Enterobacter cloacae complex*	ST108	blaACT-7
17528	Urine	2021	NM	*Hafnia alvei*	Not available	blaACC-1a
17527	Urine	2021	NM	*Klebsiella michiganensis*	ST11	aac(3)-IIa,aac(6')-Ib-cr,aph(3')-Ia,aph(3'')-Ib,aph(6)-Id,blaCTX-M-15,blaOXA-1,blaOXY-1-1,blaTEM-1B,catB3,dfrA14,qnrB1,sul2,tet(A)
**17526**	Urine	2021	NM	*Enterobacter cloacae complex*	ST108	blaACT-7
**17525**	Urine	2021	NM	*Enterobacter hormaechei*	ST1377	aac(6')-Ib-cr,aadA16,ant(3'')-Ia,ARR-3,blaACT-7,blaNDM-1,blaOXA-10,dfrA27,qacE,qnrS1,sul1

Table S2. Genotypic Results

Cepheid ID	Sample type	Year	State	Organism by K-mer spectra	MLST	Resistance genes identified
17524	Urine	2021	NM	*Pseudomonas aeruginosa*	ST2211	aph(3')-IIb,aph(3')-IIc,aph(6)-Ic,blaOXA-395,blaPDC-342,catB7,crpP,crpP,formA,formA,OqxA
17523	Urine	2021	NM	*Raoultella ornithinolytica*	Not available	blaOXA-181, blaORN-1,fosA,qnrS1
17522	Urine	2021	NM	*Escherichia coli*	ST43	aac(3)-IIa,blaCTX-M-15,blaOXA-1,catB3,mdf(A),sitABCD
17521	Urine	2021	NM	*Klebsiella pneumoniae*	ST307	aac(3)-IIa,aac(6')-Ib-cr,aph(3'')-Ib,aph(6)-Id,blaCTX-M-15,blaOXA-1,blaSHV-205,blaTEM-1B,catB3,dfrA14,fosA6,OqxA,OqxB,qnrB1,sul2,tet(A)
17520	Urine	2021	NM	*Escherichia coli*	ST43	aac(3)-IIa,aac(6')-Ib-cr,aac(6')-Ib-cr,aadA5,blaCTX-M-15,blaOXA-1,catB3,dfrA17,mdf(A),qacE,sitABCD,sul1,tet(A)
17464	Blood	2021	NY	*Escherichia coli*	ST43	aac(6')-Ib-cr,aadA5,blaCTX-M-15,blaOXA-1,catB3,dfrA17,mdf(A),mph(A),qacE,sitABCD,sul1,tet(A)
17463	Blood	2021	NY	*Escherichia coli*	ST43	aadA5,aph(3'')-Ib,aph(6)-Id,blaCTX-M-27,dfrA17,mdf(A),mph(A),qacE,sitABCD,sul1,sul2,tet(A)
17462	Blood	2021	NY	*Escherichia coli*	ST43	aac(6')-Ib-cr,aadA5,blaCTX-M-15,blaOXA-1,catB3,dfrA17,mdf(A),mph(A),qacE,sitABCD,sul1,tet(A)
**17461**	Blood	2021	NY	*Enterobacter ludwigii*	Inconclusive	blaACT-12,fosA2,fosA7
17460	Blood	2021	NY	*Serratia fonticola*	Not available	blaFONA-6
17459	Blood	2021	NY	*Escherichia coli*	ST43	aadA5,aph(3'')-Ib,aph(6)-Id,blaCTX-M-27,dfrA17,mdf(A),mph(A),qacE,sitABCD,sul1,sul2,tet(A)
17458	Blood	2021	NY	*Escherichia coli*	ST3	aadA5,ant(3'')-Ia,blaCTX-M-15,dfrA1,mdf(A),qacE,qnrS1,sitABCD,sul1,sul2,tet(B)

Table S2. Genotypic Results

Cepheid ID	Sample type	Year	State	Organism by K-mer spectra	MLST	Resistance genes identified
17457	Blood	2021	NY	*Escherichia coli*	ST24	aph(3'')-Ib,aph(6)-Id,blaCMY-2,blaTEM-1B,dfrA5,mdf(A),sitABCD,sul2,tet(A)
17456	Blood	2021	NY	*Citrobacter freundii*	Not available	blaCMY-98,qnrB69
17455	Blood	2021	NY	*Escherichia coli*	ST8	aph(3'')-Ib,aph(6)-Id,blaCTX-M-14,mdf(A),sul2
17454	Blood	2021	NY	*Escherichia coli*	ST43	aac(3)-IIa,aac(6')-Ib-cr,blaCTX-M-15,blaOXA-1,catB3,mdf(A),sitABCD
17453	Blood	2021	NY	*Klebsiella aerogenes*	ST4	blaACT-14, formA, fosA, mdf(A),OqxA,OqxB,tet(34), sitABCD (all <90%)
17452	Blood	2021	NY	*Escherichia coli*	ST53	aac(3)-IId,aadA5,aph(3'')-Ib,aph(6)-Id,blaCTX-M-15,blaTEM-1B,dfrA17,mdf(A),mph(A),qacE,sitABCD,sul1,sul2,tet(A)
17451	Blood	2021	NY	*Escherichia coli*	ST479	aac(3)-IId,blaCTX-M-1,dfrA7,mdf(A),mph(A),qacE,sul1,tet(A)
17450	Blood	2021	NY	*Klebsiella pneumoniae*	ST307	aac(3)-IIa,aac(6')-Ib-cr,aph(3'')-Ib,aph(6)-Id,blaCTX-M-15,blaOXA-1,blaSHV-28/106,blaTEM-1B,catB3,dfrA14,fosA6,OqxA,OqxB,qnrB1,sul2,tet(A)
17449	Urine	2021	NY	*Klebsiella pneumoniae*	ST307	aac(3)-IIa,aph(3'')-Ib,aph(6)-Id,blaCTX-M-15,blaSHV-28/106,blaTEM-1B,dfrA14,fosA6,OqxA,OqxB,qnrB1,sul2
17448	Urine	2021	NY	*Klebsiella michiganensis*	ST27	aac(3)-IIa,aac(6')-Ib-cr,aadA5,aph(3')-Ia,aph(3'')-Ib,aph(6)-Id,blaCTX-M-15,blaOXA-1,blaOXY-1-1,blaTEM-1B,catA1,catB3,dfrA14,dfrA17,mph(A),qacE,qnrB1,sul1,sul2,tet(A),tet(B)
17447	Urine	2021	NY	*Escherichia coli*	Inconclusive	aac(3)-IId,aadA5,aph(3'')-Ib,aph(6)-Id,blaCTX-M-15,blaTEM-1B,dfrA17,mdf(A),mph(A),qacE,sitABCD,sul1,sul2,tet(A)
17446	Urine	2021	NY	*Escherichia coli*	Inconclusive	aac(3)-IIa,aac(6')-Ib-cr,aadA5,blaCTX-M-15,blaOXA-1,catB3,dfrA17,mdf(A),mph(A),qacE,sitABCD,sul1,tet(A)

Table S2. Genotypic Results

Cepheid ID	Sample type	Year	State	Organism by K-mer spectra	MLST	Resistance genes identified
17445	Urine	2021	NY	*Escherichia coli*	ST53	aadA5,aph(3'')-Ib,aph(6)-Id,blaCTX-M-27,dfrA17,mdf(A),mph(A),qacE,sitABCD,sul1,sul2,tet(A)
17444	Urine	2021	NY	*Escherichia coli*	ST1	blaCTX-M-14,mdf(A),sitABCD
17443	Urine	2021	NY	*Klebsiella pneumoniae*	ST307	aph(3'')-Ib,aph(6)-Id,blaCTX-M-15,blaKPC-3,blaSHV-28/106,blaTEM-1B,dfrA14,fosA6,OqxA,OqxB,sul2
17442	Urine	2021	NY	*Klebsiella pneumoniae*	ST348	ant(2'')-Ia,blaKPC-4,blaOXA-2,blaSHV-187,fosA,OqxA,OqxB,qnrB2,sul1
17330	Urine	2021	GA	*Acinetobacter baumannii*	ST2	aph(3'')-Ib,aph(6)-Id,blaADC-25,blaOXA-80,tet(B)
**17440**	Urine	2021	NY	*Enterobacter hormaechei*	ST133	blaACT-7,fosA,mcr-9
**17439**	Urine	2021	NY	*Klebsiella pneumoniae*	ST258	aac(6')-Ib,aadA5,ant(3'')-Ia,blaKPC-3,blaOXA-9,blaSHV-187,blaTEM-1A,dfrA17,dfrA5,fosA6,OqxA,OqxB,qacE,sul1,tet(D)
17339	Blood	2021	GA	*Acinetobacter baumannii*	ST2	aac(3)-Ia,aac(6')-Ian,aph(3'')-Ib,aph(6)-Id,blaADC-25,blaOXA-23,blaOXA-66,qacE,sul1,tet(B)
**17437**	Urine	2021	NY	*Enterobacter hormaechei*	ST636	blaACT-7,fosA
17435	Urine	2021	NY	*Acinetobacter baumannii*	ST499	ant(2'')-Ia,blaADC-25,blaOXA-23,blaOXA-95,catB3,qacE,sul1
17436	Urine	2021	NY	*Acinetobacter baumannii*	ST2	aph(3')-VIa,blaADC-25,blaOXA-23,blaOXA-82,mph(E),msr(E)
17430	Blood	2021	NJ	*Escherichia coli*	ST43	blaDHA-1,dfrA17,mdf(A),mph(A),qacE,qnrB4,sitABCD,sitABCD,sul1

Table S2. Genotypic Results

Cepheid ID	Sample type	Year	State	Organism by K-mer spectra	MLST	Resistance genes identified
17429	Blood	2021	NJ	*Escherichia coli*	ST3	aadA5,aph(3'')-Ib,aph(6)-Id,blaCMY-2,blaTEM-1B,dfrA17,mdf(A),mph(A),qacE,sitABCD,sul1,sul2,tet(B)
17428	Blood	2021	NJ	*Escherichia coli*	ST43	blaCTX-M-27,mdf(A),sitABCD
**17427**	Blood	2021	NJ	*Klebsiella pneumoniae*	ST307	aac(3)-IIa,aac(6')-Ib-cr,ant(3'')-Ia,aph(3'')-Ib,aph(6)-Id,blaCTX-M-15,blaOXA-1,blaSCO-1,blaSHV-28/106,blaTEM-1B,catA1,catB3,dfrA8,fosA6,OqxA,OqxB,qacE,qnrB1,sul1,sul2,tet(A),tet(B),tet(C)
17426	Blood	2021	NJ	*Escherichia coli*	ST9	aac(3)-IId,aadA2,aph(3'')-Ib,aph(6)-Id,blaCTX-M-15,blaTEM-1B,dfrA12,mdf(A),mph(A),qacE,sul1,sul2,tet(A)
17425	Blood	2021	NJ	*Escherichia coli*	ST43	blaCTX-M-27,mdf(A),sitABCD
17424	Blood	2021	NJ	*Pseudomonas aeruginosa*	ST274	aph(3')-IIb,blaOXA-486,blaPDC-24,catB7,fosA
17346	Blood	2021	KS	*Escherichia coli*	ST53	aac(3)-IIa,aac(6')-Ib-cr,aph(3'')-Ib,aph(6)-Id,blaCTX-M-15,blaOXA-1,blaTEM-1B,catB3,dfrA14,erm(B),mdf(A),qnrB1,sitABCD,sul2,tet(A)
**17345**	Urine	2021	KS	*Klebsiella pneumoniae*	ST25	aph(3'')-Ib,aph(6)-Id,blaCTX-M-15,blaSHV-187,blaTEM-1B,dfrA14,fosA6,OqxA,OqxB,,qnrB1,sul2
**17344**	Urine	2021	KS	*Klebsiella pneumoniae*	ST307	aph(3'')-Ib,aph(6)-Id,blaCTX-M-15,blaSHV-106/28 (blaSHV-205 by BLAST),blaTEM-1B,dfrA14,fosA6,OqxA,OqxB,qnrB1,sul2,tet(A)
17343	Urine	2021	KS	*Escherichia coli*	ST3	blaCTX-M-15,mdf(A),qnrS1,sitABCD
17342	Urine	2021	KS	*Escherichia coli*	Inconclusive	aadA2,ant(3'')-Ia,aph(3')-Ia,aph(3'')-Ib,blaCTX-M-27,blaTEM-1C,cml,cmlA1,dfrA12,erm(B),mdf(A),mph(A),sitABCD,sul2,sul3,tet(A)
**17340**	Blood	2021	GA	*Klebsiella pneumoniae*	ST16	aac(3)-IIa,aac(6')-Ib-cr,aadA2,ant(3'')-Ia,aph(3')-Ia,aph(3'')-Ib,aph(6)-Id,blaCTX-M-15,blaNDM-5,blaOXA-1,blaOXA-181,blaSHV-187,blaTEM-1B,dfrA12,dfrA14,floR,fosA5,mph(A),OqxA,OqxB,qacE,qnrS1,rmtB,sul1,sul2,sul3

Table S2. Genotypic Results

Cepheid ID	Sample type	Year	State	Organism by K-mer spectra	MLST	Resistance genes identified
17438	Urine	2021	NY	*Acinetobacter baumannii*	ST2	aac(6')-Ib,aac(6')-Ib-cr,aadA1,aph(3')-Ia,aph(3'')-Ib,aph(6)-Id,blaADC-25,blaOXA-407,blaTEM-1D,catB8,mph(E),qacE,sul1,sul2,tet(B)
**17338**	Urine	2021	GA	*Klebsiella oxytoca*	ST180	aac(6')-Ib,aadA13,ant(3'')-Ia,aph(3')-Ia,blaKPC-3,blaOXA-9,blaOXY-1-4,blaTEM-1A,fosA,OqxA,OqxB,qnrB19
**17337**	Urine	2022	GA	*Klebsiella pneumoniae*	ST219	aph(3'')-Ib,aph(6)-Id,blaCTX-M-15,blaKPC-3,blaSHV-187,fosA,OqxA,OqxB,qnrS1,sul2,tet(A)
**17336**	Blood	2022	GA	*Klebsiella pneumoniae*	ST13	blaKPC-3,blaSHV-187,blaTEM-1A,dfrA14,fosA6,OqxA,OqxB,qnrB1
17335	Urine	2021	GA	*Klebsiella aerogenes*	Inconclusive	blaKPC-3
**17334**	Urine	2022	GA	*Klebsiella pneumoniae*	ST219	aph(3'')-Ib,aph(6)-Id,blaCTX-M-15,blaKPC-3,blaSHV-187,fosA,OqxA,OqxB,qnrS1,sul2,tet(A)
17333	Urine	2021	GA	*Klebsiella aerogenes*	Inconclusive	aac(6')-Ib,ant(3'')-Ia,blaKPC-3,blaOXA-9,blaTEM-1A,qnrB19
**17332**	Urine	2021	GA	*Klebsiella pneumoniae*	ST16	aac(3)-IIa,aac(6')-Ib-cr,ant(3'')-Ia,aph(3')-Ia,aph(3'')-Ib,aph(6)-Id,blaCTX-M-15,blaOXA-1,blaSHV-187,blaTEM-176,catB3,dfrA14,floR,fosA5,OqxA,OqxB,sul2,sul3
17331	Urine	2021	GA	*Klebsiella pneumoniae*	ST16	aac(3)-IIa,aac(6')-Ib-cr,aadA2,ant(3'')-Ia,aph(3')-Ia,aph(3'')-Ib,aph(6)-Id,blaCTX-M-15,blaNDM-5,blaOXA-1,blaOXA-181,blaSHV-187,
17441	Urine	2021	NY	*Acinetobacter baumannii*	ST2	aph(3')-VIa,blaADC-25,blaOXA-23,blaOXA-82,mph(E),msr(E)
17542	Blood	2021	NM	*Acinetobacter beijerinckii*	Inconclusive	qacE
**17328**	Urine	2021	GA	*Enterobacter hormaechei*	ST-427	blaACT-15,fosA

Table S2. Genotypic Results

Cepheid ID	Sample type	Year	State	Organism by K-mer spectra	MLST	Resistance genes identified
**17327**	Urine	2021	GA	*Klebsiella pneumoniae*	ST469	aac(6')-Ib,ant(3'')-Ia,blaKPC-3,blaOXA-9,blaSHV-187,blaTEM-1A,fosA,fosA6,OqxA,OqxB,qnrB19
**17326**	Urine	2021	GA	*Klebsiella oxytoca*	ST-50	aac(3)-IIa,aac(6')-Ib-cr,aac(6')-Ib-cr,aph(3')-Ia,aph(3'')-Ib,aph(6)-Id,blaCTX-M-15,blaOXA-1,blaOXY-1-2,blaTEM-1B,catB3,dfrA14,qnrB1,sul2,tet(A)
**17325**	Urine	2021	GA	*Klebsiella pneumoniae*	ST379	aac(6')-Ib,aadA1,aadA2,blaKPC-3,blaOXA-9,blaSHV-187,blaTEM-168/1A/54,catA1,dfrA12,fosA6,OqxA,OqxB,qacE,sul1
17635	Blood	2021	NM	*Acinetobacter baumannii*	ST203	blaADC-25,blaOXA-78
**17323**	Blood	2021	NJ	*Klebsiella pneumoniae*	ST231	aac(6')-Ib,aadA2,ARR-3,blaCTX-M-15,blaSHV-187,blaTEM-1B,catA1,dfrA12,erm(B),fosA,mph(A),OqxA,OqxB,qacE,rmtF,sitABCD,sul1
**17322**	Blood	2021	NJ	*Klebsiella pneumoniae*	ST1683-like	blaKPC-3,blaLEN16,blaSHV-187,blaTEM-1B,dfrA14,fosA,OqxA,OqxB,qnrS1
17321	Blood	2021	NJ	*Klebsiella aerogenes*	ST92	aac(6')-Ib-Hangzhou,blaCTX-M-15,blaTEM-1B,fosA,rmtF
**17320**	Blood	2021	NJ	*Klebsiella pneumoniae*	ST231	aac(6')-Ib,aadA2,ARR-3,blaCTX-M-15,blaSHV-187,blaTEM-1B,catA1,dfrA12,erm(B),fosA,mph(A),OqxA,OqxB,qacE,rmtF,sitABCD,sul1
17319	Blood	2021	NJ	*Escherichia coli*	ST43	aac(3)-IIa,aac(6')-Ib-cr,aadA5,aph(3'')-Ib,aph(6)-Id,blaCTX-M-15,blaOXA-1,blaTEM-1B,catB3,dfrA17,mdf(A),mph(A),qacE,sitABCD,sul1,tet(A)
17318	Blood	2021	NJ	*Escherichia coli*	ST43	aadA5,blaCTX-M-15,dfrA17,mph(A),qacE,sul1
**17317**	Blood	2021	NJ	*Enterobacter cloacae*	ST50	blaACT-15,fosA
17711	Urine	2021	CA	*Acinetobacter baumannii*	ST258	aph(3'')-Ib,aph(3')-VIa,aph(6)-Id,armA,blaADC-25,blaOXA-23,blaOXA-66,mph(E),msr(E),qacE,sul1,tet(B)

Table S2. Genotypic Results

Cepheid ID	Sample type	Year	State	Organism by K-mer spectra	MLST	Resistance genes identified
17315	Urine	2021	NJ	*Pseudomonas aeruginosa*	ST235	aadA6,aph(3'')-Ib,aph(3')-IIb,aph(6)-Id,blaOXA-488,blaPDC-35,catB7,crpP,fosA,qacE,sul1
**17314**	Urine	2021	NJ	*Klebsiella pneumoniae*	ST258	aac(6')-Ib,aadA6,aph(3'')-Ib,aph(3')-IIb,aph(6)-Id,blaKPC-3,blaOXA-9,blaTEM-1A,catA1,catB7,crpP,fosA,fosA6,OqxA,OqxB,qacE,sul1
**17313**	Urine	2021	NJ	*Klebsiella pneumoniae*	ST258	aac(6')-Ib,ant(3'')-Ia,aph(3'')-Ib,aph(6)-Id,blaKPC-3,blaOXA-9,blaSHV-12,blaTEM-1A,dfrA14,fosA6,mph(A),OqxA,OqxB,sul2
17312	Urine	2021	NJ	*Escherichia coli*	ST53	aadA5,aph(3'')-Ib,aph(6)-Id,blaCTX-M-27,dfrA17,erm(B),mph(A),qacE,sul1,sul2,tet(A)
**17311**	Urine	2021	NJ	*Klebsiella pneumoniae*	ST412	blaKPC-2,blaSHV-187,fosA6,OqxA,OqxB,qnrS1
**17310**	Urine	2021	NJ	*Enterobacter cloacae*	Inconclusive	blaMIR-5,fosA
17309	Urine	2021	NJ	*Escherichia coli*	Inconclusive	aph(3'')-Ib,aph(6)-Id,blaCTX-M-27,blaTEM-1B,mdf(A),sitABCD,sul2,tet(A)
**17308**	Urine	2021	NJ	*Enterobacter cloacae*	ST108	blaACT-7
**17307**	Urine	2021	NJ	*Enterobacter hormaechei supsp. xiangfangensis*	ST171	aac(6')-Ib,ant(3'')-Ia,aph(3')-Ia,aph(3'')-Ib,aph(6)-Id,blaACT-16,blaKPC-3,blaOXA-9,blaTEM-1A,dfrA14,sul2
**17306**	Urine	2021	NJ	*Klebsiella pneumoniae*	ST412	blaKPC-2,blaSHV-187,fosA6,OqxA,OqxB,qnrS1
17305	Urine	2021	NJ	*Escherichia coli*	ST43	aadA5,blaCTX-M-15,dfrA17,mdf(A),qacE,sitABCD,sul1
**17304**	Urine	2021	NJ	*Klebsiella pneumoniae*	ST258	aac(6')-Ib,ant(3'')-Ia,aph(3'')-Ib,aph(6)-Id,blaKPC-3,blaOXA-9,blaSHV-12,blaTEM-1A,dfrA14,fosA6,mph(A),OqxA,OqxB,sul2

Table S2. Genotypic Results

Cepheid ID	Sample type	Year	State	Organism by K-mer spectra	MLST	Resistance genes identified
17303	Urine	2021	NJ	*Klebsiella aerogenes*	ST103	fosA, ompK36 (p.A93S) (ceph), ompA, LptD
17302	Urine	2021	NJ	*Pseudomonas aeruginosa*	ST164	aph(3')-IIb,blaOXA-486,blaPDC-121,catB7,crpP,fosA
17301	Urine	2021	NJ	*Pseudomonas aeruginosa*	ST235	aadA6,aph(3'')-Ib,aph(3')-IIb,aph(6)-Id,blaOXA-488,blaPDC-35,catB7,crpP,fosA,qacE,sul1
17247	Urine	2021	KS	*Pseudomonas aeruginosa*	ST1337	aph(3')-IIb,blaOXA-50,blaPDC-471,catB7,crpP,fosA
**17246**	Urine	2021	KS	*Enterobacter cloacae*	ST191	blaACT-9,fosA
17245	Urine	2021	KS	*Serratia marcescens*	Not available	aac(6')-Ic,blaSRT-2,tet(41)
17244	Urine	2021	KS	*Escherichia coli*	ST8	blaCTX-M-14,mdf(A)
17243	Urine	2021	KS	*Escherichia coli*	ST53	aph(3'')-Ib,aph(6)-Id,blaCTX-M-15,blaTEM-1B,dfrA14,mdf(A),qacE,sitABCD,sul2
17213	Urine	2021	KS	*Providencia rettgeri*	Not available	tet(57), CRP (CARD) (80% min identity)
17212	Urine	2021	KS	*Escherichia coli*	ST43	blaCTX-M-15,mdf(A),sitABCD
17211	Urine	2021	KS	*Escherichia coli*	ST24	aadA5,aph(3'')-Ib,aph(6)-Id,blaCTX-M-1,blaTEM-1B,dfrA17,dfrA5,mdf(A),sitABCD,sul2
17210	Urine	2021	KS	*Escherichia coli*	ST958	aac(3)-IIa,ant(3'')-Ia,aph(3')-Ia,aph(3'')-Ib,aph(6)-Id,blaCTX-M-55,blaTEM-1B,dfrA14,floR,mdf(A),qnrB19,sitABCD,sul2,sul3,tet(A)

Table S2. Genotypic Results

Cepheid ID	Sample type	Year	State	Organism by K-mer spectra	MLST	Resistance genes identified
17209	Urine	2021	KS	*Escherichia coli*	ST958	aac(3)-IIa,ant(3'')-Ia,aph(3')-Ia,aph(3'')-Ib,aph(6)-Id,blaCTX-M-55,blaTEM-1B,dfrA14,floR,mdf(A),qnrB19,sitABCD,sul2,sul3,tet(A)
**17208**	Urine	2021	KS	*Enterobacter cloacae*	ST171	aac(6')-Ib-cr,ant(3'')-Ia,aph(3'')-Ib,aph(6)-Id,blaACT-16,blaCTX-M-15,blaOXA-1,blaTEM-1B,catB3,dfrA14,fosA,qnrB1,sul2,tet(A)
17207	Urine	2021	KS	*Escherichia coli*	ST66	aac(6')-Ib-cr,aadA5,ant(3'')-Ia,aph(3'')-Ib,aph(6)-Id,blaCTX-M-15,blaOXA-1,catB3,dfrA1,dfrA17,mdf(A),qacE,sitABCD,sul1,sul2,tet(B)
17206	Blood	2021	CA	*Citrobacter freundii*	Not available	blaCMY-67
17205	Urine	2021	CA	*Escherichia coli*	Inconclusive	aac(6')-Ib-cr,aac(6')-Ib-cr,aadA5,blaCTX-M-15,blaKPC-2-like (truncated),blaOXA-1,blaTEM-1B,catB3,dfrA17,erm(B),mdf(A),mph(A),qacE,sul1
17204	Urine	2021	CA	*Escherichia coli*	Inconclusive	aac(3)-IId,aadA5,aph(3'')-Ib,aph(6)-Id,blaCTX-M-27,blaTEM-1B,dfrA17,mdf(A),mph(A),qacE,sitABCD,sul1,sul2,tet(A)
17202	Blood	2021	CA	*Escherichia coli*	Inconclusive	blaCTX-M-14,mdf(A),sitABCD
17201	Blood	2021	CA	*Escherichia coli*	ST221	blaOXA-48,mdf(A),sitABCD
**17200**	Blood	2021	CA	*Klebsiella pneumoniae*	ST11	aac(6')-Ib-cr,aadA2,ARR-3,blaCTX-M-65,blaKPC-2,blaOXA-1,blaSHV-187,blaTEM-1B,catB3,dfrA1,fosA3,fosA6,mph(A),qacE,sul1,tet(A)
17199	Blood	2021	CA	*Serratia marcescens*	Not available	aac(6')-Ic,blaSME-2,blaSST-1,tet(41)
**17198**	Blood	2021	CA	*Escherichia coli*	ST2	aac(6')-Ib-cr,aadA2,aadA5,aph(3'')-Ib,aph(6)-Id,blaCTX-M-15,blaNDM-5,blaOXA-1,catA1,catB3,dfrA12,dfrA17,mdf(A),mph(A),qacE,sitABCD,sul1,sul2,tet(B)
17747	Blood	2021	TN	*Acinetobacter baumannii*	ST2	aac(3)-Ia,ant(3'')-Ia,aph(3'')-Ib,aph(6)-Id,blaADC-25,blaOXA-23,blaOXA-66,qacE,sul1,tet(B)

Table S2. Genotypic Results

Cepheid ID	Sample type	Year	State	Organism by K-mer spectra	MLST	Resistance genes identified
17748	Blood	2021	TN	*Acinetobacter baumannii*	Inconclusive	blaADC-25,blaOXA-24,blaOXA-317
**17195**	Blood	2021	CA	*Pseudomonas aeruginosa*	ST357	aac(6')-II,ant(3'')-Ia,aph(3')-IIb,aph(3')-VI,ARR-3,blaNDM-1,blaOXA-10,blaOXA-50,blaPAO,blaVEB-1,catB7,cml,cmlA1,crpP,dfrB2,fosA,qacE,sul1,tet(A)
17194	Blood	2021	CA	*Escherichia coli*	ST43	aac(3)-IId,aadA2,aadA5,aph(3'')-Ib,aph(6)-Id,blaCTX-M-27,blaTEM-1B,dfrA12,dfrA17,mdf(A),mph(A),qacE,sitABCD,sul1,sul2,tet(A)
**17193**	Blood	2021	CA	*Enterobacter hormaechei supsp. steigerwaltii*	ST636	blaACT-7, fosA
17192	Blood	2021	CA	*Escherichia coli*	ST8	blaCTX-M-27, blaTEM-1B, mdf(A)
**17191**	Blood	2021	CA	*Klebsiella pneumoniae*	ST15	aac(6')-Ib-cr,aadA16,aadA2,ant(3'')-Ia,aph(3')-Ia,aph(3'')-Ib,aph(6)-Id,ARR-3,blaCTX-M-27,blaSHV-106/28,cml,cmlA1,dfrA12,dfrA27,fosA6,oqxA,oqxB,qacE,sul1,sul3,tet(A)
17190	Blood	2021	CA	*Pseudomonas aeruginosa*	ST2676	aph(3')-IIb,blaOXA-395,blaPDC-142,catB7,fosA
17189	Blood	2021	CA	*Pseudomonas aeruginosa*	ST1400	aph(3')-IIb,blaOXA-395,blaPAO,catB7,fosA
17188	Urine	2021	CA	*Klebsiella aerogenes*	ST207	fosA,oqxA,oqxB, ompK36 (p.A217S) (carba), ompK36 (p.L191Q), ompK36 (p.D224E), ompK36 (p.F207W), ompK36 (p.N49S) (ceph)
17187	Urine	2021	CA	*Escherichia coli*	ST5614 (Achtma	aadA5,aph(3'')-Ib,aph(6)-Id,blaCTX-M-15,dfrA17,mdf(A),qacE,qnrS1,sul2,tet(A)
17186	Urine	2021	CA	*Escherichia coli*	ST648 (Achtman	aadA2,blaCMY-42,blaCTX-M-15,dfrA12,erm(B),mdf(A),mph(A),qacE,sul1
**17185**	Urine	2021	CA	*Klebsiella pneumoniae*	ST101	aac(3)-IIa,aac(6')-Ib-cr,blaCTX-M-15,blaOXA-1,blaOXA-48,blaSHV-187,catB3,dfrA14,fosA,oqxA,oqxB

Table S2. Genotypic Results

Cepheid ID	Sample type	Year	State	Organism by K-mer spectra	MLST	Resistance genes identified
**17184**	Urine	2021	CA	*Escherichia coli*	ST44	aadA2,blaCTX-M-15,blaNDM-5,dfrA12,mdf(A),qacE,sitABCD,sul1,tet(B)
17183	Urine	2021	CA	*Escherichia coli*	inconclusive	aadA5,aph(3'')-Ib,aph(6)-Id,blaKPC-2,blaTEM-1B,dfrA17,mdf(A),mph(A),qacE,sitABCD,sul1,sul2,tet(A)
17182	Urine	2021	CA	*Klebsiella michiganensis*	Inconclusive	aac(3)-IIa,aac(3)-IId,aac(6')-Ib-cr,ant(3'')-Ia,aph(3'')-Ib,aph(6)-Id,ARR-3,blaNDM-7,blaOXA-1,blaOXA-10,blaOXY-2-8,catA2,catB3,cml,cmlA1,dfrA14,floR,mph(A),qacE,qnrS1,sul1,sul2
**17181**	Urine	2021	CA	*Enterobacter asburiae*	ST-252	aac(6')-Ib-cr,ant(3'')-Ia,ARR-3,blaACT-3,blaKPC-2,blaOXA-10,blaSHV-12,cml,cmlA1,dfrA14,floR,fosA,qacE,qnrE1,qnrS1,sul1,sul2,tet(D)
**17180**	Urine	2021	CA	*Enterobacter hormaechei*	ST-110	blaACT-15,blaKPC-2,fosA,qnrS1
**17179**	Urine	2021	CA	*Klebsiella pneumoniae*	ST15	aac(6')-Ib,aph(3'')-Ib,aph(6)-Id,ARR-3,blaCMY-6,blaCTX-M-15,blaNDM-1,blaSHV-106/28,blaTEM-1B,fosA6,oqxA,oqxB,qacE,rmtC,rmtF,sul1,sul2
17178	Urine	2021	CA	*Escherichia coli*	inconclusive	aac(6')-Ib-cr,aadA5,aph(3'')-Ib,aph(6)-Id,ARR-3,blaCTX-M-27,blaDHA-1,blaOXA-1,blaOXA-48,catB3,dfrA17,fosA3,mdf(A),mph(A),qacE,sitABCD,sul1,sul2,tet(A)
17836	Blood	2022	IL	*Acinetobacter baumannii*	ST2	aph(3')-Ia,aph(3'')-Ib,aph(6)-Id,blaADC-25,blaOXA-24,blaOXA-66,mph(E),msr(E),tet(B)
17176	Urine	2021	CA	*Pseudomonas aeruginosa*	ST644	aac(6')-Ib,aac(6')-Ib-cr,aadA11,aadA15,aadA2,ant(3'')-Ia,aph(3'')-Ib,aph(3')-IIb,aph(3')-VIa,aph(6)-Id,ARR-8,blaCARB-2,blaIMP-62,blaNDM-1,blaOXA-486,blaPAO,blaPME-1,catB7,cml,cmlA1,crpP,fosA,qacE,qnrVC1,sul1,tet(G)
17175	Urine	2021	KS	*Escherichia coli*	ST8	aph(3'')-Ib,aph(6)-Id,blaCTX-M-14,mdf(A),sul2
17174	Urine	2021	KS	*Pseudomonas aeruginosa*	ST2053	aac(6')-Ib-cr,aph(3')-IIb,blaOXA-396/494,blaPDC-386,catB7,fosA,sul2
**17173**	Urine	2021	KS	*Klebsiella pneumoniae*	ST2546-like	aac(3)-IIa,aac(6')-Ib-cr,aph(3'')-Ib,aph(6)-Id,blaCTX-M-15,blaOXA-1,blaSHV-187,blaTEM-1B,catB3,dfrA14,fosA,OqxA,OqxB,qnrB1,sul2

Table S2. Genotypic Results

Cepheid ID	Sample type	Year	State	Organism by K-mer spectra	MLST	Resistance genes identified
17172	Urine	2021	KS	*Escherichia coli*	ST254	blaCTX-M-15,sitABCD,mdf(A),qnrS1
