# Supplementary figures and images for: Characterization of Carbapenemase- and ESBL-Producing Gram-Negative Bacilli Isolated from Patients with Urinary Tract and Bloodstream Infections
Source: Antibiotics (Basel). 2023 Aug 30;12(9):1386. doi: 10.3390/antibiotics12091386 (PMC10525328; doi:10.3390/antibiotics12091386)

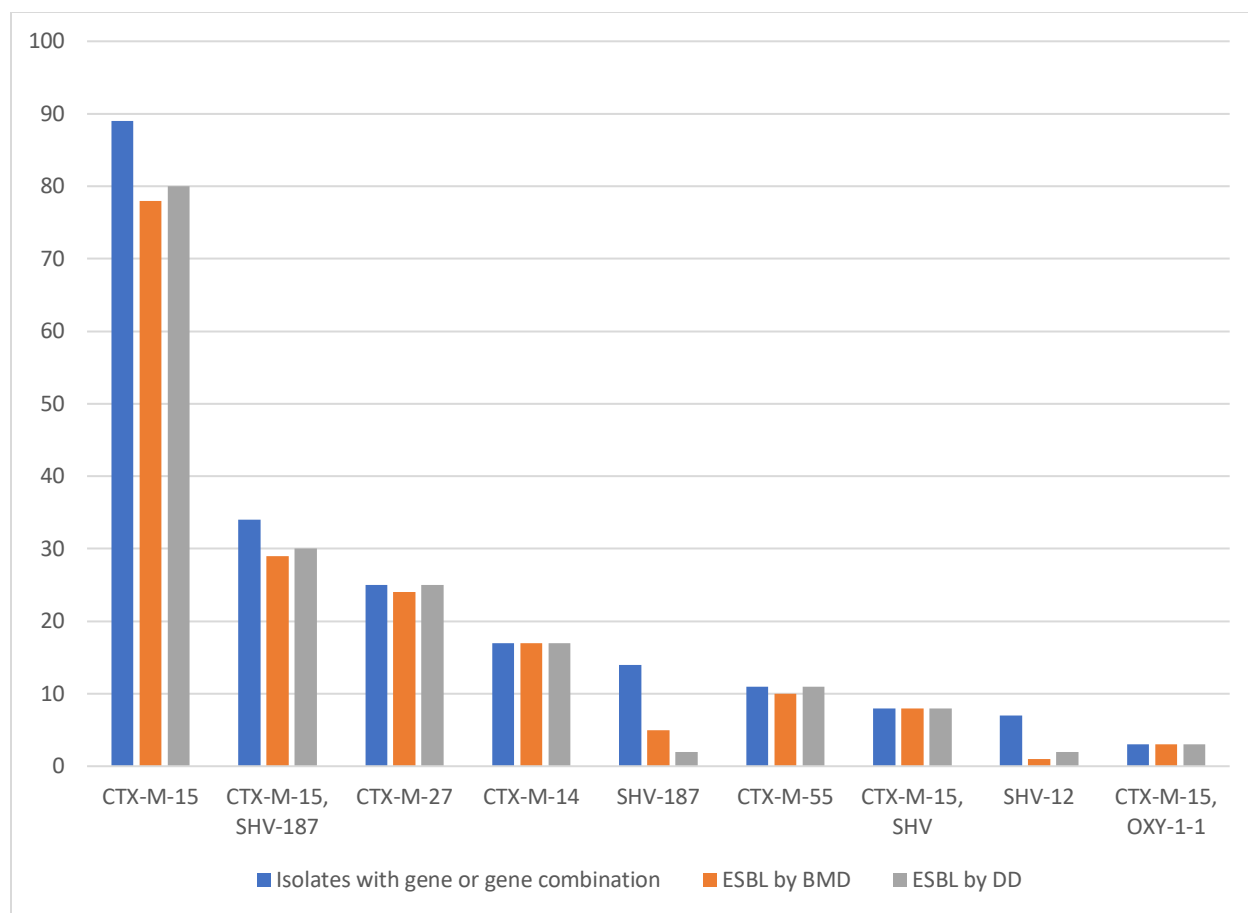

Figure S4. Most frequent ESBL genes identified and phenotypic identification by BMD and DD

Supplement: Supplementary file 1 [file antibiotics-12-01386-s001.zip › Figure S4.pdf]
